# Effect of a Comprehensive Cardiovascular Risk Reduction Intervention in Persons With Serious Mental Illness: A Randomized Clinical Trial

**DOI:** 10.1001/jamanetworkopen.2020.7247

**Published:** 2020-06-12

**Authors:** Gail L. Daumit, Arlene T. Dalcin, Faith B. Dickerson, Edgar R. Miller, A. Eden Evins, Corinne Cather, Gerald J. Jerome, Deborah R. Young, Jeanne B. Charleston, Joseph V. Gennusa, Stacy Goldsholl, Courtney Cook, Ann Heller, Emma E. McGinty, Rosa M. Crum, Lawrence J. Appel, Nae-Yuh Wang

**Affiliations:** 1 Division of General Internal Medicine, Department of Medicine, Johns Hopkins University School of Medicine, Baltimore, Maryland; 2 Welch Center for Prevention, Epidemiology and Clinical Research, Johns Hopkins University, Baltimore, Maryland; 3 Department of Psychiatry and Behavioral Sciences, Johns Hopkins University School of Medicine, Baltimore, Maryland; 4 Department of Epidemiology, Johns Hopkins Bloomberg School of Public Health, Baltimore, Maryland; 5 Department of Health Policy and Management, Johns Hopkins Bloomberg School of Public Health, Baltimore, Maryland; 6 Department of Mental Health, Johns Hopkins Bloomberg School of Public Health, Baltimore, Maryland; 7 Sheppard Pratt Health System, Towson, Maryland; 8 Department of Psychiatry, Massachusetts General Hospital, Boston,; 9 Department of Kinesiology, Towson University, Towson, Maryland; 10 Department of Research and Evaluation, Kaiser Permanente Southern California, Pasadena; 11 Department of Biostatistics, Johns Hopkins Bloomberg School of Public Health, Baltimore, Maryland

## Abstract

**Importance:**

Persons with serious mental illness have a cardiovascular disease mortality rate more than twice that of the overall population. Meaningful cardiovascular risk reduction requires targeted efforts in this population, who often have psychiatric symptoms and cognitive impairment.

**Objective:**

To determine the effectiveness of an 18-month multifaceted intervention incorporating behavioral counseling, care coordination, and care management for overall cardiovascular risk reduction in adults with serious mental illness.

**Design, Setting, and Participants:**

This randomized clinical trial was conducted from December 2013 to November 2018 at 4 community mental health outpatient programs in Maryland. The study recruited adults with at least 1 cardiovascular disease risk factor (hypertension, diabetes, dyslipidemia, current tobacco smoking, and/or overweight or obesity) attending the mental health programs. Of 398 participants screened, 269 were randomized to intervention (132 participants) or control (137 participants). Data collection staff were blinded to group assignment. Data were analyzed on the principle of intention to treat, and data analysis was performed from November 2018 to March 2019.

**Interventions:**

A health coach and nurse provided individually tailored cardiovascular disease risk reduction behavioral counseling, collaborated with physicians to implement appropriate risk factor management, and coordinated with mental health staff to encourage attainment of health goals. Programs offered physical activity classes and received consultation on serving healthier meals; intervention and control participants were exposed to these environmental changes.

**Main Outcomes and Measures:**

The primary outcome was the change in the risk of cardiovascular disease from the global Framingham Risk Score (FRS), which estimates the 10-year probability of a cardiovascular disease event, from baseline to 18 months, expressed as percentage change for intervention compared with control.

**Results:**

Of 269 participants randomized (mean [SD] age, 48.8 [11.9] years; 128 men [47.6%]), 159 (59.1%) had a diagnosis of schizophrenia or schizoaffective disorder, 67 (24.9%) had bipolar disorder, and 38 (14.1%) had major depressive disorder. At 18 months, the primary outcome, FRS, was obtained for 256 participants (95.2%). The mean (SD) baseline FRS was 11.5% (11.5%) (median, 8.6%; interquartile range, 3.9%-16.0%) in the intervention group and 12.7% (12.7%) (median, 9.1%; interquartile range, 4.0%-16.7%) in the control group. At 18 months, the mean (SD) FRS was 9.9% (10.2%) (median, 7.7%; interquartile range, 3.1%-12.0%) in the intervention group and 12.3% (12.0%) (median, 9.7%; interquartile range, 4.0%-15.9%) in the control group. Compared with the control group, the intervention group experienced a 12.7% (95% CI, 2.5%-22.9%; *P* = .02) relative reduction in FRS at 18 months.

**Conclusions and Relevance:**

An 18-month behavioral counseling, care coordination, and care management intervention statistically significantly reduced overall cardiovascular disease risk in adults with serious mental illness. This intervention provides the means to substantially reduce health disparities in this high-risk population.

**Trial Registration:**

ClinicalTrials.gov Identifier: NCT02127671

## Key Points

**Question:**

Does an 18-month intervention incorporating behavioral counseling, care coordination, and care management reduce cardiovascular risk in adults with serious mental illness, a population at extremely high risk of cardiovascular disease morbidity and mortality?

**Findings:**

In this randomized clinical trial enrolling 269 participants with serious mental illness and at least 1 cardiovascular risk factor, the intervention group participants had a 12.7% relative reduction in the 10-year probability of a cardiovascular event, compared with the control group.

**Meaning:**

These findings support the use of a behavioral counseling, care coordination, and care management intervention to substantially reduce cardiovascular health disparities in this high-risk population.

## Teaser

This randomized clinical trial examines the effectiveness of an 18-month multifaceted intervention incorporating behavioral counseling, care coordination, and care management for overall cardiovascular risk reduction in adults with serious mental illness.

## Introduction

Persons with serious mental illness, such as schizophrenia, bipolar disorder, and major depressive disorder, encompass a high-risk group, with a cardiovascular mortality rate more than twice that of the overall population and increased prevalence of all modifiable cardiovascular risk factors and behaviors.^1,2,3,4,5,6,7,8,9,10^ This population urgently needs interventions to address their multiple co-occurring risk factors, including diabetes, hypertension, dyslipidemia, tobacco smoking, and obesity.^11^ Because persons with serious mental illness often face complex issues, including persistent psychiatric symptoms, executive function impairment, social isolation, poverty, and substance use, tailored health interventions addressing these barriers are needed.^12,13,14,15,16^

Although behavioral weight loss and tobacco smoking cessation interventions adapted for persons with serious mental illness have shown efficacy in randomized trials,^17,18,19,20,21^ there is a dearth of evidence on comprehensive programs that target their multiple cardiovascular risk factors. To date, despite increasing interest in integrating physical health care into the mental health settings where many people with serious mental illness receive most of their care,^22^ studies of interventions in which specialty mental health organizations are responsible for physical health care coordination and management have shown either no or minimal improvements in cardiovascular risk factors.^23,24,25,26,27^ In contrast, care management and care coordination interventions have shown efficacy in improving cardiovascular risk factors in the overall population.^28,29^

In 2010, the American Heart Association established strategic goals to decrease cardiovascular disease mortality by 20% and improve overall cardiovascular health to ideal levels by 2020 for all Americans.^30^ Unless effective interventions are identified and implemented for persons with serious mental illness, it is likely that this population will continue to lag behind the nation in cardiovascular health. The purpose of our trial was to determine the effectiveness of an 18-month comprehensive intervention for overall cardiovascular risk reduction in adults with serious mental illness.

## Methods

### Study Design

We conducted a randomized clinical trial in 4 outpatient psychiatric rehabilitation programs and affiliated clinics within a large community mental health organization in Maryland. Institutional review boards at Johns Hopkins University and Sheppard Pratt Health System and an independent data safety and monitoring board approved the protocol (Supplement 1). All participants provided written informed consent after receiving a complete description of the study. This trial follows the Consolidated Standards of Reporting Trials (CONSORT) reporting guideline.

### Participants

Adults (aged ≥18 years) with hypertension, diabetes, dyslipidemia, current tobacco smoking, and/or overweight or obesity were eligible. Broad eligibility criteria aimed to enroll a population representative of persons with serious mental illness attending community mental health programs. Exclusion criteria were minimal and mainly related to safety (eTable 1 in Supplement 2). Study staff recruited participants through presentations at study sites and referrals from mental health program staff.

### Randomization and Masking

Individual participants were randomly assigned to intervention or control groups. The trial statistician computer-generated randomization allocation sequences. Randomization was stratified by study site and sex; assignments were generated with variable block sizes of 4 and 2. The study coordinator received the randomization assignment and communicated the group assignment to each participant. Participants and intervention staff were aware of group assignment; however, data collection staff and investigators were masked until trial end.

### Intervention Group

The intervention design drew from behavioral self-management concepts and social cognitive theory.^31,32^ To promote health behavior change, the intervention team used motivational interviewing and solution-focused therapy.^33,34^ Sessions were tailored to minimize the impact of memory and executive function deficits by breaking concepts into small components, repeating topics, and modeling and practicing skills.^35^ The design also incorporated approaches from care coordination and care management.^36,37^

The multifaceted intervention was delivered by heath coaches and a nurse and consisted of individually tailored cardiovascular risk reduction education and counseling sessions, collaboration with physicians for evidence-based management of cardiovascular health risk, and coordination with mental health staff to advocate for and encourage participants to reach individual cardiovascular health goals (eAppendix in Supplement 2).^21,38,39,40,41,42,43,44^ Intervention target goals were adapted from the American Heart Association’s Life’s Simple 7.^45^

Health coaches were based at the community mental health organization where they conducted individual-level coaching sessions. Sessions were 20 to 30 minutes long and were held weekly for the first 6 months and at least every 2 weeks thereafter. For participants with multiple risk factors, the participant and health coach collaboratively identified the primary focus for each session. The health coaches considered the status of each risk factor to guide discussion and used a patient-centered approach to help the participant identify the most impactful behavior change on which to focus between sessions. The health coaches’ location at the community mental health organization provided frequent opportunities for informal contacts, including follow-up with participants on progress toward selected behavioral change targets and communication with mental health program staff to support health goals. The health coaches interacted regularly with the nurse to collaborate on optimal care for each participant’s cardiovascular risk factors.

The nurse met with participants who required cardiovascular risk factor education (eg, diabetes or hypertension) and medication-related counseling. The nurse joined the participant as needed on selected physician visits to advocate for evidence-based treatment and monitoring for diabetes, hypertension, dyslipidemia, and tobacco dependence. The nurse’s interaction with physicians’ and office staff also included sharing information on participant risk factors, facilitating scheduling of and follow-up for appointments and laboratory testing, and communication with other health care practitioners as needed. The health coaches assisted in coordination efforts, including communication with mental health staff and caregivers around participant cardiovascular health.

The intervention supported individual participants’ goals with a point system to reward session attendance and to incentivize recommended behavior change. For example, to encourage smoking cessation, individuals trying to quit received points for reduction in expired carbon monoxide concentration.^46^ Points were exchanged for small reward items.

Study staff received initial and follow-up training, including regular observation for quality assurance. Implementation of standardized materials and procedures supported intervention fidelity. In addition to the individual-level intervention, we encouraged changes in site environments to support healthy behaviors.

We partnered with the community mental health organization and provided resources and training for them to deliver group physical activity classes for all program attendees, regardless of study enrollment. A study dietician also consulted with program kitchen staff to recommend healthier breakfast and lunch options at sites.

### Control Group

As mental health program attendees, control group participants were exposed to the aforementioned environmental changes. They did not receive an individual-level intervention.

### Outcomes

The prespecified, primary outcome was the change in the risk of a cardiovascular event from the global Framingham Risk Score^47^ from baseline to 18 months, expressed as a percentage change for the intervention group compared with the control group. The global Framingham Risk Score estimates the 10-year probability of a cardiovascular disease event (coronary heart disease, cerebrovascular event, peripheral artery disease, or heart failure). The Framingham Risk Score is used in primary care to assess overall cardiovascular risk, providing clinicians with quantitative information to aid in targeted lowering of risk factors.^48,49,50^ We used relative change in the Framingham Risk Score because it corresponded to the American Heart Association’s goal to reduce cardiovascular mortality by 20%.^30^ Other study outcomes included changes in the global Framingham Risk Score at 6 months and individual modifiable score components at 6 and 18 months (systolic blood pressure, total cholesterol, high-density lipoprotein, diabetes status, and tobacco smoking status), as well as diastolic blood pressure, fasting blood glucose, glycated hemoglobin A_1C_, low-density lipoprotein, triglycerides, and body mass index (calculated as weight in kilograms divided by height in meters squared).

### Data Collection

Data collection staff performed assessments in person. Blood pressure, body mass index, fasting blood chemical levels, exhaled carbon monoxide, and other study variables including surveillance for safety and adverse events were obtained at baseline and at 6 and 18 months. For tobacco smoking, cessation was determined by self-report and was confirmed by exhaled carbon monoxide level less than 7 ppm.^51,52^ Sociodemographic characteristics and medication information were obtained from participant self-report and program records, and psychiatric diagnoses were abstracted from program records. Follow-up data collection was completed in November 2018.

### Statistical Analysis

Analyses were conducted according to the intention to treat principle. The primary analysis was conducted through a likelihood-based repeated measures mixed-effects regression model with mean of log-transformed global Framingham Risk Score as a function of intervention group assignment, study visit indicators (6 and 18 months, with baseline as reference), and intervention-by-visit interaction terms as the main model predictors, adjusting for sex and study site. A 3-by-3 unstructured variance-covariance matrix was used for the 3 repeated outcome measures per participant. All data were used in the analysis, with any missing data included using the statistical software’s designated missing indicator and treated as missing at random in modeling. The same modeling approach was used to examine the continuous variables for secondary outcomes and to explore the heterogeneity of treatment effects on the primary outcome among prespecified subgroups, including sex, race, cardiovascular risk score at baseline, and psychiatric diagnosis, by incorporating appropriate 2-way and 3-way interaction terms between main model predictors and subgroup indicators. The intervention’s effect on relative change of the American College of Cardiology and American Heart Association Cardiovascular Disease Risk Score^53^ was also explored using this approach. Generalized estimating equations were used to estimate the population mean treatment effects on binary variables among the risk score components (ie, hypertension medication use, diabetes, and smoking status) over time. Mean models incorporated the same predictors as primary outcome analyses, with statistical inferences based on robust standard errors using the unstructured working correlation. For mixed-effects modeling, *t* and *F* tests were used, and *Z* and χ^2^ tests were used in generalized estimating equation modeling results. All tests were 2-sided, with *P* < .05 considered statistically significant. SAS statistical software version 9.4 (SAS Institute) was used for analyses. Data analysis was performed from November 2018 to March 2019. The enrollment target was 250 participants, with assumed 10% loss to follow-up, a baseline global Framingham Risk Score of 11%, and relative risk reduction in the control group of 5%, providing 80% power to detect a 10.7% reduction in the ratio between intervention and control groups of change in relative global Framingham Risk Score at 18 months.

## Results

### Study Participants

**Figure 1.**
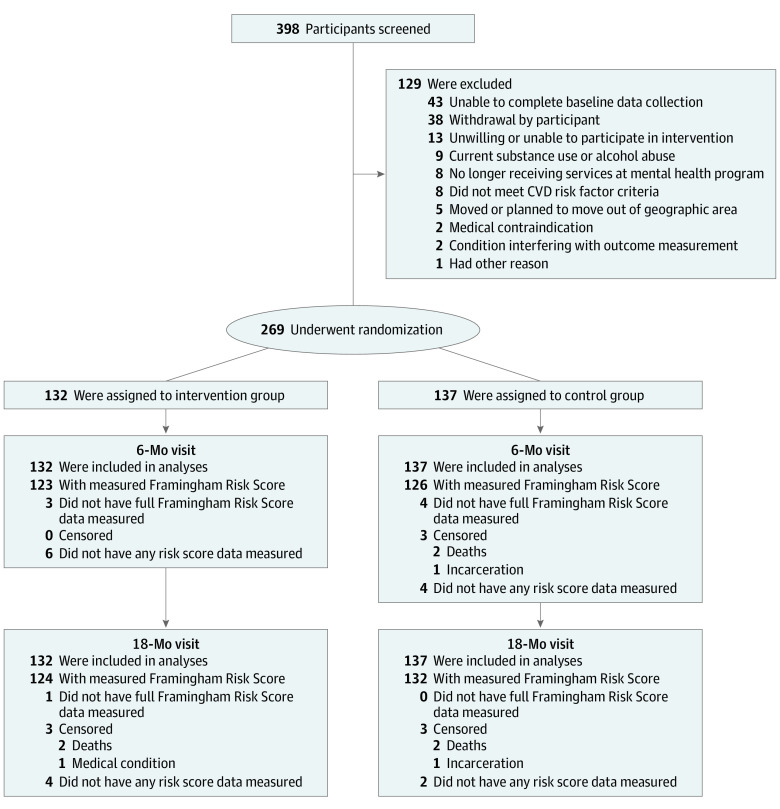
Screening, Randomization, and Follow-up of Study Participants CVD indicates cardiovascular disease.

Three hundred ninety-eight participants were screened, and 269 underwent randomization: 132 participants were randomized to the intervention group, and 137 participants were randomized to the control group (Figure 1). The mean (SD) age was 48.8 (11.9) years; 128 participants (47.6%) were men, and 124 participants (46.1%) were black (Table 1). Two hundred thirty-two participants (86.2%) were unable to work or had disability, and 153 (56.9%) lived in supported housing. A total of 159 (59.1%) had schizophrenia or schizoaffective disorder, 67 (24.9%) had bipolar disorder, and 38 (14.1%) had major depressive disorder. The mean (SD) number of psychotropic medications was 3.6 (1.9). Two hundred forty-two participants (90%) had overweight or obesity, 142 (52.8%) had hypertension, 93 (34.6%) had diabetes, 175 (65.1%) had dyslipidemia, and 138 (51.3%) smoked tobacco at baseline, with more than 85% having 2 or more risk factors for cardiovascular disease. Complete measures comprising the global Framingham Risk Score were obtained from 256 participants (95.2%) at 18 months.

**Table 1.** Baseline Characteristics of the Study Participants

Characteristic	Participants, No. (%)	
	Intervention (n = 132)	Control (n = 137)
Age, mean (SD), y	48.5 (10.8)	49.1 (12.9)
Male	62 (47.0)	66 (48.2)
Race		
White	67 (50.8)	68 (49.6)
Black or African American	61 (46.2)	63 (46.0)
Asian	1 (0.8)	4 (2.9)
Native Hawaiian or other Pacific Islander	1 (0.8)	0
Other race	2 (1.5)	2 (1.5)
Hispanic or Latino	2 (1.5)	4 (2.9)
Not high school graduate	29 (22.0)	37 (27.0)
Never married	98 (74.2)	85 (62.0)
Lives in residential program or with caregiver	76 (57.6)	77 (56.2)
Unable to work or receiving disability	112 (84.9)	120 (87.6)
Health insurance	130 (98.5)	135 (98.5)
Medicaid	125 (94.7)	129 (94.2)
Medicare	68 (51.5)	67 (48.9)
Regular physician	126 (95.5)	129 (94.2)
Routine physical examination in the past year	113 (85.6)	122 (92.4)
Psychiatric diagnoses		
Schizophrenia	40 (30.3)	41 (29.9)
Schizoaffective disorder	46 (34.9)	32 (23.4)
Bipolar disorder	25 (18.9)	42 (30.7)
Major depression	20 (15.2)	18 (13.1)
Other psychotic disorder	1 (0.8)	4 (2.9)
History of alcohol or other substance use disorder^a^	69 (52.3)	69 (50.4)
All medications, mean (SD), No.	9.4 (5.7)	10.4 (5.4)
Psychotropic medications, mean (SD), No.	3.5 (1.9)	3.6 (1.8)
Antipsychotic		
Any	106 (80.3)	114 (83.2)
Second generation	86 (65.2)	103 (75.2)
Clozapine or olanzapine	29 (22.0)	34 (24.8)
Lithium or mood stabilizer	68 (51.5)	82 (59.9)
Antidepressant	86 (65.2)	88 (64.2)
Psychiatric measures, mean (SD), score		
Behavior and Symptom Identification Scale–24^b^	1.1 (0.7)	1.2 (0.7)
Center for Epidemiologic Studies Depression Scale^c^	20.8 (11.8)	19.9 (12.0)
Cardiovascular risk factors, No.^d^		
1	14 (10.6)	20 (14.6)
2	37 (28.0)	23 (16.8)
3	39 (29.6)	47 (34.3)
4	33 (25.0)	34 (24.8)
5	9 (6.8)	13 (9.5)

^a^ Determined according to the Behavior and Symptom Identification Scale–24, the Addiction Severity Index, and diagnoses captured during medical chart abstraction.

^b^ Overall summary scores on the Behavior and Symptom Identification Scale–24 range from 0 to 4, with higher scores indicating greater severity of symptoms.

^c^ Scores on the Center for Epidemiologic Studies Depression Scale range from 0 to 60, with higher scores indicating more depressive symptoms; a score of 16 points is considered to be a cutoff point for depression.

^d^ Includes body mass index (calculated as weight in kilograms divided by height in meters squared) greater than or equal to 25, hypertension, diabetes, dyslipidemia, and tobacco smoking.

### Cardiovascular Risk Reduction

The mean (SD) baseline 10-year global Framingham Risk Score (ie, the 10-year probability of a cardiovascular event) was 11.5% (11.5%) (median, 8.6%; interquartile range [IQR], 3.9% to 16.0%) in the intervention group and 12.7% (12.7%) (median, 9.1%; IQR, 4.0% to 16.7%) in the control group (eTable 2 in Supplement 2). At 18 months, cross-sectional estimates of mean (SD) risk scores based on observed data for the intervention and control groups were 9.9% (10.2%) (median, 7.7%; IQR, 3.1% to 12.0%) and 12.3% (12.0%) (median, 9.7%; IQR, 4.0% to 15.9%), respectively. The percentage relative reduction in risk score within the intervention group was 11.2% (95% CI, 3.9% to 18.5%; *P* = .003) from baseline at 18 months, whereas the percentage relative increase in risk from baseline was 1.4% (95% CI, −8.6% to 5.7%; *P* = .69) in controls at 18 months, according to mixed-effects analyses.

**Figure 2.**
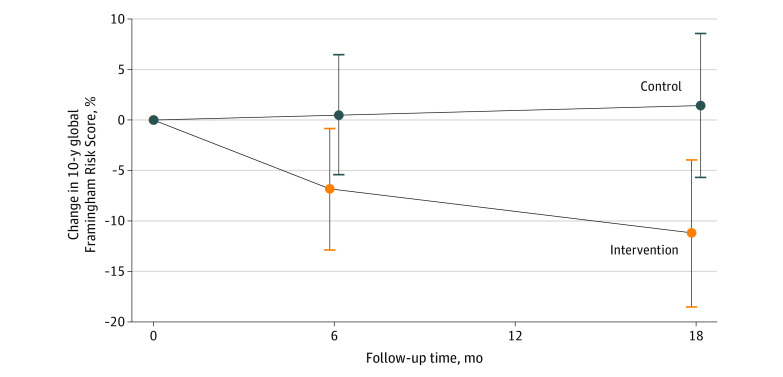
Percentage Change in 10-Year Global Framingham Risk Score Over Time According to Study Group The Global Framingham Risk Score reflects the 10-year probability of a cardiovascular event. The numbers of participants in the intervention group were 132 at baseline, 123 at 6 months, and 124 at 18 months. The numbers of participants in the control group were 137 at baseline, 126 at 6 months, and 132 at 18 months. Percentage change estimates (circles) and 95% CIs (error bars) are derived from mixed-effects repeated measures analysis using all available data from all randomized participants. Compared with the control group, the intervention group experienced a mean relative reduction in Framingham Risk Score of 12.7% (95% CI, 2.5%-22.9%; *P* = .02).

For the primary study outcome, the net percentage reduction in the 10-year global Framingham Risk Score for the intervention group compared with control at 18 months was 12.7% (95% CI, 2.5%-22.9%; *P* = .02) (Figure 2; eTable 2 in the Supplement 2), which would correspond to an absolute risk reduction of 1.5% and a number needed to treat of 66 in a population with our 12.1% baseline risk. Analyses with the American College of Cardiology and American Heart Association Risk Score showed similar results with net percentage reduction in 10-year cardiovascular risk of 13.2% (95% CI, 0.5%-25.8%; *P* = .04) for the intervention compared with control at 18 months (eFigure 1 and eTable 3 in Supplement 2). Results did not differ by subgroups of age, baseline cardiovascular risk, sex, race, and psychiatric diagnosis (all *P* for interaction >.05; eFigure 2 in Supplement 2).

### Individual Cardiovascular Risk Factors

Table 2 shows global Framingham Risk Score components and other relevant cardiovascular risk factors. Each risk score component measured in continuous scale (mean [SD] at 18 months in the intervention vs control group: systolic blood pressure, 116.5 [12.9] mm Hg vs 120.2 [16.2] mm Hg; total cholesterol, 171.8 [38.3] mg/dL vs 177.7 [48.7] mg/dL; and high-density lipoprotein levels, 50.7 [14.8] mg/dL vs 51.0 [17.7] mg/dL; to convert cholesterol to millimoles per liter, multiply by 0.0259) and the other continuous risk factor measures had a point estimate favoring the intervention compared with control at 18 months, although between-group differences were not statistically significant. For binary risk score components, absolute changes in prevalence for both diabetes and medication for hypertension also were both nonsignificant for intervention compared with control at 18 months. Tobacco smoking rates declined in the intervention group such that the absolute change in prevalence for those who smoked tobacco at 18 months relative to baseline was −11.81% (95% CI, −18.32% to −5.31%; *P* = .004) in the intervention group, compared with −1.32% (95% CI, −5.78% to 3.14%; *P* = .64) in controls, a −10.50% net change in prevalence (95% CI, −18.39% to −2.59%; *P* = .009) over 18 months. This translates to a 21% relative reduction in smoking prevalence over 18 months (ratio of prevalence ratios [intervention to control] = 0.79; 95% CI, 0.67 to 0.95; *P* = .01). Exploratory analyses suggest that although smoking cessation was a major contributor to risk reduction at 18 months, changes in blood pressure and lipids also affected overall risk reduction (data not shown).

**Table 2.** Outcomes for Individual Cardiovascular Risk Factors According to Study Group

Outcome	Intervention group			Control group			Between-group difference, mean (95% CI)^a^
	Mean (SD)		Change, mean (95% CI)^a^	Mean (SD)		Change, mean (95% CI)^a^	
	Baseline	18 mo		Baseline	18 mo		
Continuous outcomes							
Blood pressure, mm Hg							
Systolic^b^	118.1 (13.0)	116.5 (12.9)	−3.9 (−1.5 to 0.8)	120.1 (14.5)	120.2 (16.2)	0.2 (−2.1 to 2.5)	−1.8 (−5.1 to 1.5)
Diastolic	75.3 (8.2)	73.9 (9.2)	−3.0 (−1.5 to 0.1)	74.9 (9.3)	74.7 (11.1)	0.2 (−1.3 to 1.7)	−1.7 (−3.8 to 0.5)
Total cholesterol, mg/dL^b^	178.9 (42.1)	171.8 (38.3)	−7.7 (−14.2 to −1.3)	181.1 (41.6)	177.7 (48.7)	−3.4 (−9.7 to 2.9)	−4.3 (−13.3 to 4.7)
Low-density lipoprotein, mg/dL	101.9 (36.8)	93.7 (33.5)	−8.5 (−13.9 to −3.3)	101.0 (33.4)	97.3 (35.9)	−3.2 (−8.4 to 1.9)	−5.4 (−12.7 to 2.0)
High-density lipoprotein, mg/dL^b^	49.2 (15.3)	50.7 (14.8)	1.5 (−0.5 to 3.5)	50.2 (19.3)	51.0 (17.7)	0.5 (−1.4 to 2.4)	1.0 (−1.8 to 3.8)
Trigylcerides, mg/dL	140.2 (74.3)	141.0 (76.9)	0.6 (−14.8 to 16.0)	156.6 (106.7)	149.0 (111.1)	−7.5 (−22.5 to 7.6)	8.1 (−13.4 to 29.6)
Fasting glucose, mg/dL	106.5 (36.7)	106.7 (45.2)	0.5 (−6.7 to 7.6)	110.6 (36.9)	117.4 (60.0)	8.1 (1.1 to 15.0)	−7.6 (−17.6 to 2.4)
Glycated hemoglobin A_1C_, %	6.0 (1.2)	5.9 (1.3)	−0.1 (−0.3 to 0.1)	6.3 (1.4)	6.2 (1.8)	−0.0 (−0.2 to 0.1)	−0.0 (−0.3 to 0.2)
Body mass index^c^	34.4 (7.8)	33.7 (7.7)	−0.8 (−1.3 to −0.2)	32.9 (6.6)	32.1 (6.6)	−0.9 (−1.4 to −0.4)	0.1 (−0.7 to 0.9)
Binary outcomes							
Medication for hypertension, No. (%)^b^	63 (47.7)	70 (53.0)	7.99 (1.16 to 14.81)^d^	65 (47.5)	66 (48.2)	−1.87 (−5.04 to 8.80)^d^	6.11 (−3.61 to 15.84)^e^
Diabetes, No. (%)^b^	42 (31.8)	46 (34.9)	4.74 (1.11 to 8.36)^d^	51 (37.2)	60 (43.8)	8.51 (3.89 to 13.14)^d^	−3.78 (−9.65 to 2.10)^e^
Tobacco smoking, No. (%)^b^	65 (49.2)	47 (35.6)	−11.81 (−18.32 to −5.31)^d^	73 (53.3)	67 (48.9)	−1.32 (−5.78 to 3.14)^d^	−10.50 (−18.39 to −2.59)^e^

SI conversion factors: to convert cholesterol to millimoles per liter, multiply by 0.0259; glycated hemoglobin A_1C_ to proportion of total hemoglobin, multiply by 0.01; triglycerides to millimoles per liter, multiply by 0.0113; and glucose to millimoles per liter, multiply by 0.0555.

^a^ Mixed-effects repeated measures model-based estimates.

^b^ Components of the Global Framingham Risk Score.

^c^ Body mass index is calculated as weight in kilograms divided by height in meters squared.

^d^ Data are absolute change in prevalence, mean (95% CI), calculated with generalized estimating equations–based population-average estimates.

^e^ Data are net change in prevalence, mean (95% CI), calculated with generalized estimating equations–based population-average estimates.

### Intervention Participation

Table 3 displays the median number of intervention contacts for intervention group participants. Health coach sessions were recommended weekly for the first 6 months and then biweekly for months 7 to 18 (51 sessions); the median number of sessions completed over the 18-month intervention was 38 (IQR, 27-49 sessions). Nurse sessions were conducted as needed with participants who had hypertension, diabetes, dyslipidemia or were interested in pharmacotherapeutic smoking cessation aids with a median of 3.5 sessions (IQR, 2-7 sessions). Among participants for whom the nurse accompanied them to a physician appointment, the median number of appointments was 2 (IQR, 1-3 sessions).

**Table 3.** Types of Contacts, by Study Period, in the Intervention Group

Contact type	Period		
	1-6 mo	7-18 mo	1-18 mo
Health coach behavioral counseling sessions^a^			
Participants with contacts, No.	130	123	131
Sessions, median (IQR), No.	20 (14-24)	20 (10-26)	38 (27-49)
Nurse contacts^b^			
Nurse-participant sessions			
Participants with contacts, No.	68	50	84
Sessions, median (IQR), No.	3 (2-5.5)	2 (1-3)	3.5 (2-7)
In-person physician visits with study nurse			
Participants with contacts, No.	46	65	79
Visits, median (IQR), No.	1 (1-2)	1 (1-3)	2 (1-3)
Study nurse contacts with physician or office staff by telephone, email, or fax			
Participants with contacts, No.	42	95	98
Contacts, median (IQR), No.	1 (1-2)	2 (1-4)	3 (1-5)

Abbreviation: IQR, interquartile range.

^a^ Medians and IQRs are based on 132 participants.

^b^ Medians and IQRs are based on participants with contacts.

### Adverse Events

There were 2 deaths each in the intervention and control groups. No medical events were considered probably or definitely study related. Fifteen percent of intervention participants and 22% of controls reported a medical hospitalization, and 8% of participants in the intervention group and 7% of participants in the control group reported a psychiatric hospitalization (eTable 4 in the Supplement 2).

## Discussion

In adults with serious mental illness attending community mental health programs, an 18-month behavioral counseling, care coordination, and care management intervention statistically significantly reduced the estimated 10-year risk of a cardiovascular disease event measured by the global Framingham Risk Score. Rates of tobacco smoking were statistically significantly reduced in the intervention group compared with the control group, and although between-group differences were not statistically significant, the direction of change for blood pressure and lipid risk score components were toward improvement in the intervention group.

The magnitude of estimated overall cardiovascular risk reduction in this trial is substantial and comparable to prior studies in the general population.^29,54,55,56,57,58^ Our observed relative risk reduction of 12.7% corresponds to an absolute risk reduction of 1.5% in a population with our 12.1% baseline risk. Our study included a broad range of participants with any cardiovascular risk factor, including those with controlled risk factors; in populations with higher baseline risk, we would expect the intervention to produce even greater absolute risk reduction. Still, the level of cardiovascular risk reduction in this trial corresponds to a number needed to treat of 66, which is in range with trials of antihypertensive therapy and primary prevention with statins.^59,60,61,62^ Exploratory analyses suggest that although smoking cessation was a major contributor to risk reduction at 18 months, changes in blood pressure and lipids also affected overall risk reduction.

In contrast to our findings, care coordination interventions to date have not resulted in cardiovascular risk improvements for persons with serious mental illness, despite a widespread increase in programs incorporating physical health programs into mental health settings.^26^ In the US, recent studies evaluated behavioral health homes where primary care coordination and management are embedded in specialty mental health settings; of the 2 randomized trials,^23,24^ 1 showed no effects on cardiovascular risk factors, and 1 showed small decreases in risk among those with laboratory data in the intervention group compared with the control group. In Denmark, the CHANGE trial^25^ tested care coordination plus lifestyle coaching compared with care coordination and control with null results. The low-intensity lifestyle coaching in the studies’ interventions and the Danish health system’s high quality may explain the lack of improvement in cardiovascular risk in previous trials.

### Strengths and Limitations

Our trial has several strengths and important features. First, the study enrolled a diverse group of adults with serious mental illness from community-based programs with a range of diagnoses and psychotropic regimens. Despite their young age, participants composed a high-risk group; more than 85% of participants had at least 2 cardiovascular risk factors. Second, we achieved very high follow-up rates for outcome data. Third, the intervention incorporated strategies to address multiple cardiovascular risk factors to facilitate health behavior change. Fourth, having health coaches embedded in the community mental health programs approximated real-world implementation.

Our trial also has limitations. First, the outcome was a risk score rather than actual clinical events. However, risk scores are considered meaningful proxies of clinical outcomes, are feasible in trial settings, and are used to estimate intervention effects in research and clinical practice.^29,50,55,56,57,58,63^ Second, the Framingham Risk Score has limitations (eg, it was developed in a predominantly white population); however, it was the most widely used score available at trial initiation, and we found similar results using the newer American College of Cardiology and American Heart Association risk score. Third, the trial was designed with power to detect the primary outcome, with only limited power for changes in individual cardiovascular risk factors. Fourth, although the intervention was embedded in a community mental health organization and the intervention team worked closely with mental health staff and practitioners, there was no formal relationship with primary care practitioners, and the intervention team could not prescribe medications. Thus, although the nurse advocated for evidenced-based treatment, it is possible that, in a more formal integrated care structure, the intervention could have resulted in even greater risk reduction.

## Conclusions

Results from our trial support the use of a tailored intervention to address cardiovascular risk factors, embedded in routine outpatient specialty mental health care for adults with serious mental illness. In the US, through the Affordable Care Act Medicaid Health Home Waiver, behavioral health homes placing primary care coordination and management in specialty mental health settings have been implemented in 17 states and the District of Columbia.^26^ These health homes or similar integrated care programs could be a vehicle for implementing the intervention. Organizations may choose to target persons with serious mental illness with higher baseline cardiovascular risk.

These findings show that a behavioral counseling, care coordination, and care management intervention embedded in routine outpatient specialty mental health care can significantly reduce overall cardiovascular risk in adults with serious mental illness. This intervention provides the means to substantially reduce health disparities in this high-risk population.

## Notes

Supplement 1. **Trial Protocol**

Supplement 2. **eAppendix.** Synopsis of Intervention **eTable 1.** Trial Enrollment Criteria **eTable 2.** Global Framingham Risk Score by Randomized Group at Each Visit (Cross-Sectional) and Model-Based Percent Longitudinal Change of Global Framingham Risk Score Over Time **eFigure 1.** Percent Change in ACC/AHA Risk Score Over Time According to Study Group **eTable 3.** ACC/AHA Risk Score by Randomized Group at Each Visit and Model-Based Percent Longitudinal Change of ACC/AHA Risk Score Over Time **eFigure 2.** Model-Based Percent Change in Global Framingham Risk Score by Subgroups of Sex, Race, Cardiovascular Risk at Baseline, and Psychiatric Diagnosis **eTable 4.** Overnight Hospitalizations, Deaths and Medical Events for Intervention and Control Participants

Supplement 3. **Data Sharing Statement**
