# Supplementary material for: Effect of a Comprehensive Cardiovascular Risk Reduction Intervention in Persons With Serious Mental Illness: A Randomized Clinical Trial
Source: JAMA Netw Open. 2020 Jun 12;3(6):e207247. doi: 10.1001/jamanetworkopen.2020.7247 (PMC7293000; doi:10.1001/jamanetworkopen.2020.7247)
Supplement: Supplement 1. — Trial Protocol [file jamanetwopen-3-e207247-s001.pdf]

Comprehensive Cardiovascular Risk Reduction Trial in Persons with Serious Mental Illness

Protocol notes

We had initially submitted the protocol in an IRB-specified format in August, 2013 and then reformatted it in September, 2018 to an investigator-initiated clinical trial protocol format for ease of readability by reviewers and the wider scientific community. The protocol text is the same as the original IRB-specified format with the updates below. We are submitting both the original protocol in the IRB-specified format and the reformatted, updated protocol.

Edits to the protocol, all made prior to data analysis, are as follows:

i. Clarified the number of blood pressure measurements obtained at each data collection point
ii. Clarified 24-month data, serum carotenoids and accelerometer data will not be collected
iii. Updated background literature
iv. Provided more detail on definition of control and goals for hypertension, diabetes and dyslipidemia
v. Clarified and provided more detail for definition of outcome for smoking status
vi. Clarified intervention description including updating table formatting, titles of staff (e.g., health coach instead of heart health interventionist), clearer wording
vii. Provided more detail on data analysis plan including exploratory analyses, and specifically including analysis of AHA/ACC ASCVD risk score, which became available during the course of the trial.
viii. Data management, quality assurance plans included
ix. Recruitment and consent information in online IRB application now included in protocol text

STUDY PROTOCOL

COMPREHENSIVE CARDIOVASCULAR DISEASE RISK REDUCTION TRIAL IN PERSONS WITH SERIOUS MENTAL ILLNESS (IDEAL)

September 3, 2018

Table of Contents

1. ABSTRACT 3

2. SPECIFIC AIMS 3

3. BACKGROUND AND SIGNIFICANCE 4

4. DESIGN SUMMARY 6

5. STUDY POPULATION AND ELIGIBILITY 7

6. RECRUITMENT 7

7. DATA COLLECTION AND MEASUREMENTS 8

8. QUALITY ASSURANCE AND QUALITY CONTROL 11

9. RANDOMIZATION AND BLINDING 11

10. INTERVENTION 11

11. DATA MANAGEMENT 17

12. DATA ANALYSIS 17

13. DATA SECURITY 21

14. SAFETY 21

15. REFERENCES 24

IDEAL Trial Protocol

1. ABSTRACT

Persons with serious mental illness (SMI), such as schizophrenia and bipolar disorder, die on average 25 years earlier than persons without SMI. Much premature death in SMI is attributable to CVD with CVD-related mortality rates twice those of the overall population. Accordingly, this vulnerable group, comprising at least 15 million Americans, has a high burden of modifiable CVD risk factors including smoking, obesity, hypertension, diabetes, and dyslipidemia. The American Heart Association (AHA) recently set ambitious strategic Impact Goals to improve the cardiovascular health of all Americans and reduce deaths from CVD by 20 percent by 2020. Substantial efforts are underway to meet these goals in the general population. Still, interventions need to be adapted for persons with SMI who often have substantial barriers related to cognitive impairment and psychiatric symptoms. Hence, without special efforts to promote CV health and control risk factors in vulnerable populations like the SMI, it is unlikely that CV health will improve in SMI patients, and disparities in CV health will not only persist, but likely worsen. In view of their extraordinary burden of CVD risk factors, the overarching objective of this protocol is to develop and test a scalable intervention that could be widely adopted to reduce risk in this vulnerable population. Our multidisciplinary research team has expertise designing and rigorously testing interventions to decrease CVD risk in the general population and in persons with SMI.

The IDEAL Trial is a community organization-based, two-arm clinical trial that will test the hypothesis that an 18-month comprehensive, practical CVD risk reduction program will be superior to control in improving CV health in persons with SMI. We will partner with Mosaic Community Services, Inc., a large mental health care organization, and enroll a target of 250 persons with SMI who attend 4 psychiatric rehabilitation day programs (PRPs) or adjoining mental health clinics. Rehabilitation programs provide opportune settings to implement CVD risk reduction programs because patients attend several days a week and environmental components can take place on-site. We will provide a common background of exercise classes and healthy changes to meals served. Participants will then be randomized to receive this control condition or the 18-month active IDEAL intervention with a Heart Health Interventionist, who will provide on-site: 1) individual CVD risk reduction behavioral counseling (e.g., for smoking cessation, diet); 2) coordination with primary care providers to ensure appropriate management of risk factors (e.g., blood pressure control); and 3) collaboration with mental health staff and social supports to encourage and motivate participants to reach goals. The primary outcome will be the change in estimated CVD risk from the global Framingham Risk Score; secondary outcomes will include AHA metrics for CV health. Accomplishing meaningful reductions in CVD throughout the population will require targeted interventions focusing on vulnerable, high-risk groups such as those with SMI. If successful, this trial will provide persuasive evidence for use of a practical, comprehensive CVD risk reduction strategy in SMI to reduce health disparities.

2. SPECIFIC AIMS

Accomplishing meaningful reductions in CVD throughout the US population will require implementation of targeted interventions that focus on vulnerable, high-risk groups such as those with serious mental illness. In this context, we propose the following Specific Aims:

Primary Aim:

1. Test the hypothesis that the active IDEAL intervention is more effective than the control condition in reducing CVD risk using the global Framingham Risk Score (FRS).

Secondary Aims:

2. Determine the effect of the active intervention compared to control on AHA metrics for ideal CV health risk behaviors and health risk factors:
    a. Weight and BMI;
    b. Physical fitness with 6-minute walk test;
    c. Healthy diet measured by self-report;
    d. Smoking cessation with biochemical validation;
    e. Fasting plasma glucose levels and diabetes mellitus treated to goal (HgBA1c);

f. Blood pressure levels and hypertension treated to goal;
g. Lipid levels (total cholesterol, LDL-C, HDL-C, triglycerides) and dyslipidemia treated to goal;

3. Determine the effects of the active intervention on other outcomes: health status (SF-12); quality of life (Euroqol); medication use for CVD risk factors; and medication adherence.
4. Assess costs per participant and perform a cost effectiveness analysis.

Exploratory Aims:

5. Conduct analyses to identify potential mediator variables (e.g., intervention participation, medication use for CVD risk factors and adherence);
6. Assess organizational factors that could facilitate the most effective future dissemination.

The primary outcome variable will be reduction in CVD risk with global FRS from randomization to 18 months. Six-month FRS and 6 and 18-month changes in health risks comprise secondary outcomes.

3. BACKGROUND AND SIGNIFICANCE

Public Health Burden of Cardiovascular Disease in Persons with Serious Mental Illness

Persons with SMI, over 5 percent of the population or 15 million Americans, comprise a high-risk group for CVD-related mortality with rates approximately twice those of the overall US.[1-5] SMI frequently produces challenges in everyday functioning due to cognitive impairment and ongoing psychiatric symptoms;[6] accordingly, populations with SMI are often characterized by socioeconomic and environmental risk factors for CVD such as unemployment, low-income and social isolation.[7] The AHA Strategic Plan (co-authored by Dr. Appel) defines 7 goals for ideal CV health for all Americans including ideal health behaviors (BMI <25 kg/m^2, physical activity at goal levels, healthy diet, nonsmoking) and ideal health factors (fasting blood glucose <100mg/dl, untreated blood pressure <120/<80mmHg, untreated total cholesterol <200mg/dl).[8] Persons with SMI experience increased prevalence of unhealthy levels of these CVD risk factors, summarized below.

Overweight and obesity are at epidemic levels in SMI, particularly in women, where 60% are obese.[9-12] Physical inactivity and unhealthy diet contribute. The vast majority of SMI require ≥1 long-term psychotropic medication, yet many psychotropic classes cause weight gain, in part from increased appetite.[13-17] In particular, 2nd generation antipsychotics lead to substantial weight increases; in the case of olanzapine, up to 0.9 kg/mo.[18]

Physical inactivity is prevalent in SMI; Dr. Daumit reported persons with SMI report 50% higher leisure time inactivity than the general population.[19] An accelerometry study showed only 4% of persons with SMI in a psychiatric rehabilitation program (PRP) met recommended moderate to vigorous physical activity in bouts of ≥10 minutes.[20] Lack of affordable, safe places to exercise, and negative affective states such as depression may be important barriers.

Unhealthy diet is reported in persons with SMI with some studies reporting higher fat and lower fruit and vegetable intake and others show higher overall caloric intake.[21-23] Persons with SMI are also likely to depend on others for meals; preliminary baseline data from Dr. Daumit's study show 40% of participants relying on others to buy or prepare food when they eat at home.

Tobacco smoking is extremely prevalent in SMI, with 70 to 80% reporting lifetime and half to more than two-thirds reporting current smoking.[22,24-26] Smokers with SMI report using more and higher tar cigarettes and consuming more nicotine compared with other US smokers.[27,28] Smoking may be reinforced in SMI by lessening extrapyramidal effects from decreased blood antipsychotic levels, enhanced cognitive functioning from nicotine, socioeconomic factors and relative lack of other pleasurable pursuits.[26,29]

Diabetes mellitus is estimated ≥ 1.5-2 times higher in SMI than the overall population.[30-35] Multiple factors converge to increase risk of diabetes, and related emerging risks of glucose intolerance/insulin resistance and

the metabolic syndrome in SMI including obesity and physical inactivity. Several antipsychotic medications also are implicated generally in proportion to their impact on weight gain and adiposity.[18,30,36-40]

Hypertension prevalence is 50% higher in persons with SMI than those without.[24] Obesity, physical inactivity, and alcohol use are likely contributing factors to elevated blood pressure.[34,41-44]

Dyslipidemias are also prevalent in SMI; in particular, certain antipsychotics lead to elevated triglycerides and low HDL.[18,34,45-49] With glucose intolerance, hypertension, dyslipidemia and abdominal adiposity all increased in SMI, metabolic syndrome prevalence is correspondingly 1.5 to 2 times higher in SMI as well.[34,50]

Interventions to Reduce CVD Risk in Persons with SMI

In order to accomplish meaningful reductions in CVD risk, targeted interventions will be required for populations with SMI, who urgently need to adopt healthy behaviors and decrease health risk factors.[51] Lifestyle interventions require tailoring for many with SMI, where cognitive deficits as well as other competing demands (e.g., psychiatric symptoms, economic stresses) are highly prevalent. Although few studies have been conducted in PRPs, these facilities are well suited for implementing comprehensive CVD risk reduction interventions as infrastructure facilitates collaboration with staff for patients with SMI.

Interventions to Improve Health Risk Behaviors:

Weight loss and physical activity lifestyle interventions including those addressing multiple health behaviors are effective in the general population, yet these trials systematically exclude persons with SMI who have pressing need. Several studies of behavioral weight loss interventions in SMI have shown success; most focus on diet changes alone and are short-term.[52-58] The ACHIEVE trial was the first long-term trial of a tailored behavioral weight loss intervention resulting in clinically significant weight loss for persons with SMI.[59]

Tobacco cessation interventions are underutilized for persons with SMI despite their modest success with this population. A recent literature review reported more than half of smokers with mental illness are contemplating quitting within 6 months or planning to quit in the next 30 days.[60] The 2009 evidence-based PORT schizophrenia treatment recommendations, co-authored by Dr. Dickerson, recommend smoking cessation treatment, specifically a psychosocial intervention and bupropion +/- nicotine replacement therapy (NRT).[61] These treatments produce evidence of smoking reduction and up to 33% abstinence.[62-70] Since the PORT, varenicline has been found effective for abstinence and well-tolerated in smokers with schizophrenia, bipolar disorder and depression.[71-85] Attendance levels in smoking cessation programs are associated with abstinence outcomes.[65] To-date, interventions addressing all major CVD behavioral risks comprehensively have not been tested in persons with SMI.

Interventions to Improve Health Risk Factors:

In the general population, there is substantial evidence that non-physician interventionists (e.g., care managers) facilitating treatment and coordination with PCPs can improve single and multiple CVD risk factors.[86-92] These interventions are thought beneficial by addressing patient environment and psychosocial factors and decreasing barriers to medication adherence.[91] Other evidence shows interventions focusing directly on medication adherence in primary care are also effective in improving risk factors.[93,94]

Several studies concentrating on multiple CVD risks have targeted SMI. In SMI, a small literature describes interventions in mental health clinics to improve primary care delivery including co-locating primary care (e.g., with nurse practitioner) or a care management model facilitating preventive screening and referral to primary care.[95-98] These programs generally focus on overall health status and have not been specifically designed or staffed (i.e., with skilled interventionist) to effect changes in multiple CVD risk behaviors and factors. More recent evaluations of the behavioral health home incorporating primary care coordination into the specialty mental health care setting and other integrated care programs have shown mixed results with no or minimal effects on CVD risk factors. [97,99-104]

Summary of significance: Persons with SMI are at extremely high risk for CVD morbidity and mortality. Unless effective interventions are developed and tested, this population will continue to lag far behind the nation in CVD goals, and disparities will likely persist if not worsen. If the CVD risk reduction goal that the trial aims to attain could be applied widely to the millions with SMI in the US, tens of thousand of lives could be saved each year.[3,105-107] Psychiatric rehabilitation programs (PRPs) provide an available, yet untapped structure for multifactor CVD risk reduction interventions in SMI.

4. DESIGN SUMMARY

The IDEAL Trial is a community organization-based, two-arm clinical trial that will test the hypothesis that an 18-month comprehensive, practical CVD risk reduction program will be superior to control in improving CV health in persons with SMI. We will enroll a target of 250 persons with SMI who attend 4 psychiatric rehabilitation day programs or adjoining mental health clinics. We will promote a common background of exercise classes and healthy changes to meals served. Participants will be randomized to receive this control condition or the 18-month active IDEAL intervention with a health coach and nurse, who will provide on-site: 1) tailored CVD risk reduction education and counseling (e.g., for smoking cessation, diet); 2) collaboration with physicians to advocate for appropriate management of risk factors (e.g., blood pressure control); and 3) coordination with mental health staff and social supports to encourage and motivate participants to reach individually tailored CV health goals.

5. STUDY POPULATION AND ELIGIBILITY

The study population will consist of adults, ages 18 and older from 4 psychiatric rehabilitation programs and affiliated outpatient mental health clinics. Table 1 lists eligibility criteria. To enhance the generalizability of this trial, we have few exclusion criteria.

**Table 1: Eligibility Criteria**
Inclusion criteria
▪ Age 18 and older
▪ Body mass index at least 25 kg/m^2 OR<br>one of the following CVD risk factors:<br>-Hypertension (SBP>= 140mmHg or DBP>= 90mmHg or on antihypertensive medications;<br>-Diabetes mellitus (fasting blood sugar> 125mg/dl or hemoglobin A1c>6.5 or on a hypoglycemic medication);<br>-Dyslipidemia (LDL >130 mg/dl , HDL<40 or total cholesterol >=200 or on a lipid lowering agent);<br>-Current tobacco smoker
▪ Able and willing to give informed consent
▪ Completion of baseline data collection
▪ Willing to accept randomization
▪ Willing to participate in the intervention
Exclusion criteria
▪ Cardiovascular event (unstable angina, myocardial infarction) within the past 6 months
▪ Serious medical condition which either limits life expectancy or requires active management (e.g., certain cancers)
▪ Condition which interferes with outcome measurement (e.g., dialysis)
▪ Pregnant or planning a pregnancy during study period. Nursing mothers would need approval from physician.
▪ Alcohol or substance use disorder if not sober/abstinent for 30 days
▪ Planning to leave rehabilitation center or clinic within 6 months or move out of geographic area within 18 months
▪ Investigator judgment (e.g., for concerns about participant or staff safety)

Participant eligibility is determined by completion of several screening measures. Screening visits will occur on-site at rehabilitation centers and outpatient clinics. Study instruments are administered in-person by data collectors.

6. RECRUITMENT

Recruitment and retention strategies are facilitated because patients come on-site to the psychiatric rehabilitation programs or nearby to affiliated mental health clinics.

For participant recruitment for the trial at psychiatric rehabilitation programs, we will recruit in several ways including announcements to mental health consumers (i.e., patients); posters; brochures and working with rehabilitation staff. We will make announcements during regular consumer meetings at the sites. We will also post posters and have flyers and brochures available in the rehabilitation center. We will work with rehabilitation staff to identify potential participants by reviewing their list of program attendees with them. Rehabilitation counselors may mention the trial to their consumers. Interested individuals will be directed to contact the study staff by phone or in person at the rehabilitation program. Study staff will be at the rehabilitation sites to discuss the study with any interested consumers. The study will be a behavioral health

organization-wide program offered to all consumers who may be eligible. Recruitment activities will be parallel to/ similar at the outpatient clinics as in the psychiatric rehabilitation programs, except there are no mental health consumer meetings.

Participant retention is a very high priority. We will use strategies successful in the teams' prior studies. In addition to maintaining strong participant rapport, we collect and review contact information throughout and will ask for relatives /friends' contacts who can facilitate communication if needed. We also will have access to information through the PRP and clinic. We will make home visits to collect data on those who move out of the area.

7. DATA COLLECTION AND MEASUREMENTS

Primary outcome variable. The primary outcome, measured from randomization to 18 months, will be the risk of CVD from the global Framingham Risk Score (FRS) expressed as the 10-year probability of a CVD event.[108] In contrast to separate algorithms used to assess risk of specific events[109] (CHD, stroke, PVD and heart failure), the global FRS predicts risk of all CVD events. The global FRS consists of validated, sex-specific multivariable risk functions that include terms for age, total cholesterol, HDL-cholesterol, systolic BP, smoking, diabetes, and treatment for HTN. In clinical practice, such scores have been incorporated into guidelines (e.g., ATPIII) as a means to risk stratify individuals and guide treatment decisions.[110] In research, scores have been used by our group and others to document the overall effects of behavioral and practice-based interventions on CVD risk, considering concomitant changes in all relevant risk factors (systolic BP, total cholesterol, HDL cholesterol, smoking status and diabetes status).[111-114] Six-month FRS is a secondary outcome.

Secondary outcome variables. Weight and BMI; Physical fitness with 6-minute walk test; Healthy diet measured by self-report; Smoking cessation self-report confirmed with expired carbon monoxide level <7; Fasting plasma glucose levels and diabetes mellitus treated to goal (HgBA1c<7.0); Blood pressure levels and hypertension treated to goal (<140/90); Lipid levels (total cholesterol, LDL-C, HDL-C, triglycerides) and dyslipidemia treated to goal (LDL<130, total cholesterol<200).

Risk factor control rates for hypertension, diabetes and dyslipidemia will be calculated with both a single target goal applied to all participants (e.g., blood pressure <140/90, HbBA1c <7.0, and LDL<130) and also with guideline defined subgroup specific thresholds (e.g., BP< 130/80 in diabetes, HbA1c<8.0), and risk-based targets defined by individuals' estimated CVD risk (e.g. dyslipidemia treatment from AHA/ACC ASCVD risk score). The latter is consistent with the evolution of definitions and guidelines that were published after the start of the trial.

We will collect follow-up measures at 6, 12, and 18 months after baseline with in-person contacts at the study sites. (Table 2). The 12-month visit primarily serves as a way to maintain contact with participants.

Measurements will be conducted using standardized operating procedures and quality control methods. Specific study forms will be used to collect data. Table 3 summarizes the data collection schedule.

Detailed measures are described below.

Demographics and Medical History: We will collect demographic and contact information, medical history with a short checklist of conditions, Rose Angina Questionnaire, and assess alcohol or substance use using the ASI-Lite.[115,116]

Mental health diagnoses and medications will be abstracted from PRP (or mental health clinic) charts. Participants and providers will confirm medications.

Physical measures.

Blood pressure will be determined by the OMRON 907 XL, a validated device which records BP using an oscillometric technique.[117] Blood pressure will be obtained by trained, certified data collectors. At each data

collection time point that blood pressure is obtained (3 baseline visits and 1 randomization visit for pre-enrollment time point, 3 visits for follow-up at 6 and 18 months respectively), measurements for blood pressure occur 1week apart, and 3 measurements (each separated by 30 seconds) will be obtained on the right arm of participants after they rest quietly in the seated position for at least 5 minutes.[118]
Weight will be measured to the nearest 0.1 lb by a high quality digital scale with participants wearing light indoor clothes without shoes. Duplicate measurements will be made. Weight will be measured in lbs. for ease of interpretation by participants and converted to kg for calculation of BMI, calculated as the Quetelet index (kg/m^2).
Height to the nearest 0.1 cm will be measured at entry using a wall-mounted stadiometer. The participant stands shoeless on a firm, level surface, with head in the horizontal (Frankfort) plane.
Waist circumference will be measured with an anthropometric tape, in a horizontal plane 1 cm above the navel.
Physical fitness will be measured by the 6-minute walk test. This measure is a valid predictor of VO2 max and has been shown to be responsive to increases in regular moderate physical activity.[119-121]
Fasting blood measures will be collected at Mosaic, centrifuged, aliquoted and sent for processing. Total and HDL cholesterol, triglycerides, glucose, Hemoglobin A1C will be measured directly, and LDL cholesterol will be estimated by Friedwald equation unless direct measurement is needed.[122] Serum CRP and insulin, kidney function (creatinine)) will also be measured.[123-125] We will measure antibodies to viruses and bacteria such as herpes simplex virus and toxoplasma as previous studies have found an association between antibodies to infectious agents and clinical outcomes in serious mental illness. Blood from part of the collected specimens will be frozen at -70° at Johns Hopkins for future investigation of putative risk factors related to cardiovascular disease. Candidate assays include inflammatory markers, leptin and adiponectin. We also will store samples of whole blood at baseline for DNA for later studies. As this study will collect detailed information on medication, blood pressure, BMI, tobacco smoking and traditional laboratory markers of cardiovascular risk pre/post intervention, future analyses of stored serum samples will be able to be interpreted in the valuable context of these other risk factors. Approximately seven teaspoons of blood total will be collected at each study time point with one teaspoon stored for future investigation and ½ teaspoon stored for future DNA testing (DNA storage at baseline only). We plan to store blood samples indefinitely.

Potential participants have specific choices on the consent form to decline or agree to have their blood stored and to DNA testing. The serum samples will be identified with a code that does not include any identifying participant information and will be stored in a secure Johns Hopkins facility. There are currently no plans to re-contact subjects regarding their individual stored serum samples, and we will not release the results of future tests from serum samples to individual subjects. We believe these procedures described above constitute minimal risk for the participants. Study samples may be shared with other researchers partnering with the study team for future research. If we do share any study samples with outside investigators not directly addressing the research questions in this protocol, an amendment/change in research or a new protocol would be submitted to the IRB.

Expired carbon monoxide from the Micro Pro Smokerlyzer (Covita) will be collected and will confirm smoking status (see below).[126] Urinary cotinine will be substituted for CO if the validity of CO is expected to be low (e.g., COPD or difficulty forming an adequate seal on the smokerlyzer) and if the participant is not using nicotine replacement therapy.[127,128]

Urine will also be collected for Na, K, creatinine and protein.

Questionnaires will be used for a variety of purposes including baseline data to describe participants, outcomes to assess intervention effects, and mediating variables to assess potential causal pathways. The study team has experience with these and similar instruments for persons with SMI.[70] Instruments are described here:
Healthy diet. Block Fat, Fruit, Vegetable and Fiber and Sodium Screener Questionnaires provide self-report of daily fruit, vegetable intake, and percent energy from fat.[129] We also will use a questionnaire on sugar sweetened beverages.
Tobacco smoking will be assessed with questions current smoking, 7-day and longer-term abstinence, Fagerstrom Test of Nicotine Dependence (FTND), a 6-item measure of behaviors related to dependence on

nicotine, and self-efficacy questions related to quitting.[130,131] [130,131] Self-report of no cigarettes smoked in the past 7 days confirmed by carbon monoxide <7 will be used to calculate 7-day point prevalence abstinence.[127,132]

Health status will be measured by the Medical Outcomes Study SF-12.[133]
Physical activity will be collected by self-report with the Godin Leisure Time Exercise Questionnaire.
Quality of life will be measured with the Euroqol EQ-5D, a brief 6 item instrument that is valid in persons with schizophrenia and bipolar disorder, and can be used for cost-effectiveness analyses.[134-136]
Medication adherence will be measured with an adapted questionnaire based on the Morisky Medication Adherence Scale for each class of medications for CVD risk factors (e.g., antihypertensives, lipid lowering medications).[137]
Mental health symptoms will be assessed by The Center for Epidemiologic Studies Depression Scale (CES-D) to measure depressive symptoms[138,139] and the Behavior and Symptom Identification Scale (BASIS-24), a brief comprehensive mental status measure for overall mental health status.[140-142]
Social Support will be measured with the Medical Outcomes Study Social Support Questionnaire[143,144]
Other health measures related to CVD risk relevant in SMI population. We will use the Neighborhood Questionnaire to assess participants' home environments for safe places to exercise and places to purchase healthy food, a questionnaire on food and shopping habits, the Questionnaire for Eating and Weight Patterns-Revised to measure Binge Eating, and the Pittsburgh Sleep Quality Index.[145-147] We will assess health literacy with the Health Literacy Skills Instrument.

Participant semi-structured interviews (approximately 15-20 minutes) will be conducted at the end of the 18-month intervention to gain an understanding of participant perceptions of and satisfaction with the heart health program and exercise classes. Interviews will be conducted with a convenience sample of participants across the study locations. Interviews will be audio-recorded and transcribed. Transcripts will not contain names and will be kept in a secure location. Audiotapes will be destroyed after transcription is completed.

**Table 2: Data Collection Schedule**	Baseline	6 mo	12 mo	18 mo
Informed consent	X			
Contact information	X	X	X	X
Demographics	X	X		X
Medical conditions, substance use	X	X		X
Mental heath diagnoses	X			
Medications	X	X	x	X
Physical measures				
Blood pressure	X	X		X
Weight	X	X	x	X
Height	X			
Waist circumference	X	X		X
6 minute fitness walk	X	X		X
Fasting blood measures	X	X		X
Urine measures	x	x		x
Expired Carbon Monoxide	X	X		X
Accclerometery	x	x		x
Questionnaires				
Healthy diet	X	X		X
Tobacco measures	X	X	x	X
Physical activity	X	X		X

Health status	X	X		X
Quality of Life	X	X		X
Medication Adherence	X	X		X
Mental health symptoms	X	X		X
Social Support	X	X		X
Other-Neighborhood, Binge Eating, Sleep, Health literacy	X	X		X
Semi-structured interview				x
Event surveillance		X	X	X

Event surveillance. We will collect data related to health care utilization and medical records related to CVD outcomes of interest. This will include consent documents and medical release forms with appropriate text and a surveillance process that is applied identically to each randomized group.

Food environment: We will analyze a random sample of menus at baseline, 6 and 18 mos. and use ESHA software to assess changes in total calories/nutrients in site meals as a result of dietary consultation.[148]

Staff semi-structured interviews for organizational factors: To better understand the intervention implementation process and perspectives of intervention staff, non-intervention staff and leadership at Mosaic concerning the sustainability and future acceptability for dissemination of IDEAL, we will conduct semi-structured interviews. We will ask about the intervention's perceived effectiveness, ease of or barriers, fit within the organization and financial models for continued support. We will interview before the intervention for baseline information and then at end of the 18-month intervention to assess changes due to the intervention. We will gain an understanding of factors that impact the intervention being acceptable and workable, and prepare for dissemination to other organizations. We expect to conduct interviews with 75 people, or a total of 150 interviews. Questions will include topics of perceived effectiveness of the intervention, ease or barriers for the intervention, fit within the organization and suggestions for future dissemination. The interview will be approximately 30 minutes in length.
Interviews will be audio-recorded and transcribed. Transcripts will not contain names and will be kept in a secure location by Dr. Daumit. Audiotapes will be destroyed after transcription is completed. Individuals will not be identified by name in any publication. Mosaic leadership agrees that staff participation in the interview and any information shared will not affect employment or evaluation at Mosaic.

8. QUALITY ASSURANCE AND QUALITY CONTROL

Quality Assurance pertains to activities that promote collection of high quality data, and Quality Control refers to activities that detect emerging data issues with sufficient time to implement appropriate corrective actions. Our approach to Quality Assurance includes: 1) preparing a manual of operations; 2) implementing a master trainer model to train and certify other staff; 3) train and certify all data collectors; 4) recertify data collectors at least annually; 5) routinely calibrate equipment; 6) maintain logs of certified staff and calibrated equipment. Our approach to Quality Control includes: 1) monitoring counts of completed data collection items; 2) monitoring distribution of trial outcomes, overall, by data collector and site; 3) record lag time in data entry; 4) issue queries on missing data, out of range values or illogical data relations; 4) review types and distribution of data entry errors; and 5) prepare reports for staff, investigators and DSMB on Quality Control. Investigators will review CVD risk factor data to determine study endpoints and adjudicate as needed in a masked fashion.

9. RANDOMIZATION AND BLINDING

Randomization to the IDEAL intervention or control will be stratified by gender and site. Within each stratum, blocked randomization assignments are computer generated by the trial statistician in equal allocation within each block, where the block size is randomly generated to be either a size of 2 or 4. A data table containing these generated assignments is loaded into the trial's REDCap database for web-based delivery to randomize eligible participants after confirmation of eligibility and informed consent. This REDCap randomization assignment table is accessible only to the statistician and the data manager. Prior to obtaining the assignment, the study coordinator will confirm the participant meets all eligibility criteria and all required baseline data have been collected. Then the coordinator logs into REDCap to receive the group assignment for the specific participant and communicates the treatment assignment to the participant. Due to the nature of the intervention, participants and interventionists will be aware of group assignment, however, data collection staff will be kept blinded Due to the nature of the intervention, both participants and interventionists will be aware of the assignment. Until the trial end, investigators, other staff and participants are masked to outcome data, with the exception of trial statistician, study coordinator, data manager and data analyst. We will take considerable efforts to ensure data collection staff will be kept blinded to assignment (e.g., exclude them from all parts of intervention delivery, remind participants not to share group assignment, designate/track unmasked staff).

10. INTERVENTION

Intervention:

Common Background: To establish environments conducive to healthy behaviors and standardized across sites, the study will provide training for group physical activity classes open to all participants, and a dietician to consult to improve the health of meals served. We train PRP staff to deliver group exercise classes using a DVD we provide, and recommend classes are held three times per week. Classes focus on aerobic condition and follow a progression appropriate for sedentary adults, gradually building to 40 minutes of moderate intensity physical activity, and be designed to build exercise confidence.[149] We have used this successfully in SMI.[70,150,151] In order to support active intervention participants' ability to select healthy foods, a study dietician will work with PRP staff to identify healthier menu choices, so that food served is more consistent with AHA Healthy Steps. The dietician will be sensitive to budgetary issues and federal food guidelines. This common background will make available a needed foundation for physical activity and healthy diet choices to occur. However, these offerings are very unlikely to be sufficient alone for significant behavior change.

Randomized groups:

Control condition: The control group will receive this common background.

Active intervention: The active group will receive this common background and the IDEAL intervention.

Figure 1 displays our active IDEAL intervention framework. PRP staff, social supports, mental health providers (e.g., psychiatrists) and PCPs already have relationships with individual patients. For a participant randomized to the IDEAL intervention, the intervention staff (health coach and nurse) work directly with the participant on health risk behaviors and catalyze support from others, serving as a bridge between these individuals and the participant in moving toward ideal health risk behaviors and factors, resulting in improved CV health and quality of life.

Figure 1. IDEAL Intervention Framework for Moving Towards CV Health in Persons with SMI

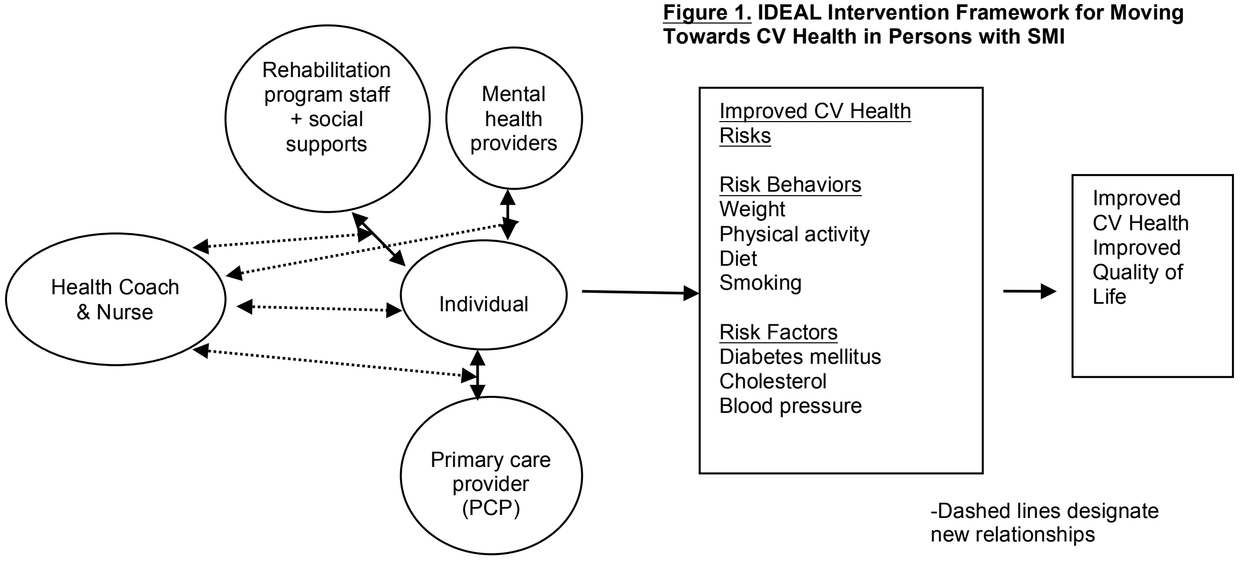

IDEAL Intervention goals include heart healthy behaviors (weight loss, physical activity, diet, smoking cessation) and heart healthy factors (blood sugar, BP, cholesterol) (Table 3). We use AHA goals and modify some (e.g.,10 lb. weight loss instead of BMI<25) to provide intermediate targets achievable with the study sample over 18-months.[8] Strategies combine individual counseling and goal-setting, consulting with the physician to optimize CV risk factor treatment, and working with PRP staff, social supports and mental health providers.

Health coaches coordinate resources and work with participants to develop tailored strategies to achieve the heart healthy goals. Coaches will be physically located on-site at Mosaic. Embedding health coaches at community sites will enable optimal coordination of heart healthy planning with other PRP staff and allow coaches the opportunity, in addition to scheduled sessions, to take advantage of everyday occurrences to reinforce healthy choices and behavior change (e.g., encouraging participant to go to exercise class, checking in and giving positive reinforcement for reporting decreased cigarette use, healthy vending machine choice or improved medication adherence, rewarding a participant for self-monitoring at a weigh-in). Coaches will be skilled facilitators with training in health behavior change and individual-level counseling, with a skill level typical for a community health educator, and will not require a Master's degree.[152] Health coaches will receive training,[153,154] content supervision and will have ongoing, regular interaction with the Intervention Director and the nurse. The health coach is modeled after a position that would be feasible and sustainable in a community mental health setting, with back-up nurse support.

A study nurse will be a Registered Nurse or equivalent with responsibility to support health coaches by serving as an ongoing clinical resource. The nurse regularly will review participants' progress on heart health factors with the coach. He/she will collaborate with PCPs in advocating for participants' CV health. The nurse will be trained in smoking cessation pharmacotherapy principles and will provide NRT and education and monitoring to interested and eligible participants using established guidelines.

The coach and nurse report to an intervention director with motivational interviewing and health behavior change expertise. The intervention director implements quality assurance, and may also provide direct services to participants.

Individual Coaching Sessions (20-30 mins.) will be held at least weekly for up to 6 months, then at least bi-weekly for 12-15 months with the health coach, based on participant need. The initial sessions will focus on building rapport and using motivational interviewing to review current heart healthy risk behaviors and factors, medication adherence, and to identify individual goals. Guided by the FRS score, the participant's abilities, interests and readiness to address each risk factor, risks and targeted behaviors will be addressed either simultaneously or sequentially.[155] (See Table 1). Participants will work with their health coach to identify

behavioral changes topics from a list of recommended sessions (e.g., healthy weight management, becoming smoke free, staying physically active). The coach will use solution-focused therapy to encourage attainment of individual goals and provide reinforcements. The nurse provides educational and counseling sessions related to specific CVD risk factors tailored to participant needs.

Primary Care Provider (PCP) and Psychiatrist Coordination: The nurse will review the participant's baseline labs, physical measurements and medications and then initiate contact with the PCP to share data on baseline CVD risk factors and on any uncontrolled CV health risks and medication adherence. The nurse will accompany the participant on selected visits to the PCP in the community in order to facilitate a collaborative relationship and assist the participant in reaching CV health goals. The nurse identifies modifiable components of the FRS taking into consideration participants' preferences for change and individual abilities. The nurse or coach will follow up with the PCP and psychiatrist as needed to facilitate coordination of behavioral strategies and any pharmacologic treatment and coordinate additional visits as needed. Guidelines for DM, HTN, lipid control and smoking cessation consistent with AHA, JNCVII, ATP-III and ADA and other prevailing guidelines and evidenced-based approaches including those emerging during the conduct of the trial are made available to physicians.[30,41,71,84,85,110,156-164] Intervention staff communicate with participants and providers about tailoring goals as needed (e.g., higher HgbA1c target). SMI patients often have communication barriers, may have medication adherence issues due in part to lack of medication reconciliation across service settings, and often require advocates to make appointments and follow-up on tests. The coach and nurse will facilitate this coordination for improved CV health. For participants who smoke and are interested in pharmacotherapy, the nurse will communicate with PCPs or psychiatrists to advocate for prescription of varenicline or bupropion and/or nicotine replacement therapy (NRT), and may provide NRT.

Staff and Social Supports Coordination: The health coach serves as a change agent, leveraging PRP staff and social supports around the participant's CV health goals. (Figure 1) Coaches when possible attend regular PRP staff case management meetings, which include residential staff for participants in supported housing. Here the coach updates staff on participants' progress with reaching CV health goals. Having the coach on-site enables two-way communication about participant CV health: 1) The coach encourages staff to promote adherence to heart health recommendations (e.g., attending exercise class) and individually identified plans (e.g., setting quit date); 2) PRP staff inform coaches about ongoing issues that could interfere with participant progress towards CV health goals (e.g., change in living situation).The coaches also will encourage social support and may include family members or peers in intervention sessions to support the participant for better control of risk factors (e.g., tobacco smoking). Through all of these pathways, the health coach and nurse coordinate a team-based approach to improve participants' CV health.

Mental Health Provider Coordination: Participants have therapists and psychiatrists they see for mental care and psychotropic medication treatment; for the most part they will be in the adjoining mental health clinics. For uncontrolled risk factors, the health coach or nurse may work with mental health providers to strategize on how to improve CV health (e.g., adherence to diet and medication for diabetes). Psychotropic prescribing practices are not specific intervention targets, however, the psychiatrist may decide to modify medications based on the participant's CVD risks. The health coach and nurse will facilitate communication between the psychiatrist and PCP as needed.

Program materials: Individual visit manuals are developed to guide coaches in conducting sessions. Options tools, cards with pictures of modifiable behaviors based on the participant's risk factors, are used to identify conversation topics. These tools give the participant the autonomy to choose specific behaviors to discuss, while also allowing the coach to provide direction for the session.

Self-monitoring: Participants will be encouraged to self-monitor eating, exercise and smoking behaviors as appropriate to their individually tailored behavioral goals and cognitive abilities. Simple paper trackers are made available for this purpose.

Reinforcements: We plan a points system to reinforce behavior change. Points are awarded for recommended behaviors (e.g., decreased cigarette smoking, group exercise) and can be traded for cheaper items (e.g., pen, key chain) or saved for larger rewards (e.g., CD player). Rewards are used to reinforce healthy habits that forgo immediate gratification (e.g., eating candy now) for larger, delayed gratification (e.g.,weight maintenance).

Quality assurance: We will ensure high-quality intervention delivery through training, case management meetings and fidelity measurement. Coaches receive initial training on CVD risk behaviors and risk factors,

and all intervention staff are trained in behavior change theories, working with persons with SMI, motivational interviewing and implementing the intervention according to the manual of procedures. Follow-up training occurs quarterly. Case management intervention meetings with the study team are used to review each participant's CVD risk factors and current treatment plan and help facilitate behavior change and/or appropriate monitoring or treatment. The meetings ensure that all participants are reviewed regularly, and those with uncontrolled risk factors are prioritized. The intervention director facilitates meetings for the intervention team, and as needed, investigators with expertise in CVD risk reduction strategies participate. To ensure high fidelity, health coach and nurse sessions are either observed in person or audio recorded and reviewed by the intervention director for adherence to the protocol. Fidelity reviews occur at least quarterly.

Process measures: For active intervention participants, we will track intervention session completion, exercise class participation, and interim progress towards goals (e.g., weight, pedometer counts, expired CO) for participant feedback, case management and analysis of potential mediators.

NRT: Nicotine patches and nicotine lozenges are FDA approved and available as over the counter pharmacotherapy for tobacco cessation. For interested participants in the active intervention arm who are smokers and would like to quit or reduce their smoking, we will provide and supervise the use of nicotine patches and nicotine lozenges as nicotine replacement therapy for them. Nicotine patches are dosed every 24 hours; nicotine lozenges (2 and 4mg doses) are used as needed initially every 1-2 hours and then tapering down to every 4-8 hours over several weeks. Nicotine patch initial dosing is based on the level of smoking at baseline, with 21mg generally used for those smoking 1 pack of cigarettes per day; 44mg may be used for those smoking 2 packs per day. Patches and lozenges may be used in combination.

Table 3. Features of IDEAL Active Intervention

CVD Risk Targets	AHA Strategic Goals	IDEAL Trial Goals	Traditional Behavioral Modification Approach	IDEAL Intervention Approach
Healthy Weight	Weight: BMI<25 kg/m^{2*}	Encourage 10 lb. weight loss. Normal weight participants encouraged to maintain weight.	-Lifestyle behavioral counseling<br>-Health education<br>-Self-monitor calories, weight<br>-Participant identifies focus of change<br>-Self-reward	-Patient-centered weight loss counseling<br>-Simplified Healthy Eating tracker<br>-Health coach weighs participant and monitors weight progress<br>-Coach guides participant towards directs high impact behavioral goals<br>-Coach rewards attendance behaviors<br>Encourage and incentivize exercise class , participation and home exercise
Physical Activity	Physical Activity: 150+ min/wk moderate	Physical Activity: 150+ min/wk moderate	-Health education<br>-Role of social support	-Provide PRP with exercise resources<br>-Encourage class attendance, incentivize participation,<br>-Provide pedometer and instructions, provide exercise DVD, reinforce use
Diet	Healthy Diet: 4-5 AHA components	Customized to clinical condition<br>Healthy diet approach similar to DASH emphasizing avoidance of sugar beverages, salty/greasy and processed food, sugar drinks and sweets. Eat smart portions and more fruits/vegetables.	-DASH Diet education<br>-Participant identifies focus of dietary change<br>-Role of social support	-Simplified diet education messages<br>-Wallet size card with dietary messages<br>-Coach recommends specific dietary targets<br>-Coach communicates with residential staff/social supports<br>-Pre-printed shopping lists<br>-"Field Trips" to model decision making<br>-Consult with PRP staff to provide healthier meals on-site
Smoking	Stop smoking	Stop smoking<br><br>Increased readiness to quit smoking	-Provide general information about risks of smoking and benefits of quitting<br>-5As: Ask, Advise, Assess, Assist, Arrange<br>-4D's (Delay, Deep Breathe, Drink water, Do something to take your mind of smoking)<br>-Provide information on nicotine replacement therapy and other pharmacotherapy	-Counseling tailored to readiness to quit<br>-Motivational interviewing and 4D's<br>-Coach communicates with PRP staff, residential program counselor and/or home social support about goals and strategies for smoking cessation<br>-Facilitate access/provide nicotine replacement therapy (NRT) and work with physician and individual to incorporate reducing/quitting into treatment plan, --Communicate with PCP and psychiatrist to advocate for pharmacotherapy for smoking cessation including varenicline, bupropion and NRT<br>-Contingency management with CO testing
Control Blood Sugar	Fasting glucose: <100	HbA1c<7% for diabetes	-Heath education<br>-Self monitoring: carbohydrate intake and blood sugar<br>-Problem solving to increase medication and diet adherence	-Teach/reinforce self-management skills<br>-Assist with glucose monitoring<br>-Simplified dietary messages: (e.g., "No sugar drinks")<br>-Solution-focused therapy for medication adherence.<br>- Coordinate with PCP regarding HbA1c targets and any suggested medication changes -Communicate with PRP staff, residential counselor and/or home social support about diet and medication<br>-Encourage and incentivize exercise class participation and home exercize
Control BP	Blood Pressure <120/80 or treated to goal	Control blood pressure, SBP <140 mmHg, DBP<90mmHg	-Heath education<br>-Self monitoring: sodium intake and blood pressure<br>-Problem solving to increase medication and diet adherence	-Simplified messages ("Avoid packaged/canned foods")<br>-Solution-focused therapy for medication adherence -Coordinate with PCP about BP therapeutic goals and any medication adjustments<br>-Coach communicates with PRP staff, residential counselor and/or home social support about diet and medication<br>-Encourage and incentivize exercise class participation and home exercise

Lower Cholesterol	Total Cholesterol <200 or treated to goal	Lower cholesterol, total cholesterol <200mg/dl, LDL<130 mg/dl*	-Heath education<br>-Self monitoring: calorie and fat intake<br>-Problem solving to increase medication and diet adherence	-Simplified dietary messages (e.g., "Avoid junk food")<br>-Solution-focused therapy for medication adherence<br>-Coordinate with PCP regarding cholesterol therapeutic goals and medication<br>-Coach communicates with PRP staff, residential counselor and/or home social support about diet and medication<br>-Encourage and incentivize exercise class participation and home exercise

11. DATA MANAGEMENT

We will store data in REDCap.[165] Built-in range and logic checks will prompt data checks and confirmation in real-time during data collection. The analyst will routinely conduct thorough checking and cleaning, examining distributions and data patterns and evaluation to detect inconsistencies. Outliers (e.g., extreme weight changes) will be identified using Rosner's extreme Studentized deviate (ESD).[166] Every effort will be made to determine correctness of outliers in a timely manner. Confirmed outliers will be flagged and set aside in the principal secondary analyses. Those removed will be treated as all other missing data; sensitivity analyses will be conducted to assess influence of outliers on results. The data manager will create detailed variable documentation and the data analyst will conduct analysis per protocol under direction of the study statistician.

12. DATA ANALYSIS

Our target is to enroll two-hundred and fifty participants recruited from 4 sites in 1 health organization, randomized in equal allocation to active intervention (Group A) and control (Group B), stratified by site. Formal assessments are at baseline, 6, and 18 months. Primary analyses will be conducted under the intention to treat principle.[167]

Core analytical model: Let Y_{ijk} denote the log-transformed FRS for the ith participant at the jth visit (j = 0, 1, or 2), corresponding to the baseline, 6, and 18-month visits), in the kth treatment group (k = 0 or 1 corresponding to intervention groups A and B). X_{ik} is a vector of pre-randomization covariates (which will include study site indicators). The longitudinal regression model for the primary analysis is:$E[Y_{ijk}] = X_{ik}\eta + \mu + \alpha_j + \beta_k + \gamma_{jk} + e_{ijk}$ with the constraints that each of $(\alpha_j, \beta_k, \gamma_{jk})$ equals 0 when an index is as its lowest value. The e_{ijk} are independent unless the (i, k) subscripts are the same. For participant (i,k) the e_{ijk} have an unstructured 3×3 covariance matrix. The η measure the influence of baseline covariates, the α_j give visit effects within Group A, β_k baseline treatment effects (which under effective randomization should be close to 0) and the γ_{jk} are the treatment by visit interactions, "the treatment effects." The primary testing contrast is to compare baseline to 18-month changes in FRS (the primary outcome) between intervention groups, i.e. testing the hypothesis that $\gamma_{21} = 0$. Data at 6 months, though not the primary outcome, will help determine whether the changes over 18 months are linear. The primary analysis will follow the intention to treat principle and will not adjust for post randomization variables. This core model is also applicable to other secondary continuous outcomes such as weight, blood pressure and lipids, and other CVD risk scores, in their original scale or log-transformed as appropriate. Modeling will be implemented via SAS PROC Mixed (or the Stata or R-equivalents). Site effects will be included to account for possible within-site clustering.

Missing and incomplete data: Prevention is far superior to statistical treatments; every effort will be made to collect outcome data on all randomized. The underlying missing data process determines the biasing effects of missing data and structures valid analytic strategies.[168] The primary analysis above treats all missing primary outcomes as resulting from a MAR process, through which the probability of missing can depend on all observed information (Observed FRS, covariates) but does not depend on any FRS scores that are not recorded. If the probability of a potential observation being missing depends on what has, but not on what hasn't been observed, termed missing at random (MAR), then estimates based on an appropriate analytic model (both the mean and error structure) for the observed data will not be biased. A valid model for the observed data allows the missing data process to be ignored. If the missing is informative, meaning that the probability of an observation being missing depends on the value of that observation, then bias cannot be avoided by modeling the observed data alone, and the impact of such bias would be evaluated using sensitivity analysis. Data set aside (censored) due to protocol defined high-leverage events such as pregnancy or a serious medical event (e.g., liver failure) that may strongly influence components of the FRS will be treated as missing at random.

Secondary Analyses

Analysis of secondary outcomes*:* We will replicate the foregoing analyses for measured outcomes (BP [raw scale], lipid levels and HgbA1c levels [log scale]. For dichotomous outcomes (e.g., HTN control, lipid control, diabetes control, smoking cessation) GEE based logistic regression modeling will be conducted using SAS

Proc Genmod (or the Stata or R-equivalents) with a logistic link and a within-individual, unstructured correlation structure. We will use data for all participants to compare changes in outcomes over time between intervention arms. For certain dichotomous outcomes we will include only “at risk” participants (e.g., smoking cessation in smokers). Medication use for CVD risk factors and psychotropics will be assessed and compared between arms and adherence examined using conventional methods.[169-171]

Sensitivity Analyses: We will conduct sensitivity analyses with multiple imputation with respect to departures from the MAR assumption. The model will be derived from our primary analytical model under MAR, maintaining the covariance structure and modifying the imputation mean according to informative missing data scenarios. Second, to evaluate potential impact of out of window FRS scores on the robustness of findings from the primary analysis, we will set aside all the out of window measurements and repeat the analyses following the core analytic modeling approach described previously. When the number of out of window FRS scores is high, we will modify the core analytic model with more flexible mean (e.g. use the actual follow-up time as a continuous variable rather than a categorical variable with prespecified follow-up time window) and covariance (e.g., heterogeneous TOEP or heterogeneous AR) structure to accommodate the more variable intervals for follow-up times between 2 outcome assessments. We will produce model-based estimate on mean outcome changes at 6 and 18 months between groups and utilize robust inferences. Third, we will conduct analyses restricting the study sample to those in age range to 30-79, which corresponds to the suggested age range of the FRS.

Exploratory Analyses: We will perform several types of exploratory analyses related to other outcomes of interest, and potential moderators or mediators. We will explore the effects of the IDEAL intervention using other potential outcomes of interest. For example, we will investigate the AHA/ACC ASCVD risk score which became available during the trial. We will systematically evaluate the efficacy of the IDEAL intervention in study subgroups defined by baseline (pre-randomization) variables. We are especially interested in baseline variables related to psychiatric conditions (psychiatric diagnosis, symptoms, substance use and psychotropic medications). We will also explore the efficacy of the intervention in subgroups by baseline cvd risk, race,gender and medications for CVD risk factors. These analyses will be performed by including the interaction (product) terms in the analysis models for study outcomes. The trial is not powered for these exploratory analyses, and we will be careful in interpreting any findings.

In addition to these and for analyses that preserve the intention to treat principle, we will also perform informative exploratory analyses based on subgroups defined post-randomization to characterize aspects of the intervention that may be related to improved outcomes in the active group. Such analyses will use post-randomization variables that are potential mediators - interventionist visit and exercise class participation, medication modifications, medication adherence and self-monitoring activities.[172] For example, for patients with BP controlled at baseline with medications, medications may be lowered if lifestyle changes occur. As a result, gauging intervention effects based on measured BP or HTN control alone may underestimate such effects as effects could partially be reflected though reduced use of medications. These analyses are more prone to biases so that protection from confounding afforded by randomization is not likely applicable. Propensity score stratifications will be used to manage potential confounding in these analyses. Nevertheless, these findings will be interpreted with caution and will be considered exploratory.

Cost Analysis. The primary analysis will be conducted from the perspective of an adopting organization or payer (e.g., Medicaid). The main goal is to estimate the incremental implementation costs of the active intervention relative to the control. Resource measurement will focus on sampling and tracking intervention staff time. We will sample at specified intervals throughout the intervention at each site for one week at a time. Tracking specific tasks will allow us to eliminate time spent strictly on research and perform sensitivity analyses with different potential levels of efficiency (e.g., amount of active client involvement versus administrative time). We will also have data on self-reported utilization of office visits and hospitalizations and medication data that can be used to calculate cost per participant and total costs. With estimates of total costs for the active group and control, we will be able to estimate incremental costs for the active intervention and the incremental costs per incremental improvement in 1°and 2°outcomes. To extend the time horizon forward, we will use the global FRS to project the proportion in each group expected to have a CV event within 10 years and the present

value of costs and QALYs associated with events. This will allow us to perform a societal perspective cost-utility analysis.[173] For all analyses, we will conduct univariate sensitivity analyses by varying assumptions and assess the uncertainty of our estimates by bootstrapping for 10000 replications, drawing samples with replacement where samples have the same number of observations as the main study.[174]

Qualitative Analysis: Interviews will be transcribed and coded. NVivo will be used to enter and group discrete passages from data into themes. We will use the constant comparative method[175] to categorize transcript statements that demonstrate common attributes, then combine categories into broader, recurrent themes using a hybrid approach where we will start with larger categories and then add nodes and subnodes as new concepts emerge until saturation of ideas is reached. Findings will be compiled to inform ongoing implementation and future sustainability and dissemination.

Power analyses: The trial has sufficient resources to enroll a target of 250 participants. With this sample size, our objective was to determine the minimum detectable difference (MDD) in relative risk reduction in the global Framingham Risk Score (FRS). Our assumptions are as follows:

- Two-sided type I α error=0.05; type II β error=0.20; 90% follow-up.
- Baseline absolute CVD risk with global FRS =11% from ACHIEVE Trial (n=291, Table 1);
- Standard deviation of change in global FRS=3% from ongoing ACHIEVE Trial (from Dr. Daumit's pilot weight loss intervention in SMI, similar SD of 2.9%);
- Global FRS change in controls=5% relative risk reduction. Controls could experience some reduction in CVD risk because of secular trends and offerings of physical activity classes on-site. However, aging increases CVD risk and might counterbalance these effects. We conservatively assume a 5% relative reduction in the control group, even though their risk might not change or might increase over time.

With these assumptions, our MDD is a 10.7% reduction in relative risk in global FRS.

Table 4: Calculation of Relative Risk Reduction to 10 year Framingham Risk Score from Baseline to Follow-up

	Baseline FRS Risk	Follow-up FRS Risk	Relative Reduction, within-group		Relative Reduction, between-group	Minimum Detectable Difference (MDD)
	10yr Probability of CVD Event		Relative Risk	Percent Risk Reduction	Relative Risk Ratio (RRR)	MDD=1-RRR
Active	11%	9.33%	9.33%/11%=0.848	1 - 0.848 = 0.152 (15.2%)	0.848/0.95=0.893	1 - 0.893 = 0.107 (10.7%)
Control	11%	10.45%	10.45%/11%=0.95	1 - 0.95 =0.05 (5%)		

Table 4 demonstrates the relation between baseline and 18-month follow-up and absolute and relative changes in global FRS with the example of a percent risk reduction in the control group of 5% and an MDD of 10.7%. The reduction in relative risk ratio of 10.7% (MDD= 1 – RRR) is very similar in magnitude to the direct difference in relative risks [95% - 84.8% = 10.2%] at 18 months. If risk is unchanged or rises in the controls, our MDD will be lower.

Table 6. Reported CVD risk factor changes and estimated relative reduction in global FRS

Table 5. Minimal Detectable Differences for Relative Framingham Risk Score Reduction by follow-up rates and % risk reduction in controls

	MDD, assuming a Relative Reduction in Control Group of:		
Follow-up rate	**0%**	**2.5%**	**5%**
100%	9.7%	10.0%	10.1%
95%	9.9%	10.2%	10.4%
90%	10.3%	10.5%	**10.7%**
85%	10.5%	10.8%	11.1%
80%	10.9%	11.1%	11.4%

Risk Factor	Reported range of change in risk factor	Corresponding Δ in Global FRS♯
SBP	Lifestyle interventions: 3.7-8mmHg [176-178]	5-15%
	Care management: 6-11mmHg [93, 179, 180]	6-19%
Total cholesterol	Lifestyle intervention: 19mg/dl [178]	10%
	Care management: 12.9-15.5 mg/dl [91, 181]	7-7.5%
HDL	Lifestyle interventions: 2-2.4mg/dl[177, 178]	4-5%
Smoking cessation	Psychosocial /buproprion +/-NRT* 12-33%[62-68]	1 -3.3%±
Fasting glucose/HgbA1C	Lifestyle interventions: HgbA1c 0.5-0.99[177, 182]	
	Care management: fasting glucose, 7.7-20mg/dl, HbgA1c 1.1[91, 180, 183]	
*in SMI. ± After weighting by the anticipated prevalence of smoking. ♯ To estimate global FRS Δ, we used median risk factor levels in ACHIEVE. Then, for each risk factor separately, we applied literature-reported changes in that risk factor to the global FRS equation.		

Table 5 displays MDD for ranges of follow-up rates and relative risk reduction in the control group. The MDD of 10.7% is very achievable. IDEAL will combine lifestyle/behavioral modification with improved clinical management of CVD risk factors. In studies of lifestyle interventions, reported relative reductions in CV risk scores range from 9-16%,[111,113,178] and for care management interventions, [91,180] from 12%-23%. Table 6 displays ranges of changes in individual CV risk factors and corresponding estimated changes in global FRS from interventions in the literature with components similar to IDEAL. Even with the understanding that intervention components will not likely be fully additive, and that except for smoking cessation, these estimates are from studies in the general population and not SMI, Table 6 provides support for many scenarios where the MDD in FRS of 10.7% would be attainable. We have precision for estimating clinically significant intervention effects in secondary outcomes. The 95%confidence interval (CI) for an effect estimate is +/-1.96* sqrt(2/n)*σ, where n is the sample size per group and σ the common SD for change in the measured outcome of interest for both groups. Assuming 90% follow-up, the 95% CI for the intervention effect of difference in change between groups would be: glucose 7.9 mg/dl; SBP 3.5mmHg, weight 4.0 lbs; total cholesterol 9.0 mg/dl; HDL 2.9mg/dl; SF-12 Physical Functioning 7.9. The SDs for change are derived from the ACHIEVE Trial. For smoking cessation, we assume 75 smokers per group with 1.3% quit rate in the controls (i.e., 1 of 75). We will have 80% power to detect a quit rate of 14% or higher in the active group using a 2-sided alpha of 0.05.

13. DATA SECURITY

Each participant will be assigned a study ID number that will be used, instead of name or other identifying information, on all study data collection materials. The link between identifying information and the study ID will be kept in a separate database with password access available only to the data analyst and principal investigators. Paper records will be kept in locked file cabinets. Data will be published only in aggregate, with no identifying characteristics of individuals published or presented. All study staff annually sign a confidentiality statement attesting to their understanding of, and willingness to abide by, written policies on research ethics and confidentiality. For data entry and management, we will use REDCap, a web-based application for building and managing data entry and databases. [165] REDCap data is housed on secure servers at the Johns Hopkins Biostatistics Center under firewall protection with offsite access through VPN. The database will contain embedded checks for internal consistency and data completeness. Missing and questionable data will be queried in source documents and corrected, and double data entry will be performed for primary outcome data. Database access is password protected and restricted to authorized personnel only (e.g., data collectors cannot see intervention data), and REDCap provides audit trails for tracking data manipulation and user activity. Data will be exported from REDCap to SAS for analysis. Analytic files will be stored on secure Johns Hopkins network servers and accessed on encrypted and password-protected computers.

14. SAFETY

Safety Monitoring

Participant safety will be closely monitored. Protection of research participants begins with the eligibility criteria, which are designed to exclude individuals with a serious medical condition that would preclude their ability to participate in a cardiovascular risk reduction program. During the study, clinical care will be provided by the participants' usual specialty mental health providers and the primary care physician, not by the study.

If a participant develops a medical or psychiatric issue, the safety of continuing or resuming the intervention will be ascertained by the study clinician in collaboration with the participant's regular providers. Surveillance for serious adverse events and other relevant clinical events will occur by questionnaire at regularly scheduled intervals. We may be come aware of medical problems including abnormal blood pressure or laboratory tests. Results of routine clinical labs and physical measures obtained as a part of data collection will be provided to participants and physicians at baseline and after study completion. Measures meeting criteria for alert values will be communicated more quickly.

Potential Risks

The study should not involve any major risk to screenees and trial participants. Recommended treatments, both lifestyle and pharmacologic, are based on prevailing national guidelines. However, there are some risks. Sources of risk include medication-related adverse events, such as hypoglycemic events, side effects from medications for CVD risk factors prescribed by the patient's primary care physician and side effects from nicotine withdrawal or over-the-counter nicotine replacement therapy. For example, improved health behaviors from the intervention (e.g., physical activity, weight loss) may lead to hypoglycemia or hypotension in those already taking medications and necessitate adjusting diabetes or blood pressure medications. In addition, physical activity is associated with a small risk of cardiovascular complications (less than 1 per 187,500 person-hours of exercise).[184] Physical activity can also increase the risk of musculoskeletal discomfort or injury. Blood drawing may cause some discomfort and/or bruising at the site of the venipuncture. The methods that participants may use to restrict calories (e.g. extreme calorie restriction or unbalanced dietary pattern) may lead to inadequate energy intake or lower than recommended intake of essential nutrients. In addition,

participants run the risk of a breach in confidentiality. These risks should be rare. Plans to minimize these risks are described below.

Adequacy of Protection Against Risks

Informed consent. Procedures for recruitment, consent, intervention, data collection and analysis will be reviewed and approved by the Johns Hopkins Institutional Review Boards and the DSMB.

We will use a two-stage consent procedure: one consent form will be signed upon entry that covers screening and baseline data collection for the study. Another will be signed at the time of randomization. The consent forms will detail the purpose of the study, the requirements for participation, and the potential benefits and risks. In consenting individuals to participate in the study, the following procedures will be used: 1) Procedures involved in the study will be fully explained to participants by study staff trained in informed consent procedures for persons with serious mental illness. 2) Participants will complete a brief test of comprehension of study procedures to demonstrate that they understand the risks and benefits of study participation and clearly consent, before consent is accepted. Any wrong answers are corrected and additional information given to clarify, until a person can easily answer all items successfully or they indicate they do not wish to continue. 3) Those agreeing to participate will sign the consent form.

Protection against risk: General

The safety approach will be implemented similar to the investigators' other trials (e.g., POWER Trial). We will have a medical safety officer who will be in charge of developing the safety protocol for the trial, and he will promote and monitor safety throughout the study. As in our previous studies, the safety officer does not provide direct medical care and is not involved in the intervention. We will use standardized materials including a manual of procedures and forms. NHLBI has considered materials that we developed in the POWER Trial as models which are now used in other NHLBI-supported lifestyle intervention trials.

Hypoglycemia, hypotension or other potential consequences of improved health behaviors or changes in medication: These will be minimized by having the intervention team communicate closely with the primary care physician and rehabilitation program staff about medication changes, exercise participation and coordinate assistance for participants in blood sugar monitoring. For example, if an active intervention group participant is taking a sulfonylurea and has significantly increased exercise frequency, the intervention team will communicate with the PCP and raise the suggestion of a glucose-lowering agent that would have lower risk of hypoglycemia, such as metformin. The interventionist will also ensure appropriate follow-up with the PCP after any medication changes.

Physical activity: The small risk of physical activity will be minimized by emphasizing moderate (as opposed to vigorous) activity, and by following American College of Sports Medicine guidelines regarding need for medical examination prior to beginning an exercise program. Participants who wish to progress to vigorous activity will be advised to obtain approval from their primary care physician. Risk of injury is further minimized by instruction on proper exercise technique, the importance of warm-up and cool-down exercises, and proper stretching techniques.

Blood drawing: All phlebotomy is performed by an experienced phlebotomist. Participants will be given information on how to contact Dr. Daumit if complications occur.

Extreme weight loss methods. The interventionist and mental health program staff will be aware of the possibility that intervention subjects could use extreme methods to lose weight. Participants will be reminded regularly of the importance of safe weight loss. Those who have a sudden, marked weight reduction will be interviewed to determine if extreme measures have been taken. The intervention staff will be trained to detect evidence of extreme measures and will be given strategies for responding.

Nicotine replacement therapy and smoking cessation. We will educate participants about symptoms of nicotine withdrawal and teach coping strategies. NRT including in combination (patches and lozenges) is considered safe and risks are far less than those associated with tobacco use, even in smokers with known cardiovascular disease and in those who continue smoking while using NRT.[185-192] Certain plasma levels of medications including olanzapine, clozapine and fluvoxamine may be higher after smoking cessation. We will discuss use of NRT with participants' primary care physicians and psychiatrists and monitor symptoms carefully at each

weekly to bi-weekly intervention visit so that risks are minimized. We will communicate with providers regarding smoking cessation so that psychotropic medication dosage adjustment may be made if needed.

Breach of confidentiality: All participant information will be considered confidential. This confidentiality will be assured through several mechanisms. For the study, each participant will be assigned an anonymous study ID, which will be used on all study forms. In addition, all study forms and paper records that contain participant information will be kept in secured, locked areas when not in use. Such materials, when in use, will be kept away from public scrutiny. Forms that need to be discarded will be destroyed. Moreover, access to all participant data and information will be restricted to authorized personnel. In the case of computerized study data, access will be password protected. When the study database is ready for analysis, it will not contain actual identities of participants. During active data collection, hard copies of data collection forms will be stored in locked areas with access only by authorized personnel. Finally, neither the mental health programs nor participants will be identified by name in any publications. Data will not be presented in such a way that the identity of individual participants can be inferred.

Similarly, for staff semi-structured interviews, all information will be considered confidential. This confidentiality will be assured through several mechanisms. Transcripts will not contain names and will be kept in a secure location by Dr. Daumit. Audiotapes will be destroyed after transcription is completed. Individuals will not be identified by name in any publication. Mosaic leadership agrees that staff participation in the interview and any information shared will not affect staff employment or evaluation.

Participant reimbursement: At baseline and 6 months, for completing data collection activities, participants will receive 15 dollars. At 18 months, for completing data collection activities, participants will receive 25 dollars. This increase in payment as participants' time in the study progresses is to recognize their commitment to the study. Staff will receive a 25 dollar gift card for completing a semi-structured interview.

Importance of knowledge to be gained: Persons with serious mental illness have high burdens of each major cardiovascular risk behavior, yet persons with SMI have been systematically excluded from interventions to decrease cardiovascular risk. If successful, this proposed cardiovascular risk reduction intervention could be disseminated widely. The minimal health risks to participants are offset by the potential benefits to participants and to the greater population with chronic mental illness.

Potential benefits to the study participants who receive the active intervention include improved health behaviors and improved cardiovascular risk factor profiles. The physical activity classes are available for active and control participants. Potential benefits to others include the possibility that the research will lead to the dissemination of effective interventions to decrease CVD risk in persons with serious mental illness.

There is no specific benefit to staff for participating in interviews. Information learned from their perspectives potentially will help disseminate health interventions in mental health organizations in the future.

Data and safety monitoring plan: Protection of research participants begins with the eligibility criteria, which are designed to exclude individuals with a serious medical condition that would preclude their ability to participate in a cardiovascular risk reduction program. During the study, clinical care will be provided by the participants' usual specialty mental health providers and the primary care physician, not by the study. Participants will be made aware of this delineation of responsibility. However, recognizing the opportunity for early detection of clinical problems and the small risk of study-related morbidity, we will perform periodic safety assessments. We will inquire about cardiovascular and musculoskeletal symptoms at 6-month intervals. Safety monitoring will consist of a symptom questionnaire and the Rose Angina questionnaire. Any positive responses to these questions will be reviewed by the safety officer; clinically significant results will lead to referral to the primary care physician. Information that comes to the attention of study personnel informally (e.g., through data collection or intervention activities at the center) may also lead to referral. Symptoms that will lead to referral include (but are not limited to) those that suggest cardiovascular disease (e.g., exertional chest pain, dyspnea, presyncope or syncope), uncontrolled hypertension, uncontrolled diabetes or

complications of physical activity. Participants are also queried at these same timepoints about possible adverse events (defined below). Positive responses trigger an adverse event (AE) record, which is reviewed and classified as either gastrointestinal, cardiovascular, musculoskeletal, or other in nature. This information is then reported to the DSMB. Similar information reported by participants at other times (e.g., during intervention classes) is duly noted and followed up with as needed to assure participant safety. The following constitute adverse events (AEs): heart attack, stroke, transient ischemic attack, heart failure, coronary angioplasty or bypass surgery, angina pectoris, broken bone, torn ligament, and any other serious injury to the bone or muscle. Evidence of the occurrence of these events is based on participant self-report that a health care professional has diagnosed the condition. We will attempt to verify the diagnosis through contact with the primary care physician. Though not considered AEs for this study, we also track and report the incidence of hyperlipidemia, gallbladder disease, diabetes, cancer and hospitalization. All other outcomes that may be construed as being an adverse consequence of study participation, such as an injury while performing a study measurement, are documented, reviewed, and followed up on as needed by a study clinician.

A Data and Safety Monitoring Board will be established and will meet at least annually during the study. The DSMB members will be research scientists not otherwise connected to the study whose expertise includes the disciplines and skills needed to initially review the protocol and then monitor intervention progress, quality of the data and safety of the participants. Members will include experts in CVD prevention, mental health and clinical trials. Prevailing NIH and IRB policies will dictate the specific roles of this committee, which will review the protocol before the start of fieldwork. Adverse events will be reviewed initially by the safety officer and Dr. Daumit and then reported locally to the IRB, the DSMB and the NIH project office according to prevailing policies of these review bodies. This plan should ensure participant safety.

15. REFERENCES

1. Health, United States, 2009: With special features on Medical Technology. Hyattsville, MD: National Center for Health Statistics; 2010.
2. Brown S. Excess mortality of schizophrenia. A meta-analysis. Br J Psychiatry 1997;171:502-8.
3. Daumit GL, Anthony CB, Ford DE, et al. Pattern of mortality in a sample of Maryland residents with severe mental illness. Psychiatry Res 2010;176:242-5.
4. Osby U, Correia N, Brandt L, Ekbom A, Sparen P. Mortality and causes of death in schizophrenia in Stockholm county, Sweden. Schizophr Res 2000;45:21-8.
5. Saha S, Chant D, McGrath J. A systematic review of mortality in schizophrenia: is the differential mortality gap worsening over time? Arch Gen Psychiatry 2007;64:1123-31.
6. Mueser KT, McGurk SR. Schizophrenia. Lancet 2004;363:2063-72.
7. Primm AB, Vasquez MJ, Mays RA, et al. The role of public health in addressing racial and ethnic disparities in mental health and mental illness. Prev Chronic Dis 2010;7:A20.
8. Lloyd-Jones DM, Hong Y, Labarthe D, et al. Defining and setting national goals for cardiovascular health promotion and disease reduction: the American Heart Association's strategic Impact Goal through 2020 and beyond. Circulation 2010;121:586-613.
9. Allison DB, Fontaine KR, Heo M, et al. The distribution of body mass index among individuals with and without schizophrenia. J Clin Psychiatry 1999;60:215-20.
10. Coodin S. Body mass index in persons with schizophrenia. Can J Psychiatry 2001;46:549-55.
11. Daumit GL, Clark JM, Steinwachs DM, Graham CM, Lehman A, Ford DE. Prevalence and correlates of obesity in a community sample of individuals with severe and persistent mental illness. J Nerv Ment Dis 2003;191:799-805.
12. McElroy SL. Correlates of Overweight and Obesit in 644 Patients with Bipolar Disorder. J Clin Psychiatry 2002;63:207-13.
13. Depression Guideline Panel: Depression in Primary Care, vol. 2. Rockville, MD: AHCPR; 1993. Report No.: 93-0551.
14. Allison DB, Mentore JL, Heo M, et al. Antipsychotic-induced weight gain: a comprehensive research synthesis. Am J Psychiatry 1999;156:1686-96.
15. Baptista T, Kin NM, Beaulieu S, de Baptista EA. Obesity and related metabolic abnormalities during antipsychotic drug administration: mechanisms, management and research perspectives. Pharmacopsychiatry 2002;35:205-19.
16. Macritchie KAN, Geddes JR, Scott J, Haslam DRS, Goodwin GM. Valproic acid, valproate and divalproex in the maintenance treatment of bipolar disorder (Cochrane Review). The Cochrane Library2002.
17. Sachs GS, Guille C. Weight gain associated with use of psychotropic medications. J Clin Psychiatry 1999;60 Suppl 21:16-9.
18. Lieberman JA, Stroup TS, McEvoy JP, et al. Effectiveness of antipsychotic drugs in patients with chronic schizophrenia. N Engl J Med 2005;353:1209-23.
19. Daumit GL, Goldberg RW, Anthony C, et al. Physical activity patterns in adults with severe mental illness. J Nerv Ment Dis 2005;193:641-6.
20. Jerome GJ, Young DR, Dalcin A, et al. Physical activity levels of persons with mental illness attending psychiatric rehabilitation programs. Schizophr Res 2009;108:252-7.
21. Amani R. Is dietary pattern of schizophrenia patients different from healthy subjects? BMC Psychiatry 2007;7:15.
22. McCreadie RG, Scottish Schizophrenia Lifestyle G. Diet, smoking and cardiovascular risk in people with schizophrenia: descriptive study. Br J Psychiatry 2003;183:534-9.
23. Strassnig M, Brar JS, Ganguli R. Nutritional assessment of patients with schizophrenia: a preliminary study. Schizophr Bull 2003;29:393-7.

24. Goff DC, Sullivan LM, McEvoy JP, et al. A comparison of ten-year cardiac risk estimates in schizophrenia patients from the CATIE study and matched controls. Schizophr Res 2005;80:45-53.
25. Holmberg SK, Kane C. Health and self-care practices of persons with schizophrenia. Psychiatr Serv 1999;50:827-9.
26. Ziedonis D, Hitsman B, Beckham JC, et al. Tobacco use and cessation in psychiatric disorders: National Institute of Mental Health report. Nicotine Tob Res 2008;10:1691-715.
27. Tidey JW, Rohsenow DJ, Kaplan GB, Swift RM. Cigarette smoking topography in smokers with schizophrenia and matched non-psychiatric controls. Drug and alcohol dependence 2005;80:259-65.
28. Williams JM, Ziedonis DM, Abanyie F, Steinberg ML, Foulds J, Benowitz NL. Increased nicotine and cotinine levels in smokers with schizophrenia and schizoaffective disorder is not a metabolic effect. Schizophrenia research 2005;79:323-35.
29. Goff DC, Henderson DC, Amico E. Cigarette smoking in schizophrenia: relationship to psychopathology and medication side effects. Am J Psychiatry 1992;149:1189-94.
30. Consensus Development Conference on Antipsychotic Drugs and Obesity and Diabetes. Diabetes Care 2004;27:596-58.
31. Bresee LC, Majumdar SR, Patten SB, Johnson JA. Prevalence of cardiovascular risk factors and disease in people with schizophrenia: A population-based study. Schizophr Res 2010.
32. Bushe C, Leonard B. Association between atypical antipsychotic agents and type 2 diabetes: review of prospective clinical data. Br J Psychiatry Suppl 2004;47:S87-93.
33. Dixon L, Weiden P, Delahanty J, et al. Prevalence and correlates of diabetes in national schizophrenia samples. Schizophr Bull 2000;26:903-12.
34. McEvoy JP, Meyer JM, Goff DC, et al. Prevalence of the metabolic syndrome in patients with schizophrenia: baseline results from the Clinical Antipsychotic Trials of Intervention Effectiveness (CATIE) schizophrenia trial and comparison with national estimates from NHANES III. Schizophr Res 2005;80:19-32.
35. Sokal J, Messias E, Dickerson F, et al. Co-morbidity of medical illnesses among adults with serious mental illness who are receiving community psychiatric services. Journal of Nervous and Mental Disease 2004;192:421-7.
36. Casey DE. Metabolic issues and cardiovascular disease in patients with psychiatric disorders. Am J Med 2005;118 Suppl 2:15S-22S.
37. Dwyer DS, Pinkofsky HB, Liu Y, Bradley RJ. Antipsychotic drugs affect glucose uptake and the expression of glucose transporters in PC12 cells. Prog Neuropsychopharmacol Biol Psychiatry 1999;23:69-80.
38. Haupt DW, Newcomer JW. Hyperglycemia and antipsychotic medications. J Clin Psychiatry 2001;62 Suppl 27:15-26; discussion 40-1.
39. Newcomer JW. Comparing the safety and efficacy of atypical antipsychotics in psychiatric patients with comorbid medical illnesses. J Clin Psychiatry 2009;70 Suppl 3:30-6.
40. Newcomer JW, Haupt DW, Fucetola R, et al. Abnormalities in Glucose Regulation during antipsychotic treatment of Schizophrenia. Arch Gen Psychiatry 2002;59:337-45.
41. Chobanian AV, Bakris GL, Black HR, et al. The Seventh Report of the Joint National Committee on Prevention, Detection, Evaluation, and Treatment of High Blood Pressure: the JNC 7 report. JAMA 2003;289:2560-72.
42. Koskinen J, Lohonen J, Koponen H, Isohanni M, Miettunen J. Prevalence of alcohol use disorders in schizophrenia--a systematic review and meta-analysis. Acta Psychiatr Scand 2009;120:85-96.
43. Newcomer JW. Medical risk in patients with bipolar disorder and schizophrenia. J Clin Psychiatry 2006;67 Suppl 9:25-30; discussion 6-42.
44. Dixon L, Postrado L, Delahanty J, Fischer PJ, Lehman A. The association of medical comorbidity in schizophrenia with poor physical and mental health. J Nerv Ment Dis 1999;187:496-502.
45. Casey DE. Dyslipidemia and atypical antipsychotic drugs. J Clin Psychiatry 2004;65 Suppl 18:27-35.
46. Daumit GL, Goff DC, Meyer JM, et al. Antipsychotic effects on estimated 10-year coronary heart disease risk in the CATIE schizophrenia study. Schizophr Res 2008;105:175-87.

47. L'Italien GJ, Casey DE, Kan HJ, Carson WH, Marcus RN. Comparison of metabolic syndrome incidence among schizophrenia patients treated with aripiprazole versus olanzapine or placebo. J Clin Psychiatry 2007;68:1510-6.
48. Meyer JM, Davis VG, McEvoy JP, et al. Impact of antipsychotic treatment on nonfasting triglycerides in the CATIE Schizophrenia Trial phase 1. Schizophr Res 2008;103:104-9.
49. Newcomer JW. Second-generation (atypical) antipsychotics and metabolic effects: a comprehensive literature review. CNS Drugs 2005;19 Suppl 1:1-93.
50. Sharpe JK, Hills AP. Anthropometry and adiposity in a group of people with chronic mental illness. Aust N Z J Psychiatry 1998;32:77-81.
51. Newcomer JW, Hennekens CH. Severe mental illness and risk of cardiovascular disease. JAMA 2007;298:1794-6.
52. Brar JS, Ganguli R, Pandina G, Turkoz I, Berry S, Mahmoud R. Effects of behavioral therapy on weight loss in overweight and obese patients with schizophrenia or schizoaffective disorder. Journal of Clinical Psychiatry 2005;66:205-12.
53. Evans S, Newton R, Higgins S. Nutritional intervention to prevent weight gain in patients commenced on olanzapine: a randomized controlled trial. Aust N Z J Psychiatry 2005;39:479-86.
54. Faulkner G, Soundy AA, Lloyd K. Schizophrenia and weight management: a systematic review of interventions to control weight. Acta Psychiatr Scand 2003;108:324-32.
55. Kwon JS, Choi JS, Bahk WM, et al. Weight management program for treatment-emergent weight gain in olanzapine-treated patients with schizophrenia or schizoaffective disorder: A 12-week randomized controlled clinical trial. J Clin Psychiatry 2006;67:547-53.
56. Littrell KH, Hilligoss NM, Kirshner CD, Petty RG, Johnson CG. The effects of an educational intervention on antipsychotic-induced weight gain. J Nurs Scholarsh 2003;35:237-41.
57. McKibbin CL, Patterson TL, Norman G, et al. A lifestyle intervention for older schizophrenia patients with diabetes mellitus: a randomized controlled trial. Schizophr Res 2006;86:36-44.
58. Weber M, Wyne K. A cognitive/behavioral group intervention for weight loss in patients treated with atypical antipsychotics. Schizophr Res 2006;83:95-101.
59. Daumit GL, Dickerson FB, Wang NY, et al. A behavioral weight-loss intervention in persons with serious mental illness. N Engl J Med 2013;368:1594-602.
60. Siru R, Hulse GK, Tait RJ. Assessing motivation to quit smoking in people with mental illness: a review. Addiction 2009;104:719-33.
61. Kreyenbuhl J, Buchanan RW, Dickerson FB, Dixon LB, Schizophrenia Patient Outcomes Research T. The Schizophrenia Patient Outcomes Research Team (PORT): updated treatment recommendations 2009. Schizophr Bull 2010;36:94-103.
62. Evins AE, Cather C, Deckersbach T, et al. A double-blind placebo-controlled trial of bupropion sustained-release for smoking cessation in schizophrenia. J Clin Psychopharmacol 2005;25:218-25.
63. Evins AE, Cather C, Rigotti NA, et al. Two-year follow-up of a smoking cessation trial in patients with schizophrenia: increased rates of smoking cessation and reduction. J Clin Psychiatry 2004;65:307-11; quiz 452-3.
64. Evins AE, Mays VK, Rigotti NA, Tisdale T, Cather C, Goff DC. A pilot trial of bupropion added to cognitive behavioral therapy for smoking cessation in schizophrenia. Nicotine Tob Res 2001;3:397-403.
65. Baker A, Richmond R, Haile M, et al. A randomized controlled trial of a smoking cessation intervention among people with a psychotic disorder. Am J Psychiatry 2006;163:1934-42.
66. Ziedonis DM, George TP. Schizophrenia and nicotine use: report of a pilot smoking cessation program and review of neurobiological and clinical issues. Schizophrenia bulletin 1997;23:247-54.
67. Weiner E, Ball MP, Summerfelt A, Gold J, Buchanan RW. Effects of sustained-release bupropion and supportive group therapy on cigarette consumption in patients with schizophrenia. Am J Psychiatry 2001;158:635-7.

68. George TP, Vessicchio JC, Termine A, et al. A placebo controlled trial of bupropion for smoking cessation in schizophrenia. Biol Psychiatry 2002;52:53-61.
69. George TP, Ziedonis DM, Feingold A, et al. Nicotine transdermal patch and atypical antipsychotic medications for smoking cessation in schizophrenia. Am J Psychiatry 2000;157:1835-42.
70. Casagrande SS, Jerome GJ, Dalcin AT, et al. Randomized trial of achieving healthy lifestyles in psychiatric rehabilitation: the ACHIEVE trial. BMC Psychiatry 2010;10:108.
71. Tsoi DT, Porwal M, Webster AC. Interventions for smoking cessation and reduction in individuals with schizophrenia. Cochrane Database Syst Rev 2013;2:CD007253.
72. Dutra SJ, Stoeckel LE, Carlini SV, Pizzagalli DA, Evins AE. Varenicline as a smoking cessation aid in schizophrenia: effects on smoking behavior and reward sensitivity. Psychopharmacology (Berl) 2011.
73. Williams JM, Anthenelli RM, Morris CD, et al. A randomized, double-blind, placebo-controlled study evaluating the safety and efficacy of varenicline for smoking cessation in patients with schizophrenia or schizoaffective disorder. J Clin Psychiatry 2012;73:654-60.
74. Weiner E, Buchholz A, Coffay A, et al. Varenicline for smoking cessation in people with schizophrenia: a double blind randomized pilot study. Schizophr Res 2011;129:94-5.
75. Pachas GN, Cather C, Pratt SA, et al. Varenicline for Smoking Cessation in Schizophrenia: Safety and Effectiveness in a 12-Week, Open-Label Trial. J Dual Diagn 2012;8:117-25.
76. Evins AE. Reassessing the Safety of Varenicline. Am J Psychiatry 2013;170.
77. Gibbons RD, Mann JJ. Varenicline, Smoking Cessation, and Neuropsychiatric Adverse Events. Am J Psychiatry 2013.
78. Wu BS, Weinberger AH, Mancuso E, et al. A Preliminary Feasibility Study of Varenicline for Smoking Cessation in Bipolar Disorder. J Dual Diagn 2012;8:131-2.
79. George TP, Vessicchio JC, Termine A, et al. A placebo controlled trial of bupropion for smoking cessation in schizophrenia. Biological psychiatry 2002;52:53-61.
80. Heffner JL, Anthenelli RM, Delbello MP, Stahl L, Strakowski SM. Mood management and nicotine patch for smoking cessation in adults with bipolar disorder. Nicotine Tob Res 2012;15:1805-6.
81. Weinberger AH, Vessicchio JC, Sacco KA, Creeden CL, Chengappa KN, George TP. A preliminary study of sustained-release bupropion for smoking cessation in bipolar disorder. J Clin Psychopharmacol 2008;28:584-7.
82. Evins AE. Extended Duration Pharmacotherapy for Sustained Tobacco Abstinence in People with Serious Mental Illness In Symposium: $\alpha_4\beta_2$-Nicotinic Acetylcholine Receptors In Schizophrenia: Implications for Comorbid Tobacco Smoking and Therapeutics. Society for Research on Nicotine and Tobacco 2013; Boston, MA.
83. Chengappa KN. Varenicline for Initiating Smoking Cessation in Persons with Bipolar Disorder A Random-Assignment, Placebo-Controlled, Parallel Group Clinical Trial (PowerPoint). American Psychiatric Assoication 166th Annual Meeting San Franciso, CA.(5/18/2013).
84. Evins AE, Cather C, Pratt SA, et al. Maintenance treatment with varenicline for smoking cessation in patients with schizophrenia and bipolar disorder: a randomized clinical trial. JAMA 2014;311:145-54.
85. Anthenelli RM, Benowitz NL, West R, et al. Neuropsychiatric safety and efficacy of varenicline, bupropion, and nicotine patch in smokers with and without psychiatric disorders (EAGLES): a double-blind, randomised, placebo-controlled clinical trial. The Lancet 2016;387:2507-20.
86. Renders C, Valk G, Griffin S, Wagner E, Thm van Eijk J, Assendelft W. Interventions to improve the management of diabetes mellitus in primary care, outpatient and community settings. Cochrane database of systematic reviews 2000:CD001481.
87. Glynn L, Murphy A, Smith S, Schroeder K, Fahey T. Interventions used to improve control of blood pressure in patients with hypertension. Cochrane Database of Systematic Reviews 2010;3:CD005182.
88. Schedlbauer A, Davies P, Fahey T. Interventions to improve adherence to lipid lowering medication. Cochrane Database Syst Rev 2010:CD004371.

89. Haskell WL, Berra K, Arias E, et al. Multifactor cardiovascular disease risk reduction in medically underserved, high-risk patients. The American journal of cardiology 2006;98:1472-9.
90. Haskell WL, Alderman EL, Fair JM, et al. Effects of intensive multiple risk factor reduction on coronary atherosclerosis and clinical cardiac events in men and women with coronary artery disease. The Stanford Coronary Risk Intervention Project (SCRIP). Circulation 1994;89:975-90.
91. Becker DM, Yanek LR, Johnson WR, Jr., et al. Impact of a community-based multiple risk factor intervention on cardiovascular risk in black families with a history of premature coronary disease. Circulation 2005;111:1298-304.
92. Katon WJ, Lin EH, Von Korff M, et al. Collaborative care for patients with depression and chronic illnesses. N Engl J Med 2010;363:2611-20.
93. Ogedegbe G, Chaplin W, Schoenthaler A, et al. A practice-based trial of motivational interviewing and adherence in hypertensive African Americans. Am J Hypertens 2008;21:1137-43.
94. Schedlbauer A, Davies P, Fahey T. Interventions to improve adherence to lipid lowering medication. Cochrane Database Syst Rev 2010:CD004371.
95. Druss BG, Rohrbaugh RM, Levinson CM, Rosenheck RA. Integrated medical care for patients with serious psychiatric illness: a randomized trial. Arch Gen Psychiatry 2001;58:861-8.
96. Druss BG, von Esenwein SA. Improving general medical care for persons with mental and addictive disorders: systematic review. Gen Hosp Psychiatry 2006;28:145-53.
97. Druss BG, von Esenwein SA, Compton MT, Rask KJ, Zhao L, Parker RM. A randomized trial of medical care management for community mental health settings: the Primary Care Access, Referral, and Evaluation (PCARE) study. Am J Psychiatry 2010;167:151-9.
98. Umbricht-Schneiter A, Ginn DH, Pabst KM, Bigelow GE. Providing medical care to methadone clinic patients: referral vs on-site care. Am J Public Health 1994;84:207-10.
99. Breslau J, Leckman-Westin E, Han B, et al. Impact of a mental health based primary care program on emergency department visits and inpatient stays. General Hospital Psychiatry 2018;52:8-13.
100. Breslau J, Leckman-Westin E, Yu H, et al. Impact of a Mental Health Based Primary Care Program on Quality of Physical Health Care. Administration and Policy in Mental Health and Mental Health Services Research 2018;45:276-85.
101. Druss BG, von Esenwein SA, Glick GE, et al. Randomized Trial of an Integrated Behavioral Health Home: The Health Outcomes Management and Evaluation (HOME) Study. Am J Psychiatry 2017;174:246-55.
102. Gilmer TP, Henwood BF, Goode M, Sarkin AJ, Innes-Gomberg D. Implementation of Integrated Health Homes and Health Outcomes for Persons With Serious Mental Illness in Los Angeles County. Psychiatr Serv 2016;67:1062-7.
103. Krupski A, West, II, Scharf DM, et al. Integrating Primary Care Into Community Mental Health Centers: Impact on Utilization and Costs of Health Care. Psychiatr Serv 2016;67:1233-9.
104. Tepper MC, Cohen AM, Progovac AM, et al. Mind the Gap: Developing an Integrated Behavioral Health Home to Address Health Disparities in Serious Mental Illness. Psychiatr Serv 2017;68:1217-24.
105. Heidenreich PA, Trogdon JG, Khavjou OA, et al. Forecasting the future of cardiovascular disease in the United States: a policy statement from the American Heart Association. Circulation 2011;123:933-44.
106. Heart Disease and Stroke Statistics — 2010 Update2010.
107. Incidence and Prevalence: 2006 Chart Book on Cardiovascular and Lung Diseases. Bethesda, MD2006.
108. D'Agostino RB, Ramachandran SV, Pencina MJ, et al. General cardiovascular risk profile for use in primary care: The Framingham Heart Study. Circulation 2008;17:743-53.
109. Wilson PW, D'Agostino RB, Levy D, Belanger AM, Silbershatz H, Kannel WB. Prediction of coronary heart disease using risk factor categories. Circulation 1998;97:1837-47.
110. Third Report of the National Cholesterol Education Program (NCEP) Expert Panel on Detection, Evaluation, and Treatment of High Blood Cholesterol in Adults (Adult Treatment Panel III) final report. Circulation 2002;106:3143-421.

111. Randomised controlled trial evaluating cardiovascular screening and intervention in general practice: principal results of British family heart study. Family Heart Study Group. BMJ 1994;308:313-20.
112. Appel LJ, Sacks FM, Carey VJ, et al. Effects of protein, monounsaturated fat, and carbohydrate intake on blood pressure and serum lipids: results of the OmniHeart randomized trial. Jama 2005;294:2455-64.
113. Maruthur NM, Wang NY, Appel LJ. Lifestyle interventions reduce coronary heart disease risk: results from the PREMIER Trial. Circulation 2009;119:2026-31.
114. Willaing I, Ladelund S, Jorgensen T, Simonsen T, Nielsen LM. Nutritional counselling in primary health care: a randomized comparison of an intervention by general practitioner or dietician. European journal of cardiovascular prevention and rehabilitation : official journal of the European Society of Cardiology, Working Groups on Epidemiology & Prevention and Cardiac Rehabilitation and Exercise Physiology 2004;11:513-20.
115. Rose G, McCartney P, Reid DD. Self-administration of a questionnaire on chest pain and intermittent claudication. Br J Prev Soc Med 1977;31:42-8.
116. Cacciola JS, Alterman AI, McLellan AT, Lin YT, Lynch KG. Initial evidence for the reliability and validity of a "Lite" version of the Addiction Severity Index. Drug Alcohol Depend 2007;87:297-302.
117. White WB, Anwar YA. Evaluation of the overall efficacy of the Omron office digital blood pressure HEM-907 monitor in adults. Blood Press Monit 2001;6:107-10.
118. Vollmer WM, Appel LJ, Svetkey LP, et al. Comparing office-based and ambulatory blood pressure monitoring in clinical trials. J Hum Hypertens 2005;19:77-82.
119. Laboratories ATSCoPSfCPF. ATS statement: guidelines for the six-minute walk test. Am J Respir Crit Care Med 2002;166:111-7.
120. Grant S, Corbett K, Amjad AM, Wilson J, Aitchison T. A comparison of methods of predicting maximum oxygen uptake. Br J Sports Med 1995;29:147-52.
121. Johnson DJ, Oliver RA, Terry JW. Regression equation for prediction of performance in the twelve minute run walk test. J Sports Med Phys Fitness 1979;19:165-70.
122. Friedewald WT, Levy RI, Fredrickson DS. Estimation of the concentration of low-density lipoprotein cholesterol in plasma, without use of the preparative ultracentrifuge. Clin Chem 1972;18:499-502.
123. Bingham SA, Gill C, Welch A, et al. Validation of dietary assessment methods in the UK arm of EPIC using weighed records, and 24-hour urinary nitrogen and potassium and serum vitamin C and carotenoids as biomarkers. Int J Epidemiol 1997;26 Suppl 1:S137-51.
124. Martini MC, Campbell DR, Gross MD, Grandits GA, Potter JD, Slavin JL. Plasma carotenoids as biomarkers of vegetable intake: the University of Minnesota Cancer Prevention Research Unit Feeding Studies. Cancer Epidemiol Biomarkers Prev 1995;4:491-6.
125. van Kappel AL, Steghens JP, Zeleniuch-Jacquotte A, Chajes V, Toniolo P, Riboli E. Serum carotenoids as biomarkers of fruit and vegetable consumption in the New York Women's Health Study. Public Health Nutr 2001;4:829-35.
126. Jarvis MJ, Russell MA, Saloojee Y. Expired air carbon monoxide: a simple breath test of tobacco smoke intake. Br Med J 1980;281:484-5.
127. Verification SSoB. Biochemical verification of tobacco use and cessation. Nicotine Tob Res 2002;4:149-59.
128. Acosta M, Buchhalter A, Breland A, Hamilton D, Eissenberg T. Urine cotinine as an index of smoking status in smokers during 96-hr abstinence: comparison between gas chromatography/mass spectrometry and immunoassay test strips. Nicotine Tob Res 2004;6:615-20.
129. Block G, Gillespie C, Rosenbaum EH, Jenson C. A rapid food screener to assess fat and fruit and vegetable intake. Am J Prev Med 2000;18:284-8.
130. Heatherton TF, Kozlowski LT, Frecker RC, Fagerstrom KO. The Fagerstrom Test for Nicotine Dependence: a revision of the Fagerstrom Tolerance Questionnaire. Br J Addict 1991;86:1119-27.

131. Hughes JR, Keely JP, Niaura RS, Ossip-Klein DJ, Richmond RL, Swan GE. Measures of abstinence in clinical trials: issues and recommendations. Nicotine & tobacco research : official journal of the Society for Research on Nicotine and Tobacco 2003;5:13-25.
132. Karelitz JL, Michael VC, Perkins KA. Analysis of Agreement between Expired-Air Carbon Monoxide Monitors. J Smok Cessat 2017;12:105-12.
133. Salyers MP, Bosworth HB, Swanson JW, Lamb-Pagone J, Osher FC. Reliability and validity of the SF-12 health survey among people with severe mental illness. Medical Care 2000;38:1141-50.
134. Bobes J, Garcia-Portilla P, Saiz PA, Bascaran T, Bousono M. Quality of life measures in schizophrenia. Eur Psychiatry 2005;20 Suppl 3:S313-7.
135. Fryback DG, Dunham NC, Palta M, et al. US norms for six generic health-related quality-of-life indexes from the National Health Measurement study. Med Care 2007;45:1162-70.
136. Hayhurst H, Palmer S, Abbott R, Johnson T, Scott J. Measuring health-related quality of life in bipolar disorder: relationship of the EuroQol (EQ-5D) to condition-specific measures. Qual Life Res 2006;15:1271-80.
137. Morisky DE, Ang A, Krousel-Wood M, Ward HJ. Predictive validity of a medication adherence measure in an outpatient setting. J Clin Hypertens (Greenwich) 2008;10:348-54.
138. Radloff L. The CES-D Scale: A self-report depression scale for research in the general population. Applied Psyhcological Measurement 1977;1:385-401.
139. Weissman MM, Sholomskas D, Pottenger M, Prusoff BA, Locke BZ. Assessing depressive symptoms in five psychiatric populations: a validation study. Am J Epidemiol 1977;106:203-14.
140. Eisen SV, Normand SL, Belanger AJ, Spiro A, 3rd, Esch D. The Revised Behavior and Symptom Identification Scale (BASIS-R): reliability and validity. Med Care 2004;42:1230-41.
141. Eisen SV, Ranganathan G, Seal P, Spiro A, 3rd. Measuring clinically meaningful change following mental health treatment. J Behav Health Serv Res 2007;34:272-89.
142. Niv N, Cohen AN, Mintz J, Ventura J, Young AS. The validity of using patient self-report to assess psychotic symptoms in schizophrenia. Schizophr Res 2007;90:245-50.
143. Sallis JF, Pinski RB, Grossman RM, Patterson TL, Nader PR. The development of self efficacy scales for health-related diet and exercise behaviors. Health Education Research 1988;3:283-92.
144. Sherbourne CD, Stewart AL. The MOS social support survey. Soc Sci Med 1991;32:705-14.
145. Celio AA, Wilfley DE, Crow SJ, Mitchell J, Walsh BT. A comparison of the binge eating scale, questionnaire for eating and weight patterns-revised, and eating disorder examination questionnaire with instructions with the eating disorder examination in the assessment of binge eating disorder and its symptoms. Int J Eat Disord 2004;36:434-44.
146. Mujahid MS, Diez Roux AV, Morenoff JD, Raghunathan T. Assessing the measurement properties of neighborhood scales: from psychometrics to ecometrics. American journal of epidemiology 2007;165:858-67.
147. Buysse D, Reynolds C, Monk T, Berman S, Kupfer D. The Pittsburgh Sleep Quality Index (PSQI): A new instrument for psychiatric research and practice. Psychiatry Research 1989;28:193-213.
148. Casagrande SS, Dalcin A, McCarron P, et al. A Nutritional Intervention to Reduce the Calorie Content of Meals Served at Psychiatric Rehabilitation Programs. Community Ment Health J 2011.
149. American Heart Association and National Heart L, and Blood Institute. Exercise and Your Heart: A guide to physical activity AHA; 2001.
150. Daumit GL, Dalcin AT, Jerome GJ, et al. A behavioral weight loss intervention for persons with serious mental illness in psychiatric rehabilitation centers. International Journal of Obesity 2010:1–10.
151. Daumit G, Appel L, Leatherman E, et al. Randomized Trial of Peer-Supported Physical Activity for Persons with Severe Mental Illness in Community Psychiatry. Journal of General Internal Medicine 2010;25:378-9.
152. Gary TL, Batts-Turner M, Yeh HC, et al. The effects of a nurse case manager and a community health worker team on diabetic control, emergency department visits, and hospitalizations among urban African Americans with type 2 diabetes mellitus: a randomized controlled trial. Arch Intern Med 2009;169:1788-94.

153. Campbell MK, Resnicow K, Carr C, Wang T, Williams A. Process evaluation of an effective church-based diet intervention: Body & Soul. Health Educ Behav 2007;34:864-80.
154. Hill-Briggs F, Batts-Turner M, Gary TL, et al. Training community health workers as diabetes educators for urban African Americans: value added using participatory methods. Prog Community Health Partnersh 2007;1:185-94.
155. Hyman DJ, Pavlik VN, Taylor WC, Goodrick GK, Moye L. Simultaneous vs sequential counseling for multiple behavior change. Arch Intern Med 2007;167:1152-8.
156. Standards of medical care in diabetes--2011. Diabetes care 2011;34 Suppl 1:S11-61.
157. Appel LJ, Brands MW, Daniels SR, Karanja N, Elmer PJ, Sacks FM. Dietary approaches to prevent and treat hypertension: a scientific statement from the American Heart Association. Hypertension 2006;47:296-308.
158. Evins AE. Reassessing the safety of varenicline. Am J Psychiatry 2013;170:1385-7.
159. Stone NJ, Robinson JG, Lichtenstein AH, et al. 2013 ACC/AHA guideline on the treatment of blood cholesterol to reduce atherosclerotic cardiovascular risk in adults: a report of the American College of Cardiology/American Heart Association Task Force on Practice Guidelines. Circulation 2014;129:S1-45.
160. Treating tobacco use and dependence: 2008 update U.S. Public Health Service Clinical Practice Guideline executive summary. Respir Care 2008;53:1217-22.
161. James PA, Oparil S, Carter BL, et al. 2014 evidence-based guideline for the management of high blood pressure in adults: report from the panel members appointed to the Eighth Joint National Committee (JNC 8). Jama 2014;311:507-20.
162. Ebbert JO, Hughes JR, West RJ, et al. Effect of Varenicline on Smoking Cessation Through Smoking Reduction: A Randomized Clinical Trial. Jama 2015;313:687-94.
163. American Diabetes Association. Standards of medical care in diabetes—2014. Diabetes Care 2014;37:S14-S80.
164. Buchanan RW, Kreyenbuhl J, Kelly DL, et al. The 2009 schizophrenia PORT psychopharmacological treatment recommendations and summary statements. Schizophr Bull 2010;36:71-93.
165. Harris PA, Taylor R, Thielke R, Payne J, Gonzalez N, Conde JG. Research electronic data capture (REDCap)- A metadata-and workflow process for providing translational research informatics support. J Biomed Infor 2009;42:377-81.
166. Rosner B. Fundamentals of biostatistics: Volume 1 6th ed ed. Belmont, CA: Duxbury Press; 2006.
167. Piantadosi S. Clinical Trials: A Methodologic Perspective. Second ed. Baltimore, MD: Wiley Interscience; 2005.
168. Little R, Rubin D. Statistical Analysis with Missing Data, Second Edition: Wiley and Sons; 2002.
169. Redmon JB, Bertoni AG, Connelly S, et al. Effect of the look AHEAD study intervention on medication use and related cost to treat cardiovascular disease risk factors in individuals with type 2 diabetes. Diabetes Care 2010;33:1153-8.
170. Muntner P, Joyce C, Holt E, et al. Defining the minimal detectable change in scores on the eight-item Morisky Medication Adherence Scale. Ann Pharmacother 2011;45:569-75.
171. Krousel-Wood M, Joyce C, Holt E, et al. Predictors of decline in medication adherence: results from the cohort study of medication adherence among older adults. Hypertension 2011;58:804-10.
172. Kraemer HC, Wilson GT, Fairburn CG, Agras WS. Mediators and moderators of treatment effects in randomized clinical trials. Arch Gen Psychiatry 2002;59:877-83.
173. Gold M, Siegel J, Russell L, Weinstein M. Cost-Effectiveness in Health and Medicine. New York: Oxford University Press; 1996.
174. Briggs AH, O'Brien BH, Blackhouse G. Thinking outside the box: recent advances in the analysis and presentation of uncertainty in cost-effectiveness studies. Annu Rev Public Health 2002;23:377-401.
175. Glaser BG. The Constant Comparative Method of Qualitative-Analysis. Social Problems 1965;12:436-45.

176. Appel L, Champagne C, Harsha D, et al. Effects of Comprehensive Lifestyle Modification on Blood Pressure Control: Main results of the PREMIER Clinical Trial. Writing Group of the PREMIER Collaborative Research Group. JAMA 2003;289:2083-93.
177. Pi-Sunyer X, Blackburn G, Brancati FL, et al. Reduction in weight and cardiovascular disease risk factors in individuals with type 2 diabetes: one-year results of the look AHEAD trial. Diabetes care 2007;30:1374-83.
178. Bove AA, Santamore WP, Homko C, et al. Reducing cardiovascular disease risk in medically underserved urban and rural communities. Am Heart J 2011;161:351-9.
179. Hiss RG, Armbruster BA, Gillard ML, McClure LA. Nurse care manager collaboration with community-based physicians providing diabetes care: a randomized controlled trial. Diabetes Educ 2007;33:493-502.
180. Ma J, Berra K, Haskell WL, et al. Case management to reduce risk of cardiovascular disease in a county health care system. Arch Intern Med 2009;169:1988-95.
181. Barr Taylor C, Miller NH, Reilly KR, et al. Evaluation of a nurse-care management system to improve outcomes in patients with complicated diabetes. Diabetes Care 2003;26:1058-63.
182. Spencer MS, Rosland AM, Kieffer EC, et al. Effectiveness of a Community Health Worker Intervention Among African American and Latino Adults With Type 2 Diabetes: A Randomized Controlled Trial. Am J Public Health 2011.
183. Welch G, Garb J, Zagarins S, Lendel I, Gabbay RA. Nurse diabetes case management interventions and blood glucose control: results of a meta-analysis. Diabetes Res Clin Pract 2010;88:1-6.
184. ACSM. ACSM's guidelines for exercise testing and prescription/American College of Sports Medicine. Philadelphia: Lippincott Williams & Wilkins; 2000.
185. Benowitz NL, Gourlay SG. Cardiovascular toxicity of nicotine: implications for nicotine replacement therapy. J Am Coll Cardiol 1997;29:1422-31.
186. Dale LC, Hurt RD, Offord KP, Lawson GM, Croghan IT, Schroeder DR. High-dose nicotine patch therapy. Percentage of replacement and smoking cessation. JAMA 1995;274:1353-8.
187. Heishman SJ, Balfour DJ, Benowitz NL, Hatsukami DK, Lindstrom JM, Ockene JK. Society for Research on Nicotine and Tobacco. Addiction 1997;92:615-33.
188. Jorenby DE, Smith SS, Fiore MC, et al. Varying nicotine patch dose and type of smoking cessation counseling. JAMA 1995;274:1347-52.
189. Zevin S, Jacob P, 3rd, Benowitz NL. Dose-related cardiovascular and endocrine effects of transdermal nicotine. Clin Pharmacol Ther 1998;64:87-95.
190. Gourlay SG, Benowitz NL. The benefits of stopping smoking and the role of nicotine replacement therapy in older patients. Drugs Aging 1996;9:8-23.
191. Rose JE, Behm FM, Westman EC, Kukovich P. Precessation treatment with nicotine skin patch facilitates smoking cessation. Nicotine Tob Res 2006;8:89-101.
192. Rose JE, Herskovic JE, Behm FM, Westman EC. Precessation treatment with nicotine patch significantly increases abstinence rates relative to conventional treatment. Nicotine Tob Res 2009;11:1067-75.

Date: __8/28/13
Principal Investigator: _Gail Daumit
Application Number: _NA 00072197__________

JHM IRB - eForm A – Protocol

- **Use the section headings to write the JHM IRB eForm A, inserting the appropriate material in each. If a section is not applicable, leave heading in and insert N/A.**
- **When submitting JHM IRB eForm A (new or revised), enter the date submitted to the field at the top of JHM IRB eForm A.**

****

1. Abstract

a. Provide no more than a one page research abstract briefly stating the problem, the research hypothesis, and the importance of the research.

Persons with serious mental illness (SMI), such as schizophrenia and bipolar disorder, die on average 25 years earlier than persons without SMI. Much premature death in SMI is attributable to CVD with CVD-related mortality rates twice those of the overall population. Accordingly, this vulnerable group, comprising at least 15 million Americans, has a high burden of modifiable CVD risk factors including smoking, obesity, hypertension, diabetes, and dyslipidemia. The American Heart Association (AHA) recently set ambitious strategic Impact Goals to improve the cardiovascular health of all Americans and reduce deaths from CVD by 20 percent by 2020. Substantial efforts are underway to meet these goals in the general population. Still, interventions need to be adapted for persons with SMI who often have substantial barriers related to cognitive impairment and psychiatric symptoms. Hence, without special efforts to promote CV health and control risk factors in vulnerable populations like the SMI, it is unlikely that CV health will improve in SMI patients, and disparities in CV health will not only persist, but likely worsen. In view of their extraordinary burden of CVD risk factors, the overarching objective of this protocol is to develop and test a scalable intervention that could be widely adopted to reduce risk in this vulnerable population. Our multidisciplinary research team has expertise designing and rigorously testing interventions to decrease CVD risk in the general population and in persons with SMI.

The IDEAL Trial is a community organization-based, two-arm clinical trial that will test the hypothesis that an 18-month comprehensive, practical CVD risk reduction program will be superior to control in improving CV health in persons with SMI. We will partner with Mosaic Community Services, Inc., a large mental health care organization, and enroll 250 persons with SMI who attend 4 psychiatric rehabilitation day programs (PRPs) or adjoining mental health clinics. Rehabilitation programs provide opportune settings to implement CVD risk reduction programs because patients attend several days a week and environmental components can take place on-site. We will provide a common background of exercise classes and healthy changes to meals served. Participants will then be randomized to receive this control condition or the 18-month active IDEAL intervention with a Heart Health Interventionist, who will provide on-site: 1) individual CVD risk reduction behavioral counseling (e.g., for smoking cessation, diet); 2) coordination with primary care providers to ensure appropriate management of risk factors (e.g., blood pressure control); and 3) collaboration with mental health staff and social supports to encourage and motivate participants to reach goals. The primary outcome will be the change in estimated CVD risk from the global Framingham Risk Score; secondary outcomes will include AHA metrics for CV health. Accomplishing meaningful reductions in CVD throughout the population will require targeted interventions focusing on vulnerable, high-risk groups such as those with SMI. If successful, this trial will provide persuasive evidence for use of a practical, comprehensive CVD risk reduction strategy in SMI to reduce health disparities.

2. Objectives (include all primary and secondary objectives)

Accomplishing meaningful reductions in CVD throughout the US population will require implementation of targeted interventions that focus on vulnerable, high-risk groups such as those with serious mental illness. In this context, we propose the following Specific Aims:

Primary Aim:

1. Test the hypothesis that the active IDEAL intervention is more effective than the control condition in reducing CVD risk using the global Framingham Risk Score (FRS).

Secondary Aims:

2. Determine the effect of the active intervention compared to control on AHA metrics for ideal CV health risk behaviors and health risk factors:
    a. Weight and BMI;
    b. Physical fitness with 6-minute walk test;
    c. Healthy diet measured by self-report;
    d. Smoking cessation confirmed with urine cotinine;
    e. Fasting plasma glucose levels and diabetes mellitus treated to goal (HgBA1c);
    f. Blood pressure levels and hypertension treated to goal;
    g. Lipid levels (total cholesterol, LDL-C, HDL-C, triglycerides) and dyslipidemia treated to goal;
3. Determine the effects of the active intervention on other outcomes: health status (SF-12); quality of life (Euroqol); medication use for CVD risk factors; and medication adherence.
4. Assess costs per participant and perform a cost effectiveness analysis.

Exploratory Aims:

5. Conduct analyses to identify potential mediator variables (e.g., intervention participation, medication use for CVD risk factors and adherence);
6. Assess organizational factors that could facilitate the most effective future dissemination.
7. Evaluate outcomes at 24 months, 6 months post-intervention.

The primary outcome variable will be reduction in CVD risk with global FRS from randomization to 18 months. Six-month FRS and 6 and 18-month changes in health risks comprise secondary outcomes.

3. Background (briefly describe pre-clinical and clinical data, current experience with procedures, drug or device, and any other relevant information to justify the research)

Public Health Burden of Cardiovascular Disease in Persons with Serious Mental Illness

Persons with SMI, over 5 percent of the population or 15 million Americans, comprise a high-risk group for CVD-related mortality with rates approximately twice those of the overall US.[1-5] SMI frequently produces challenges in everyday functioning due to cognitive impairment and ongoing psychiatric symptoms;[6] accordingly, populations with SMI are often characterized by socioeconomic and environmental risk factors for CVD such as unemployment, low-income and social isolation.[7] The AHA Strategic Plan (co-authored by Dr. Appel) defines 7 goals for ideal CV health for all Americans including ideal health behaviors (BMI <25 kg/m^2, physical activity at goal levels, healthy diet, nonsmoking) and ideal health factors (fasting blood glucose <100mg/dl, untreated blood pressure <120/<80mmHg, untreated total cholesterol <200mg/dl).[8] Persons with SMI experience increased prevalence of unhealthy levels of these CVD risk factors, summarized below.

Overweight and obesity are at epidemic levels in SMI, particularly in women, where 60% are obese.[9-12] Physical inactivity and unhealthy diet contribute. The vast majority of SMI require ≥1 long-term psychotropic medication, yet many psychotropic classes cause weight gain, in part from increased appetite.[13-17] In particular, 2nd generation antipsychotics lead to substantial weight increases; in the case of olanzapine, up to 0.9 kg/mo.[18]

Physical inactivity is prevalent in SMI; Dr. Daumit reported persons with SMI report 50% higher leisure time inactivity than the general population.[19] An accelerometry study showed only 4% of persons with SMI in a psychiatric rehabilitation program (PRP) met recommended moderate to vigorous physical activity in bouts of ≥10 minutes.[20] Lack of affordable, safe places to exercise, and negative affective states such as depression may be important barriers.

Unhealthy diet is reported in persons with SMI with some studies reporting higher fat and lower fruit and vegetable intake and others show higher overall caloric intake.[21-23] Persons with SMI are also likely to depend on others for meals; preliminary baseline data from Dr. Daumit's study show 40% of participants relying on others to buy or prepare food when they eat at home.

Tobacco smoking is extremely prevalent in SMI, with 70 to 80% reporting lifetime and half to more than two-thirds reporting current smoking.[22,24-26] Smokers with SMI report using more and higher tar cigarettes and consuming more nicotine compared with other US smokers.[27,28] Smoking may be reinforced in SMI by lessening extrapyramidal effects from decreased blood antipsychotic levels, enhanced cognitive functioning from nicotine, socioeconomic factors and relative lack of other pleasurable pursuits.[26,29]

Diabetes mellitus is estimated ≥ 1.5-2 times higher in SMI than the overall population.[30-35] Multiple factors converge to increase risk of diabetes, and related emerging risks of glucose intolerance/insulin resistance and the metabolic syndrome in SMI including obesity and physical inactivity. Several antipsychotic medications also are implicated generally in proportion to their impact on weight gain and adiposity.[18,30,36-40]

Hypertension prevalence is 50% higher in persons with SMI than those without.[24] Obesity, physical inactivity, and alcohol use are likely contributing factors to elevated blood pressure.[34,41-44]

Dyslipidemias are also prevalent in SMI; in particular, certain antipsychotics lead to elevated triglycerides and low HDL.[18,34,45-49] With glucose intolerance, hypertension, dyslipidemia and abdominal adiposity all increased in SMI, metabolic syndrome prevalence is correspondingly 1.5 to 2 times higher in SMI as well.[34,50]

Interventions to Reduce CVD Risk in Persons with SMI

In order to accomplish meaningful reductions in CVD risk, targeted interventions will be required for populations with SMI, who urgently need to adopt healthy behaviors and decrease health risk factors.[51] Lifestyle interventions require tailoring for many with SMI, where cognitive deficits as well as other competing demands (e.g., psychiatric symptoms, economic stresses) are highly prevalent. Although few studies have been conducted in PRPs, these facilities are well suited for implementing comprehensive CVD risk reduction interventions as infrastructure facilitates collaboration with staff for patients with SMI.

Interventions to Improve Health Risk Behaviors:

Weight loss and physical activity lifestyle interventions including those addressing multiple health behaviors are effective in the general population, yet these trials systematically exclude persons with SMI who have pressing need. [59]A few published trials of behavioral weight loss interventions in SMI have shown success; most focus on diet changes alone and are short-term.[53-59] Dr. Daumit is a leader in developing and testing state of the art programs for diet and physical activity for SMI in community settings.[60,61]

Tobacco cessation interventions are underutilized for persons with SMI despite their modest success with this population. A recent literature review reported more than half of smokers with mental illness are contemplating quitting within 6 months or planning to quit in the next 30 days.[60] The 2009 evidence-based PORT schizophrenia treatment recommendations, co-authored by Dr. Dickerson, recommend smoking cessation treatment, specifically a psychosocial intervention and bupropion +/- nicotine replacement therapy (NRT).[61] These treatments produce evidence of smoking reduction and up to 33% abstinence.[62-70] Attendance levels in smoking cessation programs are associated with abstinence outcomes.[65] Individualized rather than group interventions could improve cessation rates. To-date, interventions addressing all major CVD behavioral risks comprehensively have not been tested in persons with SMI.

Interventions to Improve Health Risk Factors:

In the general population, there is substantial evidence that non-physician interventionists (e.g., care managers) facilitating treatment and coordination with PCPs can improve single and multiple CVD risk factors.[86-92] These interventions are thought beneficial by addressing patient environment and psychosocial factors and decreasing barriers to medication adherence.[91] Other evidence shows interventions focusing directly on medication adherence in primary care are also effective in improving risk factors.[93,94]

Trials concentrating on multiple CVD risks have not targeted SMI. In SMI, a small literature describes interventions in mental health clinics to improve primary care delivery including co-locating primary care (e.g., with nurse practitioner) or a care management model facilitating preventive

screening and referral to primary care.[95-98] These programs generally focus on overall health status and have not been specifically designed or staffed (i.e., with skilled interventionist) to effect changes in multiple CVD risk behaviors and factors.

Summary of significance: Persons with SMI are at extremely high risk for CVD morbidity and mortality. Previous studies have not tested comprehensive CVD risk reduction programs in this vulnerable population. Unless effective interventions are developed and tested, this population will continue to lag far behind the nation in CVD goals, and disparities will likely persist if not worsen. If the CVD risk reduction goal that the trial aims to attain could be applied widely to the millions with SMI in the US, tens of thousand of lives could be saved each year.[3,105-107] Psychiatric rehabilitation programs (PRPs) provide an available, yet untapped structure for multifactor CVD risk reduction interventions in SMI.

4. Study Procedures

a. Study design, including the sequence and timing of study procedures (distinguish research procedures from those that are part of routine care).

The IDEAL Trial is a community organization-based, two-arm clinical trial that will test the hypothesis that an 18-month comprehensive, practical CVD risk reduction program will be superior to control in improving CV health in persons with SMI. We will enroll 250 persons with SMI who attend 4 psychiatric rehabilitation day programs or adjoining mental health clinics. We will provide a common background of exercise classes and healthy changes to meals served. Participants will then be randomized to receive this control condition or the 18-month active IDEAL intervention with a Heart Health Interventionist, who will provide on-site: 1) individual CVD risk reduction behavioral counseling (e.g., for smoking cessation, diet); 2) coordination with primary care providers to ensure appropriate management of risk factors (e.g., blood pressure control); and 3) collaboration with mental health staff and social supports to encourage and motivate participants to reach goals.

Intervention:

Common Background: To establish environments conducive to healthy behaviors and standardized across sites, the study will provide resources for group physical activity classes open to all participants, and a dietician to consult to improve the health of meals served. Group exercise classes focusing on aerobic conditioning will follow a progression appropriate for sedentary adults, gradually building to 40 minutes of moderate intensity physical activity (3x/wk), and be designed to build exercise confidence.[108] We have used this successfully in SMI.[70,109,110] In order to support active intervention group participants' ability to select healthy foods, the intervention director will work with PRP staff to identify healthier menu choices, so that food served is more consistent with AHA Healthy Steps. The intervention director will be sensitive to budgetary issues and federal food guidelines. This will make available a needed foundation for physical activity and healthy diet choices to occur. However, these offerings are very unlikely to be sufficient alone for significant behavior change.

Randomized groups:

Control condition: The control group will receive this common background.

Active intervention: The active group will receive this common background and the IDEAL intervention.

Figure 1 displays our active IDEAL intervention framework. PRP staff, social supports, mental health providers (e.g., psychiatrists) and PCPs already have relationships with individual patients. For a participant randomized to the IDEAL intervention, the Heart Health interventionist (HHI) supervised by the Heart Health Clinical Consultant (HHCC) (nurse) works directly with the participant on health risk behaviors and catalyzes support from others, serving as a bridge between these individuals and the participant in moving toward ideal health risk behaviors and factors, resulting in improved CV health and quality of life.

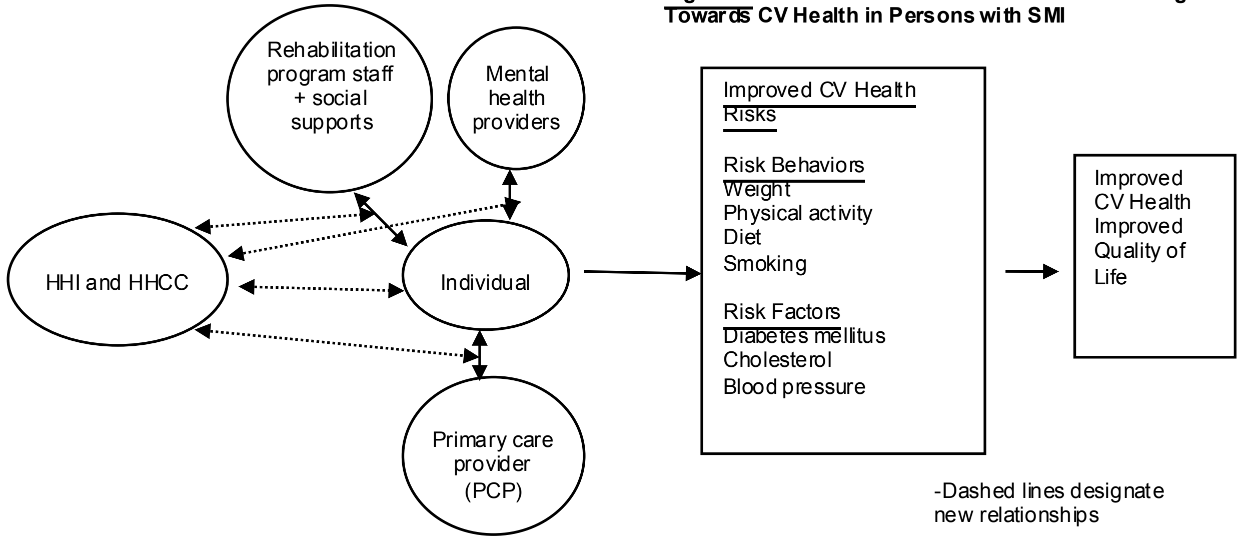

Figure 1. IDEAL Intervention Framework for Moving Towards CV Health in Persons with SMI

IDEAL Intervention goals include heart healthy behaviors (weight loss, physical activity, diet, smoking cessation) and heart healthy factors (blood sugar, BP, cholesterol) (Table). We use AHA goals and modify some (e.g.,10 lb. weight loss instead of BMI<25) to provide intermediate targets achievable with the study sample over 18-months.[8] Strategies combine individual counseling and goal-setting, consulting with the PCP to optimize CV risk factor treatment, and working with PRP staff, social supports and mental health providers.

Heart Health Interventionists (HHIs) coordinate resources and work with participants to develop tailored strategies to achieve the heart healthy goals. Interventionists will be physically located on-site at Mosaic. Embedding HHIs at community sites will enable optimal coordination of heart healthy planning with other PRP staff and allow HHIs the opportunity, in addition to scheduled sessions, to take advantage of everyday occurrences to reinforce healthy choices and behavior change (e.g., encouraging participant to go to exercise class, checking in and giving positive reinforcement for reporting decreased cigarette use, healthy vending machine choice or improved medication adherence, rewarding a participant for self-monitoring at a weigh-in). HHIs will be skilled facilitators with training in health behavior change and individual-level counseling, with a skill level typical for a community health educator, and will not require a Master's degree.[111] HHIs will receive training,[112,113] content supervision and will have ongoing, regular interaction with the Intervention Director and Heart Heath Clinical Consultant. The HHI is modeled after a position that would be feasible and sustainable in a community mental health setting, with back-up nurse support.

Heart Healthy Clinical Consultant (HHCC) will be a Registered Nurse or equivalent with primary responsibility to support HHIs by providing training and serving as an ongoing clinical resource. Weekly, the HHCC and HHIs will review participants' progress on heart health factors. He/she will support HHIs in their collaboration with PCPs and in implementing participants' Heart Health Treatment Plans. The HHCC will be trained in nicotine replacement therapy and will provide NRT and education and monitoring to interested and eligible participants using established guidelines (Treating Tobacco Use and Dependence: 2008).

Heart Health Sessions (20-30 mins.) will be held at least weekly for up to 6 months, then at least bi-weekly for 12-15 months with the HHI and the participant. The initial sessions will focus on building rapport and using motivational interviewing to review current heart healthy risk behaviors and factors, medication adherence, and to identify individual goals. Guided by the FRS score, the participant's abilities and interests, risks and targeted behaviors will be addressed either simultaneously or sequentially.[114] (See Table 2). Participants will work with their HHI to identify weekly behavioral changes topics from a list of recommended sessions (e.g., healthy weight management, becoming smoke free, staying physically active). The HHI will use the Heart Health Report Cards and solution-focused therapy to encourage attainment of individual goals and provide reinforcements. We are successfully using these techniques in ACHIEVE. Participants desiring to quit

smoking will be invited to smoking cessation groups if there are sufficient individuals at the same stage of quitting at each site.

Primary Care Provider (PCP) and Psychiatrist Coordination: The HHI will review the participant's baseline labs, physical measurements and medications with the HHCC and then initiate contact with the PCP to share data on baseline CVD risk factors and on any uncontrolled CV health risks and medication adherence. (Appendix 3F). The HHI will accompany the participant on a visit to the PCP in the community in order to facilitate a collaborative relationship and assist the participant in reaching CV health goals. The Heart Health Treatment Plan will be used to identify modifiable components of the FRS taking into consideration patients' preferences for change. The HHI will follow up with the PCP and psychiatrist as needed to ensure coordination of behavioral strategies and any pharmacologic treatment and coordinate additional PCP visits as needed. Guidelines for DM, HTN, and lipid control consistent with AHA, JNCVII, ATP-III and ADA[30,41,115-117] (and when available, the new NHLBI guidelines) are made available to PCPs, yet in our experience in ACHIEVE, PCPs are well aware of prevailing guidelines. They do, however, appreciate the initiative to coordinate care because SMI patients often have communication barriers, may have medication adherence issues due in part to lack of medication reconciliation across service settings, and often require advocates to make appointments and follow-up on tests. The HHI will manage this coordination for improved CV health. The HHCC nurse will work with the intervention team to communicate with PCPs and psychiatrists to advocate for bupropion or other pharmacotherapy for smoking cessation.

Staff and Social Supports Coordination: The HHI serves as a change agent, leveraging PRP staff and social supports around the participant's CV health goals. (Figure 1) HHIs will attend regular PRP staff case management meetings, which include residential staff for participants in supported housing. Here the HHI shares plans from the PCP and updates staff on participants' progress with the Heart Health Report Card and Treatment Plan. Having the HHI on-site enables two-way communication about participant CV health: 1) The HHI encourages staff to promote adherence to heart health recommendations (e.g., attending exercise class) and individually identified plans (e.g., setting quit date); 2) PRP staff inform HHIs about ongoing issues that could interfere with participant progress towards CV health goals (e.g., change in living situation).The HHIs also will encourage social support. They will pair interested participants with an exercise buddy to provide support in groups and for exercise outside of the PRP.[110-113] HHIs also will match participants working towards smoking cessation with a former smoker with SMI.[114] In addition, participants will be encouraged to identify family members or other social supports, (e.g., caregiver, friend) to assist them in adoption of healthy lifestyles. We will offer quarterly telephone consults to these social supports to help them learn more about Heart Healthy Habits and how they can support the participant. Through all of these pathways, the HHI coordinates a team-based approach to improve participants' CV health.

Mental Health Provider Coordination: Participants have therapists and psychiatrists they see for mental care and psychotropic medication treatment; for the most part they will be in the adjoining mental health clinics. The HHI will share the Heart Health Treatment Plan with them. Psychotropic prescribing practices are not specific intervention targets, however, the psychiatrist may decide to modify medications based on the participant's CVD risks. The HHI will facilitate communication between the psychiatrist and PCP.

Program materials: Individual visit manuals will be developed to guide HHIs in conducting sessions based on our prior work. The Heart Health Report Card facilitates HHI and participant monitoring of behavioral goals. It allows the HHI to keep track of the participants' changes in weight, exercise attendance and additional tailored goals and helps to monitor the status of heart healthy factors (blood sugar, BP and lipids). Cards will be updated at the individual sessions with the corresponding reinforcement points associated with recent accomplishments. The Heart Health Treatment Plan indicates the participant's current status and treatment plan for the 7 CVD risk factors. It serves as an instrument to coordinate care with participants, PCPs, PRP staff and mental health providers.

Self-monitoring: Participants will be encouraged to self-monitor eating, exercise and smoking behaviors as appropriate to their individually tailored behavioral goals and cognitive abilities. This data will be reviewed with participants at individual visits and used to update Heart Health Report Cards.

Reinforcements: We plan a points system to reinforce behavior change. Points are awarded for recommended behaviors (e.g., decreased cigarette smoking, group exercise) and can be traded for cheaper items (e.g., pen, key chain) or saved for larger rewards (e.g., CD player). Rewards are used to reinforce healthy habits that forgo immediate gratification (e.g., eating candy now) for larger, delayed gratification (e.g.,weight maintenance). Process measures: For active intervention participants, we will track session completion, exercise class participation, medication adherence, and interim progress towards goals (e.g.,

weight, pedometer counts, expired CO) for participant feedback, case management and analysis of potential mediators.

Table 2. Features of IDEAL Active Intervention

CVD Risk Targets	AHA Strategic Goals (modified for IDEAL)		Traditional Behavioral Modification Approach	Behavioral Modification Approach Targeted to SMI
Healthy Weight	Weight: BMI<25 kg/m^{2*}<br><br>(Encourage 10 lb. weight loss. Normal weight participants encouraged to maintain weight).		-Participant identifies focus of change<br>-Self-reward	-HHI weighs participant and monitors weight progress<br>-HHI directs high impact behavioral target<br>-HHI rewards behavior<br>-Physically change foods available at PRP<br>-Provide exercise classes at PRP, reward participation
Physical Activity	Physical Activity: 150+ min/wk moderate		-Health education<br>-Role of social support	-Provide<br>-Provide pedometer and instructions, reinforce use
Diet	Healthy Diet: 4-5 AHA components<br>(DASH modified with <1500 mg/day Na, <7% energy from saturated fat, no sugar beverages)		-DASH Diet education<br>-Participant identifies focus of dietary change<br>-Role of social support	-Simplified diet education messages<br>-Wallet size card with dietary messages<br>-HHI recommends specific dietary targets<br>-HHI communicates with residential staff/social supports<br>-Pre-printed shopping lists<br>-"Field Trips" to grocery store
Smoking	Stop smoking (smoking cessation but also will realize quit attempts and decreased number of cigarettes/day)		-Provide general information about risks of smoking and benefits of quitting	-Counseling (App. 3B) with increased contact, 2x/ week<br>-Motivational interviewing and 5R's<br>-HHI communicates with PRP staff, residential program counselor and/or home social support about goals and strategies for smoking cessation<br>-Facilitate access/provide nicotine replacement therapy (NRT) and work with PCP and individual to incorporate NRT into treatment plan, communicate with PCP and psychiatrist to advocate for pharmacotherapy for smoking cessation including varenicline, bupropion and NRT<br>-Contingency management with CO testing<br>-HHI may conduct small smoking cessation groups
Control Blood Sugar	Fasting glucose: <100 or HbA1c<7% for diabetes		-Heath education<br>-Self monitoring: carbohydrate intake and blood sugar<br>-Problem solving to increase medication and diet adherence	-Teach/reinforce self-monitoring of blood sugar<br>-Simplified dietary messages: (e.g., "No sugar drinks")<br>-Solution-focused therapy for medication adherence. -Provide weekly pill organizers<br>-HHI and HHCC coordinates with PCP regarding HbA1c targets, other testing (e.g., proteinuria) and any needed medication changes due to weight loss/exercise<br>-HHI communicates with PRP staff, residential counselor and/or home social support about diet and medication<br>-Provide exercise classes at PRP, reward participation
Control BP	Blood Pressure <120/80 or treated to goal		-Heath education<br>-Self monitoring: sodium intake and blood pressure<br>-Problem solving to increase medication and diet adherence	-Simplified messages ("Avoid packaged/canned foods")<br>-Solution-focused therapy for medication adherence -Provide weekly pill organizers<br>-HHI and HHCC coordinates with PCP about BP therapeutic goals and any medication adjustments<br>-HHI communicates with PRP staff, residential counselor and/or home social support about diet and medication<br>-Provide exerciseclasses at PRP, reward participation
Lower Cholesterol	Total Cholesterol <200 or treated to goal		-Heath education<br>-Self monitoring: calorie and fat intake<br>-Problem solving to increase medication and diet adherence	-Simplified dietary messages (e.g., "Avoid junk food")<br>-Solution-focused therapy for medication adherence -Provide weekly pill organizers<br>-HHI and HHCC coordinates with PCP regarding cholesterol therapeutic goals and medication<br>-HHI communicates with PRP staff, residential counselor and/or home social support about diet and medication<br>-Provide exercise classes at PRP, reward participation

b. Study duration and number of study visits required of research participants.

Eligibility and Baseline

Participant eligibility is determined by completion of several screening measures. Data collected at these visits provide baseline information and classifies individuals for subgroup analyses. Screening visits will occur on-site at Mosaic rehabilitation centers and outpatient clinics. Data collectors will be on-site regularly during the screening and follow-up periods and measures will be performed around participants' needs and schedules. We plan to complete screening measures within six to eight visits, ranging from 20 to 60 minutes each over the course of 4 to 8 weeks. Research staff will have flexibility in determining which screening measures will be completed at which visit for each participant. Study instruments are administered in-person by data collectors.

All participants provide written informed consent for entry into the trial. We will use a two-stage consent procedure: one consent form will be signed upon entry that covers screening and baseline data collection; another will be signed at the time of randomization and study enrollment. In consenting individuals to participate in study, the following procedures will be used: 1. All procedures involved in the study will be fully explained to participants by staff trained in informed consent procedures for patients with severe mental illness. 2. An evaluation of ability to give consent is also administered for each participant which includes having potential participants answer questions about the goal of the study, what they will be asked to do if they decide to join the study, and what risks may be involved if they join the study. If a participant is deemed not able to give consent, he/she may not participate in the study. 3. Those agreeing to participate will sign the consent form."

We will obtain consent in a private room at the behavioral health organization. We allot 30 minutes per participant, and in our experience in the ACHIEVE Trial this is ample time and more commonly 15-20 minutes is needed. We will give potential participants the consent form in advance to read while waiting to meet with a study staff.

After baseline data collection and before the intervention begins, all screened, potential participants will meet with study staff and the enrollment/intervention part of the study will be described in detail. Those interested in enrollment will be consented individually and randomized to either the intervention or the usual care/control group.

Randomization to the IDEAL intervention or control will be stratified by site. To prevent predictability of assignment, the randomization schedule will be created in variable block sizes.

Follow-up

We will collect follow-up measures at 6, 12, 18 and 24 months after baseline with in-person contacts at the study sites.

c. Measurements. Measurements will be conducted using standardized operating procedures and quality control methods. Specific study forms will be used to collect data. Table 3 summarizes the data collection schedule.

Detailed measures are described below.

Demographics and Medical History: We will collect demographic and contact information, medical history with a short checklist of conditions, Rose Angina Questionnaire, and assess alcohol or substance use using the ASI-Lite.[125,126]

Mental health diagnoses and medications will be abstracted from PRP (or mental health clinic) charts. Participants and providers will confirm medications.

Physical measures.

Blood pressure will be determined by the OMRON 907 XL, a validated device which records BP using an oscillometric technique.[127] Blood pressure will be obtained by trained, certified data collectors. On each of 3

visits, 1 week apart, 3 measurements (each separated by 30 seconds) will be obtained on the right arm of participants after they rest quietly in the seated position for at least 5 minutes.[128]
Weight will be measured to the nearest 0.1 lb by a high quality digital scale with participants wearing light indoor clothes without shoes. Duplicate measurements will be made. Weight will be measured in lbs. for ease of interpretation by participants and converted to kg for calculation of BMI, calculated as the Quetelet index (kg/m^2).
Height to the nearest 0.1 cm will be measured at entry using a wall-mounted stadiometer. The participant stands shoeless on a firm, level surface, with head in the horizontal (Frankfort) plane.
Waist circumference will be measured with an anthropometric tape, in a horizontal plane 1 cm above the navel.
Physical fitness will be measured by the 6-minute walk test. This measure is a valid predictor of VO2 max and has been shown to be responsive to increases in regular moderate physical activity.[129-131]
Fasting blood measures will be collected at Mosaic, centrifuged, aliquoted and sent for processing. Total and HDL cholesterol, triglycerides, glucose, Hemoglobin A1C will be measured directly, and LDL cholesterol will be estimated by Friedwald equation unless direct measurement is needed.[132] Serum CRP and insulin, kidney function (creatinine) and serum carotenoids (lutein and zeaxanthin) will also be measured.[133-135] We will measure antibodies to viruses and bacteria such as herpes simplex virus and toxoplasma as previous studies have found an association between antibodies to infectious agents and clinical outcomes in serious mental illness. Blood from part of the collected specimens will be frozen at -70° at Johns Hopkins for future investigation of putative risk factors related to cardiovascular disease. Candidate assays include inflammatory markers, leptin and adiponectin. We also will store samples of whole blood at baseline for DNA for later studies. As this study will collect detailed information on medication, blood pressure, BMI, tobacco smoking and traditional laboratory markers of cardiovascular risk pre/post intervention, future analyses of stored serum samples will be able to be interpreted in the valuable context of these other risk factors. Approximately seven teaspoons of blood total will be collected at each study time point with one teaspoon stored for future investigation and ½ teaspoon stored for future DNA testing (DNA storage at baseline only). We plan to store blood samples indefinitely.

Potential participants have specific choices on the consent form to decline or agree to have their blood stored and to DNA testing. The serum samples will be identified with a code that does not include any identifying participant information and will be stored in a secure Johns Hopkins facility. There are currently no plans to re-contact subjects regarding their individual stored serum samples, and we will not release the results of future tests from serum samples to individual subjects. We believe these procedures described above constitute minimal risk for the participants. Study samples may be shared with other researchers partnering with the study team for future research. If we do share any study samples with outside investigators not directly addressing the research questions in this protocol, an amendment/change in research or a new protocol would be submitted to the IRB.

Urine will be collected for cotinine, to confirm smoking status, and for Na, K, creatinine and protein.
Urine will also be collected for Na, K, creatinine and protein.

Expired carbon monoxide from the piCO Smokerlyzer (Bedfont Scientific Limited) will also be collected.[126]
Physical activity will be measured by accelerometry in addition to self-report. Objective measures are not subject to biases asociated with self-reported physical activity assessments but are limited in that they only assess physical activity at the time the monitor is worn. We will use the Actigraph activity monitor. The monitor, the size of a pager, is worn on the hip. Piezo-electric accelerometer technology measures motion in three dimensions and provides tri-axial vector data in activity count units, metabolic equivalent units (METs) or kilocalories. The monitor can be used to assess a global index of physical activity (i.e., total counts per day, time spent in moderate physical activity/day) and is reliable. Energy expenditure determined by Actigraph and from treadmill exercise correlates at r=0.89. Established cutpoints will be used to identify physical activity of at least moderate intensity. Accelerometers do not require user input beyond putting the unit on in the morning and taking it off before bed or before going in the water. We may select a sample of participants for accelerometry depending on project resources. Participants will wear accelerometers for 7 days.

Questionnaires will be used for a variety of purposes including baseline data to describe participants, outcomes to assess intervention effects, and mediating variables to assess potential causal pathways. The

study team has experience with these and similar instruments for persons with SMI.[70] Instruments are described here:

Healthy diet. Block Fat, Fruit, Vegetable and Fiber and Sodium Screener Questionnaires provide self-report of daily fruit, vegetable intake, and percent energy from fat.[139] We also will use a questionnaire on sugar sweetened beverages.

Tobacco smoking will be assessed with questions current smoking, 7-day and longer-term abstinence, Fagerstrom Test of Nicotine Dependence (FTND), a 6-item measure of behaviors related to dependence on nicotine, and self-efficacy questions related to quitting.[140,141 137,142]

Health status will be measured by the Medical Outcomes Study SF-12.[143]

Physical activity will be collected by self-report with the Godin Leisure Time Exercise Questionnaire.

Quality of life will be measured with the Euroqol EQ-5D, a brief 6 item instrument that is valid in persons with schizophrenia and bipolar disorder, and can be used for cost-effectiveness analyses.[144-146]

Medication adherence will be measured with an adapted questionnaire based on the Morisky Medication Adherence Scale for each class of medications for CVD risk factors (e.g., antihypertensives, lipid lowering medications).[147]

Mental health symptoms will be assessed by The Center for Epidemiologic Studies Depression Scale (CES-D) to measure depressive symptoms[148,149] and the Behavior and Symptom Identification Scale (BASIS-24), a brief comprehensive mental status measure for overall mental health status.[150-152]

Social Support will be measured with the Medical Outcomes Study Social Support Questionnaire[153,154]

Other health measures related to CVD risk relevant in SMI population. We will use the Neighborhood Questionnaire to assess participants' home environments for safe places to exercise and places to purchase healthy food, a questionnaire on food and shopping habits, the Questionnaire for Eating and Weight Patterns-Revised to measure Binge Eating, and the Pittsburgh Sleep Quality Index.[155-157] We will assess health literacy with the Health Literacy Skills Instrument.

Participant semi-structured interviews (approximately 15-20 minutes) will be conducted at the end of the 18-month intervention to gain an understanding of participant perceptions of and satisfaction with the heart health program and exercise classes. Interviews will be conducted with a convenience sample of participants across the study locations. Interviews will be audio-recorded and transcribed. Transcripts will not contain names and will be kept in a secure location. Audiotapes will be destroyed after transcription is completed.

**Table 3: Data Collection Schedule**	Baseline	6 mo	12 mo	18 mo
Informed consent	X			
Contact information	X	X	X	X
Demographics	X	X		X
Medical conditions, substance use	X	X		X
Mental heath diagnoses	X			
Medications	X	X	x	X
Physical measures				
Blood pressure	X	X		X
Weight	X	X	x	X
Height	X			
Waist circumference	X	X		X
6 minute fitness walk	X	X		X
Fasting blood measures	X	X		X
Urine measures	x	x		x
Expired Carbon Monoxide	X	X		X
Accclerometery	x	x		x
Questionnaires				
Healthy diet	X	X		X
Tobacco measures	X	X	x	X
Physical activity	X	X		X

Health status	X	X		X
Quality of Life	X	X		X
Medication Adherence	X	X		X
Mental health symptoms	X	X		X
Social Support	X	X		X
Other-Neighborhood, Binge Eating, Sleep, Health literacy	X	X		X
Semi-structured interview				x
Event surveillance		X	X	X

Event surveillance. We will collect data related to health care utilization and medical records related to CVD outcomes of interest. This will include consent documents and medical release forms with appropriate text and a surveillance process that is applied identically to each randomized group.

Food environment: We will analyze a random sample of menus at baseline, 6 and 18 mos. and use ESHA software to assess changes in total calories/nutrients in site meals as a result of dietary consultation.[158]

Staff semi-structured interviews for organizational factors: To better understand the intervention implementation process and perspectives of intervention staff, non-intervention staff and leadership at Mosaic concerning the sustainability and future acceptability for dissemination of IDEAL, we will conduct semi-structured interviews. We will ask about the intervention's perceived effectiveness, ease of or barriers, fit within the organization and financial models for continued support. We will interview before the intervention for baseline information and then at end of the 18-month intervention to assess changes due to the intervention. We will gain an understanding of factors that impact the intervention being acceptable and workable, and prepare for dissemination to other organizations. We expect to conduct interviews with 75 people, or a total of 150 interviews. Questions will include topics of perceived effectiveness of the intervention, ease or barriers for the intervention, fit within the organization and suggestions for future dissemination. The interview will be approximately 30 minutes in length.
Interviews will be audio-recorded and transcribed. Transcripts will not contain names and will be kept in a secure location by Dr. Daumit. Audiotapes will be destroyed after transcription is completed. Individuals will not be identified by name in any publication. Mosaic leadership agrees that staff participation in the interview and any information shared will not affect employment or evaluation at Mosaic.

d. Blinding, including justification for blinding or not blinding the trial, if applicable.

Randomization to the IDEAL intervention or control will be stratified by gender and site. To prevent predictability of assignment, the randomization schedule will be created in variable block sizes. . Prior to obtaining the assignment, the study coordinator will confirm the participant meets all eligibility criteria and all required baseline data have been collected. Due to the nature of the intervention, participants and interventionists will be aware of group assignment, however, data collection staff will be kept blinded. The random sequence and allocation envelopes will be created and kept private by the data analyst; the study coordinator will then ascertain and communicate treatment assignment to participants. Due to the nature of the intervention, both participants and interventionists will be aware of the assignment. Until the trial end, investigators, other staff and participants are masked to outcome data, with the exception of trial statistician, study coordinatorand data analyst.

e. Justification of why participants will not receive routine care or will have current therapy stopped. N/A

f. Justification for inclusion of a placebo or non-treatment group.
All participants, control and active intervention, will have access to group exercise classes. The control participants are needed to test the benefit of the comprehensive CVD risk intervention.

g. Definition of treatment failure or participant removal criteria.
We will continue to follow participants for data collection whether or not they are attending the intervention sessions unless they develop an study exclusion criteria (e.g., pregnancy) that would require they are censored from the study.

h. Description of what happens to participants receiving therapy when study ends or if a participant's participation in the study ends prematurely.
N/A.

Table 4: Eligibility Criteria
Inclusion criteria
▪ Age 18 and older
▪ Body mass index at least 25 kg/m^2 OR<br>one of the following CVD risk factors:<br>-Hypertension (SBP>= 140mmHg or DBP>= 90mmHg or on antihypertensive medications;<br>-Diabetes mellitus (fasting blood sugar> 125mg/dl or hemoglobin A1c>6.5 or on a hypoglycemic medication);<br>-Dyslipidemia (LDL >130 mg/dl , HDL<40 or total cholesterol >=200 or on a lipid lowering agent);<br>-Current tobacco smoker
▪ Able and willing to give informed consent
▪ Completion of baseline data collection
▪ Willing to accept randomization
▪ Willing to participate in the intervention
Exclusion criteria
▪ Cardiovascular event (unstable angina, myocardial infarction) within the past 6 months
▪ Serious medical condition which either limits life expectancy or requires active management (e.g., certain cancers)
▪ Condition which interferes with outcome measurement (e.g., dialysis)
▪ Pregnant or planning a pregnancy during study period. Nursing mothers would need approval from physician.
▪ Alcohol or substance use disorder if not sober/abstinent for 30 days
▪ Planning to leave rehabilitation center or clinic within 6 months or move out of geographic area within 18 months
▪ Investigator judgment (e.g., for concerns about participant or staff safety)

5. Inclusion/Exclusion Criteria

The study population will consist of 250 adults, ages 18 and older from 4 psychiatric rehabilitation programs and affiliated outpatient mental health clinics. Table 4 lists eligibility criteria.

6. Drugs/ Substances/ Devices- N/A

a. The rationale for choosing the drug and dose or for choosing the device to be used.

Nicotine patches and nicotine lozenges are FDA approved and available as over the counter pharmacotherapy for tobacco cessation. For interested participants in the active intervention arm who are smokers and would like to quit, we will provide and supervise the use of nicotine patches and nicotine lozenges as nicotine replacement therapy for them. Nicotine patches are dosed every 24 hours; nicotine lozenges (2 and 4mg doses) are used as needed initially every 1-2 hours and then tapering down to every 4-8 hours over several weeks. Nicotine patch initial dosing is based on the level of smoking at baseline, with 21mg generally used for those smoking 1 pack of cigarettes per day; 44mg may be used for those smoking 2 packs per day. Patches and lozenges may be used in combination.

b. Justification and safety information if FDA approved drugs will be administered for non-FDA approved indications or if doses or routes of administration or participant populations are changed.

c. Justification and safety information if non-FDA approved drugs without an IND will be administered.

7. **Study Statistics**

a. Primary outcome variable. The primary outcome, measured from randomization to 18 months, will be the risk of CVD from the global Framingham Risk Score (FRS) expressed as the 10-year probability of a CVD event.[159] In contrast to separate algorithms used to assess risk of specific events[160] (CHD, stroke, PVD and heart failure), the global FRS predicts risk of all CVD events. The global FRS consists of validated, sex-specific multivariable risk functions that include terms for age, total cholesterol, HDL-cholesterol, systolic BP, smoking, diabetes, and treatment for HTN. In clinical practice, such scores have been incorporated into guidelines (e.g., ATPIII) as a means to risk stratify individuals and guide treatment decisions.[116] In research, scores have been used by our group and others to document the overall effects of behavioral and practice-based interventions on CVD risk, considering concomitant changes in all relevant risk factors (systolic BP, total cholesterol, HDL cholesterol, smoking status and diabetes status).[161-164] Six-month FRS is a secondary outcome.

Secondary outcome variables. Weight and BMI;Physical fitness with 6-minute walk test; Healthy diet measured by self-report and serum carotenoids; Smoking cessation confirmed with urine cotinine; Fasting plasma glucose levels and diabetes mellitus treated to goal (HgBA1c); Blood pressure levels and hypertension treated to goal; Lipid levels (total cholesterol, LDL-C, HDL-C, triglycerides) and dyslipidemia treated to goal

b. Statistical plan including sample size justification and interim data analysis.

Data Analysis: Two-hundred and fifty participants will be recruited from 4 sites in 1 health organization,enrolled, and randomized in equal allocation to active intervention (Group A) and control (Group B), stratified by site. Formal assessments are at baseline, 6, and 18 months. Primary analyses will be conducted under the intention to treat principle.[167]

Core analytical model**:** Let Y_{ijk} denote the log-transformed FRS for the ith participant at the jth visit (j = 0, 1, or 2), corresponding to the baseline, 6, and 18-month visits), in the kth treatment group (k = 0 or 1 corresponding to intervention groups A and B). X_{ik} is a vector of pre-randomization covariates (which will include study site indicators). The longitudinal regression model for the primary analysis is:$E[Y_{ijk}] = X_{ik}\eta + \mu + \alpha_j + \beta_k + \gamma_{jk} + e_{ijk}$ with the constraints that each of (α_j , β_k ,γ_{jk}) equals 0 when an index is as its lowest value. The e_{ijk} are independent unless the (i, k) subscripts are the same. For participant (i,k) the e_{ijk} have an unstructured 3×3 covariance matrix. The η measure the influence of baseline covariates, the α_j give visit effects within Group A, β_k baseline treatment effects (which under effective randomization should be close to 0) and the γ_{jk} are the treatment by visit interactions, "the treatment effects." The primary testing contrast is to compare baseline to 18-month changes in FRS (the primary outcome) between intervention groups, i.e. testing the hypothesis that $\gamma_{21} = 0$. Data at 6 months, though not the primary outcome, will help determine whether the changes over 18 months are linear. The primary analysis will follow the intention to treat principle and will not adjust for post randomization variables. This core model is also applicable to other secondary continuous outcomes such as weight, blood pressure and lipids. Modeling will be implemented via SAS PROC Mixed (or the Stata or R-equivalents). Site effects will be included to account for possible within-site clustering.

Missing and incomplete data: Prevention is far superior to statistical treatments; every effort will be made to collect outcome data on all randomized. The underlying missing data process determines the biasing effects of missing data and structures valid analytic strategies.[168] If the probability of a potential observation being missing depends on what has, but not on what hasn't been observed, termed missing at random (MAR), then estimates based on an appropriate analytic model (both the mean and error structure) for the observed data will not be biased. A valid model for the observed data allows the missing data process to be ignored. If the missing is informative, meaning that the probability of an observation being missing depends on the value of that observation, then bias cannot be avoided by modeling the observed data alone, and the impact of such bias would be evaluated using sensitivity analysis.

Sensitivity Analyses will be conducted with multiple imputation with respect to departures from the MAR assumption. The model will be derived from our primary analytical model under MAR, maintaining the covariance structure and modifying the imputation mean according to informative missing data scenarios.

Principal Secondary Analyses. Secondary outcomes*:* We will replicate the foregoing analyses formeasured outcomes (BP [raw scale], lipid levels and HgbA1c levels [log scale]. For dichotomous outcomes (e.g., HTN control, lipid control, diabetes control, smoking cessation) GEE based logistic regression modeling will be conducted using SAS Proc Genmod (or the Stata or R-equivalents) with a logistic link and a within-individual, unstructured correlation structure. We will use data for all participants to compare changes in outcomes over

time between intervention arms. For certain dichotomous outcomes we will include only “at risk” participants (e.g., smoking cessation in smokers). Medication use for CVD risk factors and psychotropics will be assessed and compared between arms and adherence examined using conventional methods.[169-171]

Exploratory Analyses: In addition to analyses that preserve the intention to treat principle, we will also perform informative exploratory analyses based on subgroups defined post-randomization to characterize aspects of the intervention that may be related to improved outcomes in the active group. Such analyses will use post-randomization variables that are potential mediators - interventionist visit and exercise class participation, medication modifications, medication adherence and self-monitoring activities.[172] For example, for patients with BP controlled at baseline with medications, medications may be lowered if lifestyle changes occur. As a result, gauging intervention effects based on measured BP or HTN control alone may underestimate such effects as effects could partially be reflected though reduced use of medications. We will develop a protocol for these analyses defining subgroups before the primary outcomes are collected. Even so, these analyses are more prone to biases so that protection from confounding afforded by randomization is not likely applicable. Propensity score stratifications will be used to manage potential confounding in these analyses. Nevertheless, these findings will be interpreted with caution and will be considered exploratory.

Qualitative Analysis: Interviews will be transcribed and coded. NVivo will be used to enter and group discrete passages from data into themes. We will use the constant comparative method[175] to categorize transcript statements that demonstrate common attributes, then combine categories into broader, recurrent themes using a hybrid approach where we will start with larger categories and then add nodes and subnodes as new concepts emerge until saturation of ideas is reached. Findings will be compiled to inform ongoing implementation and future sustainability and dissemination.

Power analyses: The trial has sufficient resources to enroll 250 participants. With this sample size, our objective was to determine the minimum detectable difference (MDD) in relative risk reduction in the global Framingham Risk Score (FRS). Our assumptions are as follows:

- Two-sided type I α error=0.05; type II β error=0.20; 90% follow-up.
- Baseline absolute CVD risk with global FRS =11% from ACHIEVE Trial (n=291, Table 1);
- Standard deviation of change in global FRS=3% from ongoing ACHIEVE Trial (from Dr. Daumit’s pilot weight loss intervention in SMI, similar SD of 2.9%);
- Global FRS change in controls=5% relative risk reduction. Controls could experience some reduction in CVD risk because of secular trends and offerings of physical activity classes on-site. However, aging increases CVD risk and might counterbalance these effects. We conservatively assume a 5% relative reduction in the control group, even though their risk might not change or might increase over time.

With these assumptions, our MDD is a 10.7% reduction in relative risk in global FRS.

Table 5: Calculation of Relative Risk Reduction to 10 year Framingham Risk Score from Baseline to Follow-up

	Baseline FRS Risk	Follow-up FRS Risk	Relative Reduction, within-group		Relative Reduction, between-group	Minimum Detectable Difference (MDD)
	10yr Probability of CVD Event		Relative Risk	Percent Risk Reduction	Relative Risk Ratio (RRR)	MDD=1-RRR
Active	11%	9.33%	9.33%/11%=0.848	1 - 0.848 = 0.152 (15.2%)	0.848/0.95=0.893	1 - 0.893 = 0.107 (10.7%)
Control	11%	10.45%	10.45%/11%=0.95	1 - 0.95 =0.05 (5%)		

Table 5 demonstrates the relation between baseline and 18-month follow-up and absolute and relative changes in global FRS with the example of a percent risk reduction in the control group of 5% and an MDD of 10.7%. The reduction in relative risk ratio of 10.7% (MDD= 1 – RRR) is very similar in magnitude to the direct difference in relative risks [95% - 84.8% = 10.2%] at 18 months. If risk is unchanged or rises in the controls, our MDD will be lower.

**Table 5.** Minimal Detectable Differences for Relative Framingham Risk Score Reduction by follow-up rates and % risk reduction in controls			
	MDD, assuming a Relative Reduction in Control Group of:		
Follow-up rate	**0%**	**2.5%**	**5%**
100%	9.7%	10.0%	10.1%
95%	9.9%	10.2%	10.4%
90%	10.3%	10.5%	**10.7%**
85%	10.5%	10.8%	11.1%
80%	10.9%	11.1%	11.4%

Table 6 displays MDD for ranges of follow-up rates and relative risk reduction in the control group. The MDD of 10.7% is very achievable. IDEAL will combine lifestyle/behavioral modification with improved clinical management of CVD risk factors. In studies of lifestyle interventions, reported relative reductions in CV risk scores range from 9-16%,[161,163,176] and for care management interventions, [91,177] from 12%-23%. Table 7 displays ranges of changes in individual CV risk factors and corresponding estimated changes in global FRS from interventions in the literature with components similar to IDEAL. Even with the understanding that intervention components will not likely be fully additive, and that except for smoking cessation, these estimates are from studies in the general population and not SMI, Table 7 provides support for many scenarios where the MDD in FRS of 10.7% would be attainable. We have precision for estimating clinically significant intervention effects in secondary outcomes. The 95%confidence interval (CI) for an effect estimate is +/-1.96* sqrt(2/n)*σ, where n is the sample size per group andσ the common SD for change in the measured outcome of interest for both groups. Assuming 90% follow-up, the 95% CI for the intervention effect of difference in change between groups would be: glucose 7.9 mg/dl; SBP 3.5mmHg, weight 4.0 lbs; total cholesterol 9.0 mg/dl; HDL 2.9mg/dl; SF-12 Physical Functioning 7.9. The SDs for change are derived from the ongoing ACHIEVE Trial. For smoking cessation, we assume 75 smokers per group with 1.3% quit rate in the controls (i.e., 1 of 75). We will have 80% power to detect a quit rate of 14% or higher in the active group using a 2-sided alpha of 0.05.

**Table 6.** Reported CVD risk factor changes and estimated relative reduction in global FRS		
Risk Factor	Reported range of change in risk factor	Corresponding Δ in Global FRS♯
SBP	Lifestyle interventions: 3.7-8mmHg [176,178,179]	5-15%
	Care management: 6-11mmHg [93,177,180]	6-19%
Total cholesterol	Lifestyle intervention: 19mg/dl [176]	10%
	Care management: 12.9-15.5 mg/dl [91,181]	7-7.5%
HDL	Lifestyle interventions: 2-2.4mg/dl[176,179]	4-5%
Smoking cessation	Psychosocial /buproprion +/-NRT* 12-33%[62-68]	1 -3.3%±
Fasting glucose/HgbA1C	Lifestyle interventions: HgbA1c 0.5-0.99[179,182]	
	Care management: fasting glucose, 7.7-20mg/dl, HbgA1c 1.1[91,177,183]	
*in SMI. ± After weighting by the anticipated prevalence of smoking. ♯ To estimate global FRS Δ, we used median risk factor levels in ACHIEVE. Then, for each risk factor separately, we applied literature-reported changes in that risk factor to the global FRS equation.		

7. **Risks**

a. Medical risks, listing all procedures, their major and minor risks and expected frequency.

The study should not involve any major risk to screenees and trial participants. Recommended treatments, both lifestyle and pharmacologic, are based on prevailing national guidelines. However, there are some risks. Sources of risk include medication-related adverse events, such as hypoglycemic events, side effects from medications for CVD risk factors prescribed by the patient's primary care physician and side effects from nicotine withdrawal or over-the-counter nicotine replacement therapy. For example, improved health behaviors from the intervention (e.g., physical activity, weight loss) may lead to hypoglycemia or hypotension in those already taking medications and necessitate adjusting diabetes or blood pressure medications. In addition, physical activity is associated with a small risk of cardiovascular complications (less than 1 per 187,500 person-hours of exercise).[184] Physical activity can also increase the risk of musculoskeletal discomfort or injury. Blood drawing may cause some discomfort and/or bruising at the site of the venipuncture. The methods that participants may use to restrict calories (e.g. extreme calorie restriction or unbalanced dietary pattern) may lead to inadequate energy intake or lower than recommended intake of essential nutrients. In addition, participants run the risk of a breach in confidentiality. These risks should be rare. Plans to minimize these risks are described below.

b. Steps taken to minimize the risks.

The safety approach will be implemented similar to the investigators' other trials (e.g., POWER Trial). We will have a medical safety officer, Dr. Miller. He will be in charge of developing the safety protocol for the trial, and he will promote and monitor safety throughout the study. As in our previous studies, the safety officer does not provide direct medical care and is not involved in the intervention. We will use standardized materials including a manual of procedures and forms. NHLBI has considered materials that we developed in the POWER Trial as models which are now used in other NHLBI-supported lifestyle intervention trials.

<u>Hypoglycemia, hypotension or other potential consequences of improved health behaviors or changes in medication:</u> These will be minimized by having the intervention team communicate closely with the primary care physician and rehabilitation program staff about medication changes, exercise participation and coordinate assistance for participants in blood sugar monitoring. For example, if an active intervention group participant is taking a sulfonylurea and has significantly increased exercise frequency, the intervention team will communicate with the PCP and raise the suggestion of a glucose-lowering agent that would have lower risk of hypoglycemia, such as metformin. The interventionist will also ensure appropriate follow-up with the PCP after any medication changes.

<u>Physical activity:</u> The small risk of physical activity will be minimized by emphasizing moderate (as opposed to vigorous) activity, and by following American College of Sports Medicine guidelines regarding need for medical examination prior to beginning an exercise program. Participants who wish to progress to vigorous activity will be advised to obtain approval from their primary care physician. Risk of injury is further minimized by instruction on proper exercise technique, the importance of warm-up and cool-down exercises, and proper stretching techniques.

<u>Blood drawing:</u> All phlebotomy is performed by an experienced phlebotomist. Participants will be given information on how to contact Dr. Daumit if complications occur.

<u>Extreme weight loss methods.</u> The interventionist and mental health program staff will be aware of the possibility that intervention subjects could use extreme methods to lose weight. Participants will be reminded regularly of the importance of safe weight loss. Those who have a sudden, marked weight reduction will be interviewed to determine if extreme measures have been taken. The intervention staff will be trained to detect evidence of extreme measures and will be given strategies for responding.

Nicotine replacement therapy and smoking cessation. We will educate participants about symptoms of nicotine withdrawal and teach coping strategies. NRT including in combination (patches and lozenges) is considered safe and risks are far less than those associated with tobacco use, even in

smokers with known cardiovascular disease and in those who continue smoking while using NRT.[185-192] Certain plasma levels of medications including olanzapine, clozapine and fluvoxamine may be higher after smoking cessation. We will discuss use of NRT with participants' primary care physicians and psychiatrists and monitor symptoms carefully at each weekly to bi-weekly intervention visit so that risks are minimized. We will communicate with providers regarding smoking cessation so that psychotropic medication dosage adjustment may be made if needed.

c. Plan for reporting unanticipated problems or study deviations.

Data and safety monitoring plan: Protection of research participants begins with the eligibility criteria, which are designed to exclude individuals with a serious medical condition that would preclude their ability to participate in a cardiovascular risk reduction program. During the study, clinical care will be provided by the participants' usual specialty mental health providers and the primary care physician, not by the study. Participants will be made aware of this delineation of responsibility. However, recognizing the opportunity for early detection of clinical problems and the small risk of study-related morbidity, we will perform periodic safety assessments. We will inquire about cardiovascular and musculoskeletal symptoms at 6-month intervals. Safety monitoring will consist of a symptom questionnaire and the Rose Angina questionnaire. Any positive responses to these questions will be reviewed by the safety officer; clinically significant results will lead to referral to the primary care physician. Information that comes to the attention of study personnel informally (e.g., through data collection or intervention activities at the center) may also lead to referral. Symptoms that will lead to referral include (but are not limited to) those that suggest cardiovascular disease (e.g., exertional chest pain, dyspnea, presyncope or syncope), uncontrolled hypertension, uncontrolled diabetes or complications of physical activity. Participants are also queried at these same timepoints about possible adverse events (defined below). Positive responses trigger an adverse event (AE) record, which is reviewed and classified as either gastrointestinal, cardiovascular, musculoskeletal, or other in nature. This information is then reported to the DSMB. Similar information reported by participants at other times (e.g., during intervention classes) is duly noted and followed up with as needed to assure participant safety. The following constitute adverse events (AEs): heart attack, stroke, transient ischemic attack, heart failure, coronary angioplasty or bypass surgery, angina pectoris, broken bone, torn ligament, and any other serious injury to the bone or muscle. Evidence of the occurrence of these events is based on participant self-report that a health care professional has diagnosed the condition. We will attempt to verify the diagnosis through contact with the primary care physician. Though not considered AEs for this study, we also track and report the incidence of hyperlipidemia, gallbladder disease, diabetes, cancer and hospitalization. All other outcomes that may be construed as being an adverse consequence of study participation, such as an injury while performing a study measurement, are documented, reviewed, and followed up on as needed by a study clinician.

d. Legal risks such as the risks that would be associated with breach of confidentiality.

Breach of confidentiality: All participant information will be considered confidential. This confidentiality will be assured through several mechanisms. For the study, each participant will be assigned an anonymous study ID, which will be used on all study forms. In addition, all study forms and paper records that contain participant information will be kept in secured, locked areas when not in use. Such materials, when in use, will be kept away from public scrutiny. Forms that need to be discarded will be destroyed. Moreover, access to all participant data and information will be restricted to authorized personnel. In the case of computerized study data, access will be password protected. When the study database is ready for analysis, it will not contain actual identities of participants. During active data collection, hard copies of data collection forms will be stored in locked areas with access only by authorized personnel. Finally, neither the mental health programs nor participants will be identified by name in any publications. Data will not be presented in such a way that the identity of individual participants can be inferred.

Similarly, for staff semi-structured interviews, all information will be considered confidential. This confidentiality

will be assured through several mechanisms. Transcripts will not contain names and will be kept in a secure location by Dr. Daumit. Audiotapes will be destroyed after transcription is completed. Individuals will not be identified by name in any publication. Mosaic leadership agrees that staff participation in the interview and any information shared will not affect staff employment or evaluation.

e. Financial risks to the participants.

None.

8. Benefits

a. Description of the probable benefits for the participant and for society.

Potential benefits to the study participants who receive the active intervention include improved health behaviors and improved cardiovascular risk factor profiles. The physical activity classes are available for active and control participants. Potential benefits to others include the possibility that the research will lead to the dissemination of effective interventions to decrease CVD risk in persons with serious mental illness.

There is no specific benefit to staff for participating in interviews. Information learned from their perspectives potentially will help disseminate health interventions in mental health organizations in the future.

9. Payment and Remuneration

a. Detail compensation for participants including possible total compensation, proposed bonus, and any proposed reductions or penalties for not completing the protocol.

At baseline and 6 months, for completing data collection activities, participants will receive 15 dollars. At 18 months, for completing data collection activities, participants will receive 25 dollars. This increase in payment as participants' time in the study progresses is to recognize their commitment to the study.

Staff will receive a 25 dollar gift card for completing a semi-structured interview.

10. Costs

a. Detail costs of study procedure(s) or drug (s) or substance(s) to participants and identify who will pay for them.

N/A

BIBLIOGRAPHY AND REFERENCES CITED

1. Health, United States, 2009: With special features on Medical Technology. Hyattsville, MD: National Center for Health Statistics; 2010.
2. Brown S. Excess mortality of schizophrenia. A meta-analysis. Br J Psychiatry 1997;171:502-8.
3. Daumit GL, Anthony CB, Ford DE, et al. Pattern of mortality in a sample of Maryland residents with severe mental illness. Psychiatry Res 2010;176:242-5.
4. Osby U, Correia N, Brandt L, Ekbom A, Sparen P. Mortality and causes of death in schizophrenia in Stockholm county, Sweden. Schizophr Res 2000;45:21-8.
5. Saha S, Chant D, McGrath J. A systematic review of mortality in schizophrenia: is the differential mortality gap worsening over time? Arch Gen Psychiatry 2007;64:1123-31.
6. Mueser KT, McGurk SR. Schizophrenia. Lancet 2004;363:2063-72.
7. Primm AB, Vasquez MJ, Mays RA, et al. The role of public health in addressing racial and ethnic disparities in mental health and mental illness. Prev Chronic Dis 2010;7:A20.
8. Lloyd-Jones DM, Hong Y, Labarthe D, et al. Defining and setting national goals for cardiovascular health promotion and disease reduction: the American Heart Association's strategic Impact Goal through 2020 and beyond. Circulation 2010;121:586-613.
9. Allison DB, Fontaine KR, Heo M, et al. The distribution of body mass index among individuals with and without schizophrenia. J Clin Psychiatry 1999;60:215-20.
10. Coodin S. Body mass index in persons with schizophrenia. Can J Psychiatry 2001;46:549-55.
11. Daumit GL, Clark JM, Steinwachs DM, Graham CM, Lehman A, Ford DE. Prevalence and correlates of obesity in a community sample of individuals with severe and persistent mental illness. J Nerv Ment Dis 2003;191:799-805.
12. McElroy SL. Correlates of Overweight and Obesit in 644 Patients with Bipolar Disorder. J Clin Psychiatry 2002;63:207-13.
13. Depression Guideline Panel: Depression in Primary Care, vol. 2. Rockville, MD: AHCPR; 1993. Report No.: 93-0551.
14. Allison DB, Mentore JL, Heo M, et al. Antipsychotic-induced weight gain: a comprehensive research synthesis. Am J Psychiatry 1999;156:1686-96.
15. Baptista T, Kin NM, Beaulieu S, de Baptista EA. Obesity and related metabolic abnormalities during antipsychotic drug administration: mechanisms, management and research perspectives. Pharmacopsychiatry 2002;35:205-19.
16. Macritchie KAN, Geddes JR, Scott J, Haslam DRS, Goodwin GM. Valproic acid, valproate and divalproex in the maintenance treatment of bipolar disorder (Cochrane Review). The Cochrane Library2002.
17. Sachs GS, Guille C. Weight gain associated with use of psychotropic medications. J Clin Psychiatry 1999;60 Suppl 21:16-9.
18. Lieberman JA, Stroup TS, McEvoy JP, et al. Effectiveness of antipsychotic drugs in patients with chronic schizophrenia. N Engl J Med 2005;353:1209-23.
19. Daumit GL, Goldberg RW, Anthony C, et al. Physical activity patterns in adults with severe mental illness. J Nerv Ment Dis 2005;193:641-6.
20. Jerome GJ, Young DR, Dalcin A, et al. Physical activity levels of persons with mental illness attending psychiatric rehabilitation programs. Schizophr Res 2009;108:252-7.
21. Amani R. Is dietary pattern of schizophrenia patients different from healthy subjects? BMC Psychiatry 2007;7:15.
22. McCreadie RG, Scottish Schizophrenia Lifestyle G. Diet, smoking and cardiovascular risk in people with schizophrenia: descriptive study. Br J Psychiatry 2003;183:534-9.
23. Strassnig M, Brar JS, Ganguli R. Nutritional assessment of patients with schizophrenia: a preliminary study. Schizophr Bull 2003;29:393-7.
24. Goff DC, Sullivan LM, McEvoy JP, et al. A comparison of ten-year cardiac risk estimates in schizophrenia patients from the CATIE study and matched controls. Schizophr Res 2005;80:45-53.
25. Holmberg SK, Kane C. Health and self-care practices of persons with schizophrenia. Psychiatr Serv 1999;50:827-9.
26. Ziedonis D, Hitsman B, Beckham JC, et al. Tobacco use and cessation in psychiatric disorders: National Institute of Mental Health report. Nicotine Tob Res 2008;10:1691-715.
27. Tidey JW, Rohsenow DJ, Kaplan GB, Swift RM. Cigarette smoking topography in smokers with schizophrenia and matched non-psychiatric controls. Drug and alcohol dependence 2005;80:259-65.
28. Williams JM, Ziedonis DM, Abanyie F, Steinberg ML, Foulds J, Benowitz NL. Increased nicotine and cotinine levels in smokers with schizophrenia and schizoaffective disorder is not a metabolic effect. Schizophrenia research 2005;79:323-35.
29. Goff DC, Henderson DC, Amico E. Cigarette smoking in schizophrenia: relationship to psychopathology and medication side effects. Am J Psychiatry 1992;149:1189-94.
30. Consensus Development Conference on Antipsychotic Drugs and Obesity and Diabetes. Diabetes Care 2004;27:596-58.
31. Bresee LC, Majumdar SR, Patten SB, Johnson JA. Prevalence of cardiovascular risk factors and disease in people with schizophrenia: A population-based study. Schizophr Res 2010.
32. Bushe C, Leonard B. Association between atypical antipsychotic agents and type 2 diabetes: review of prospective clinical data. Br J Psychiatry Suppl 2004;47:S87-93.
33. Dixon L, Weiden P, Delahanty J, et al. Prevalence and correlates of diabetes in national schizophrenia samples. Schizophr Bull 2000;26:903-12.
34. McEvoy JP, Meyer JM, Goff DC, et al. Prevalence of the metabolic syndrome in patients with schizophrenia: baseline results from the Clinical Antipsychotic Trials of Intervention Effectiveness (CATIE) schizophrenia trial and comparison with national estimates from NHANES III. Schizophr Res 2005;80:19-32.

35. Sokal J, Messias E, Dickerson F, et al. Co-morbidity of medical illnesses among adults with serious mental illness who are receiving community psychiatric services. Journal of Nervous and Mental Disease 2004;192:421-7.
36. Casey DE. Metabolic issues and cardiovascular disease in patients with psychiatric disorders. Am J Med 2005;118 Suppl 2:15S-22S.
37. Dwyer DS, Pinkofsky HB, Liu Y, Bradley RJ. Antipsychotic drugs affect glucose uptake and the expression of glucose transporters in PC12 cells. Prog Neuropsychopharmacol Biol Psychiatry 1999;23:69-80.
38. Haupt DW, Newcomer JW. Hyperglycemia and antipsychotic medications. J Clin Psychiatry 2001;62 Suppl 27:15-26; discussion 40-1.
39. Newcomer JW. Comparing the safety and efficacy of atypical antipsychotics in psychiatric patients with comorbid medical illnesses. J Clin Psychiatry 2009;70 Suppl 3:30-6.
40. Newcomer JW, Haupt DW, Fucetola R, et al. Abnormalities in Glucose Regulation during antipsychotic treatment of Schizophrenia. Arch Gen Psychiatry 2002;59:337-45.
41. Chobanian AV, Bakris GL, Black HR, et al. The Seventh Report of the Joint National Committee on Prevention, Detection, Evaluation, and Treatment of High Blood Pressure: the JNC 7 report. JAMA 2003;289:2560-72.
42. Koskinen J, Lohonen J, Koponen H, Isohanni M, Miettunen J. Prevalence of alcohol use disorders in schizophrenia--a systematic review and meta-analysis. Acta Psychiatr Scand 2009;120:85-96.
43. Newcomer JW. Medical risk in patients with bipolar disorder and schizophrenia. J Clin Psychiatry 2006;67 Suppl 9:25-30; discussion 6-42.
44. Dixon L, Postrado L, Delahanty J, Fischer PJ, Lehman A. The association of medical comorbidity in schizophrenia with poor physical and mental health. J Nerv Ment Dis 1999;187:496-502.
45. Casey DE. Dyslipidemia and atypical antipsychotic drugs. J Clin Psychiatry 2004;65 Suppl 18:27-35.
46. Daumit GL, Goff DC, Meyer JM, et al. Antipsychotic effects on estimated 10-year coronary heart disease risk in the CATIE schizophrenia study. Schizophr Res 2008;105:175-87.
47. L'Italien GJ, Casey DE, Kan HJ, Carson WH, Marcus RN. Comparison of metabolic syndrome incidence among schizophrenia patients treated with aripiprazole versus olanzapine or placebo. J Clin Psychiatry 2007;68:1510-6.
48. Meyer JM, Davis VG, McEvoy JP, et al. Impact of antipsychotic treatment on nonfasting triglycerides in the CATIE Schizophrenia Trial phase 1. Schizophr Res 2008;103:104-9.
49. Newcomer JW. Second-generation (atypical) antipsychotics and metabolic effects: a comprehensive literature review. CNS Drugs 2005;19 Suppl 1:1-93.
50. Sharpe JK, Hills AP. Anthropometry and adiposity in a group of people with chronic mental illness. Aust N Z J Psychiatry 1998;32:77-81.
51. Newcomer JW, Hennekens CH. Severe mental illness and risk of cardiovascular disease. JAMA 2007;298:1794-6.
52. Brar JS, Ganguli R, Pandina G, Turkoz I, Berry S, Mahmoud R. Effects of behavioral therapy on weight loss in overweight and obese patients with schizophrenia or schizoaffective disorder. Journal of Clinical Psychiatry 2005;66:205-12.
53. Evans S, Newton R, Higgins S. Nutritional intervention to prevent weight gain in patients commenced on olanzapine: a randomized controlled trial. Aust N Z J Psychiatry 2005;39:479-86.
54. Faulkner G, Soundy AA, Lloyd K. Schizophrenia and weight management: a systematic review of interventions to control weight. Acta Psychiatr Scand 2003;108:324-32.
55. Kwon JS, Choi JS, Bahk WM, et al. Weight management program for treatment-emergent weight gain in olanzapine-treated patients with schizophrenia or schizoaffective disorder: A 12-week randomized controlled clinical trial. J Clin Psychiatry 2006;67:547-53.
56. Littrell KH, Hilligoss NM, Kirshner CD, Petty RG, Johnson CG. The effects of an educational intervention on antipsychotic-induced weight gain. J Nurs Scholarsh 2003;35:237-41.
57. McKibbin CL, Patterson TL, Norman G, et al. A lifestyle intervention for older schizophrenia patients with diabetes mellitus: a randomized controlled trial. Schizophr Res 2006;86:36-44.
58. Weber M, Wyne K. A cognitive/behavioral group intervention for weight loss in patients treated with atypical antipsychotics. Schizophr Res 2006;83:95-101.
59. Daumit GL, Dickerson FB, Wang NY, et al. A behavioral weight-loss intervention in persons with serious mental illness. N Engl J Med 2013;368:1594-602.
60. Siru R, Hulse GK, Tait RJ. Assessing motivation to quit smoking in people with mental illness: a review. Addiction 2009;104:719-33.
61. Kreyenbuhl J, Buchanan RW, Dickerson FB, Dixon LB, Schizophrenia Patient Outcomes Research T. The Schizophrenia Patient Outcomes Research Team (PORT): updated treatment recommendations 2009. Schizophr Bull 2010;36:94-103.
62. Evins AE, Cather C, Deckersbach T, et al. A double-blind placebo-controlled trial of bupropion sustained-release for smoking cessation in schizophrenia. J Clin Psychopharmacol 2005;25:218-25.
63. Evins AE, Cather C, Rigotti NA, et al. Two-year follow-up of a smoking cessation trial in patients with schizophrenia: increased rates of smoking cessation and reduction. J Clin Psychiatry 2004;65:307-11; quiz 452-3.
64. Evins AE, Mays VK, Rigotti NA, Tisdale T, Cather C, Goff DC. A pilot trial of bupropion added to cognitive behavioral therapy for smoking cessation in schizophrenia. Nicotine Tob Res 2001;3:397-403.
65. Baker A, Richmond R, Haile M, et al. A randomized controlled trial of a smoking cessation intervention among people with a psychotic disorder. Am J Psychiatry 2006;163:1934-42.

66. Ziedonis DM, George TP. Schizophrenia and nicotine use: report of a pilot smoking cessation program and review of neurobiological and clinical issues. Schizophrenia bulletin 1997;23:247-54.
67. Weiner E, Ball MP, Summerfelt A, Gold J, Buchanan RW. Effects of sustained-release bupropion and supportive group therapy on cigarette consumption in patients with schizophrenia. Am J Psychiatry 2001;158:635-7.
68. George TP, Vessicchio JC, Termine A, et al. A placebo controlled trial of bupropion for smoking cessation in schizophrenia. Biol Psychiatry 2002;52:53-61.
69. George TP, Ziedonis DM, Feingold A, et al. Nicotine transdermal patch and atypical antipsychotic medications for smoking cessation in schizophrenia. Am J Psychiatry 2000;157:1835-42.
70. Casagrande SS, Jerome GJ, Dalcin AT, et al. Randomized trial of achieving healthy lifestyles in psychiatric rehabilitation: the ACHIEVE trial. BMC Psychiatry 2010;10:108.
71. Tsoi DT, Porwal M, Webster AC. Interventions for smoking cessation and reduction in individuals with schizophrenia. Cochrane Database Syst Rev 2013;2:CD007253.
72. Dutra SJ, Stoeckel LE, Carlini SV, Pizzagalli DA, Evins AE. Varenicline as a smoking cessation aid in schizophrenia: effects on smoking behavior and reward sensitivity. Psychopharmacology (Berl) 2011.
73. Williams JM, Anthenelli RM, Morris CD, et al. A randomized, double-blind, placebo-controlled study evaluating the safety and efficacy of varenicline for smoking cessation in patients with schizophrenia or schizoaffective disorder. J Clin Psychiatry 2012;73:654-60.
74. Weiner E, Buchholz A, Coffay A, et al. Varenicline for smoking cessation in people with schizophrenia: a double blind randomized pilot study. Schizophr Res 2011;129:94-5.
75. Pachas GN, Cather C, Pratt SA, et al. Varenicline for Smoking Cessation in Schizophrenia: Safety and Effectiveness in a 12-Week, Open-Label Trial. J Dual Diagn 2012;8:117-25.
76. Evins AE. Reassessing the Safety of Varenicline. Am J Psychiatry 2013;170.
77. Gibbons RD, Mann JJ. Varenicline, Smoking Cessation, and Neuropsychiatric Adverse Events. Am J Psychiatry 2013.
78. Wu BS, Weinberger AH, Mancuso E, et al. A Preliminary Feasibility Study of Varenicline for Smoking Cessation in Bipolar Disorder. J Dual Diagn 2012;8:131-2.
79. George TP, Vessicchio JC, Termine A, et al. A placebo controlled trial of bupropion for smoking cessation in schizophrenia. Biological psychiatry 2002;52:53-61.
80. Heffner JL, Anthenelli RM, Delbello MP, Stahl L, Strakowski SM. Mood management and nicotine patch for smoking cessation in adults with bipolar disorder. Nicotine Tob Res 2012;15:1805-6.
81. Weinberger AH, Vessicchio JC, Sacco KA, Creeden CL, Chengappa KN, George TP. A preliminary study of sustained-release bupropion for smoking cessation in bipolar disorder. J Clin Psychopharmacol 2008;28:584-7.
82. Evins AE. Extended Duration Pharmacotherapy for Sustained Tobacco Abstinence in People with Serious Mental Illness In Symposium: $\alpha_4\beta_2$-Nicotinic Acetylcholine Receptors In Schizophrenia: Implications for Comorbid Tobacco Smoking and Therapeutics. Society for Research on Nicotine and Tobacco 2013; Boston, MA.
83. Chengappa KN. Varenicline for Initiating Smoking Cessation in Persons with Bipolar Disorder A Random-Assignment, Placebo-Controlled, Parallel Group Clinical Trial (PowerPoint). American Psychiatric Assoication 166th Annual Meeting San Franciso, CA.(5/18/2013).
84. Evins AE, Cather C, Pratt SA, et al. Maintenance treatment with varenicline for smoking cessation in patients with schizophrenia and bipolar disorder: a randomized clinical trial. JAMA 2014;311:145-54.
85. Anthenelli RM, Benowitz NL, West R, et al. Neuropsychiatric safety and efficacy of varenicline, bupropion, and nicotine patch in smokers with and without psychiatric disorders (EAGLES): a double-blind, randomised, placebo-controlled clinical trial. The Lancet 2016;387:2507-20.
86. Renders C, Valk G, Griffin S, Wagner E, Thm van Eijk J, Assendelft W. Interventions to improve the management of diabetes mellitus in primary care, outpatient and community settings. Cochrane database of systematic reviews 2000:CD001481.
87. Glynn L, Murphy A, Smith S, Schroeder K, Fahey T. Interventions used to improve control of blood pressure in patients with hypertension. Cochrane Database of Systematic Reviews 2010;3:CD005182.
88. Schedlbauer A, Davies P, Fahey T. Interventions to improve adherence to lipid lowering medication. Cochrane Database Syst Rev 2010:CD004371.
89. Haskell WL, Berra K, Arias E, et al. Multifactor cardiovascular disease risk reduction in medically underserved, high-risk patients. The American journal of cardiology 2006;98:1472-9.
90. Haskell WL, Alderman EL, Fair JM, et al. Effects of intensive multiple risk factor reduction on coronary atherosclerosis and clinical cardiac events in men and women with coronary artery disease. The Stanford Coronary Risk Intervention Project (SCRIP). Circulation 1994;89:975-90.
91. Becker DM, Yanek LR, Johnson WR, Jr., et al. Impact of a community-based multiple risk factor intervention on cardiovascular risk in black families with a history of premature coronary disease. Circulation 2005;111:1298-304.
92. Katon WJ, Lin EH, Von Korff M, et al. Collaborative care for patients with depression and chronic illnesses. N Engl J Med 2010;363:2611-20.
93. Ogedegbe G, Chaplin W, Schoenthaler A, et al. A practice-based trial of motivational interviewing and adherence in hypertensive African Americans. Am J Hypertens 2008;21:1137-43.
94. Schedlbauer A, Davies P, Fahey T. Interventions to improve adherence to lipid lowering medication. Cochrane Database Syst Rev 2010:CD004371.

95. Druss BG, Rohrbaugh RM, Levinson CM, Rosenheck RA. Integrated medical care for patients with serious psychiatric illness: a randomized trial. Arch Gen Psychiatry 2001;58:861-8.
96. Druss BG, von Esenwein SA. Improving general medical care for persons with mental and addictive disorders: systematic review. Gen Hosp Psychiatry 2006;28:145-53.
97. Druss BG, von Esenwein SA, Compton MT, Rask KJ, Zhao L, Parker RM. A randomized trial of medical care management for community mental health settings: the Primary Care Access, Referral, and Evaluation (PCARE) study. Am J Psychiatry 2010;167:151-9.
98. Umbricht-Schneiter A, Ginn DH, Pabst KM, Bigelow GE. Providing medical care to methadone clinic patients: referral vs on-site care. Am J Public Health 1994;84:207-10.
99. Breslau J, Leckman-Westin E, Han B, et al. Impact of a mental health based primary care program on emergency department visits and inpatient stays. General Hospital Psychiatry 2018;52:8-13.
100. Breslau J, Leckman-Westin E, Yu H, et al. Impact of a Mental Health Based Primary Care Program on Quality of Physical Health Care. Administration and Policy in Mental Health and Mental Health Services Research 2018;45:276-85.
101. Druss BG, von Esenwein SA, Glick GE, et al. Randomized Trial of an Integrated Behavioral Health Home: The Health Outcomes Management and Evaluation (HOME) Study. Am J Psychiatry 2017;174:246-55.
102. Gilmer TP, Henwood BF, Goode M, Sarkin AJ, Innes-Gomberg D. Implementation of Integrated Health Homes and Health Outcomes for Persons With Serious Mental Illness in Los Angeles County. Psychiatr Serv 2016;67:1062-7.
103. Krupski A, West, II, Scharf DM, et al. Integrating Primary Care Into Community Mental Health Centers: Impact on Utilization and Costs of Health Care. Psychiatr Serv 2016;67:1233-9.
104. Tepper MC, Cohen AM, Progovac AM, et al. Mind the Gap: Developing an Integrated Behavioral Health Home to Address Health Disparities in Serious Mental Illness. Psychiatr Serv 2017;68:1217-24.
105. Heidenreich PA, Trogdon JG, Khavjou OA, et al. Forecasting the future of cardiovascular disease in the United States: a policy statement from the American Heart Association. Circulation 2011;123:933-44.
106. Heart Disease and Stroke Statistics — 2010 Update2010.
107. Incidence and Prevalence: 2006 Chart Book on Cardiovascular and Lung Diseases. Bethesda, MD2006.
108. American Heart Association and National Heart L, and Blood Institute. Exercise and Your Heart: A guide to physical activity AHA; 2001.
109. Daumit GL, Dalcin AT, Jerome GJ, et al. A behavioral weight loss intervention for persons with serious mental illness in psychiatric rehabilitation centers. International Journal of Obesity 2010:1–10.
110. Daumit G, Appel L, Leatherman E, et al. Randomized Trial of Peer-Supported Physical Activity for Persons with Severe Mental Illness in Community Psychiatry. Journal of General Internal Medicine 2010;25:378-9.
111. Gary TL, Batts-Turner M, Yeh HC, et al. The effects of a nurse case manager and a community health worker team on diabetic control, emergency department visits, and hospitalizations among urban African Americans with type 2 diabetes mellitus: a randomized controlled trial. Arch Intern Med 2009;169:1788-94.
112. Campbell MK, Resnicow K, Carr C, Wang T, Williams A. Process evaluation of an effective church-based diet intervention: Body & Soul. Health Educ Behav 2007;34:864-80.
113. Hill-Briggs F, Batts-Turner M, Gary TL, et al. Training community health workers as diabetes educators for urban African Americans: value added using participatory methods. Prog Community Health Partnersh 2007;1:185-94.
114. Hyman DJ, Pavlik VN, Taylor WC, Goodrick GK, Moye L. Simultaneous vs sequential counseling for multiple behavior change. Arch Intern Med 2007;167:1152-8.
115. Standards of medical care in diabetes--2011. Diabetes care 2011;34 Suppl 1:S11-61.
116. Third Report of the National Cholesterol Education Program (NCEP) Expert Panel on Detection, Evaluation, and Treatment of High Blood Cholesterol in Adults (Adult Treatment Panel III) final report. Circulation 2002;106:3143-421.
117. Appel LJ, Brands MW, Daniels SR, Karanja N, Elmer PJ, Sacks FM. Dietary approaches to prevent and treat hypertension: a scientific statement from the American Heart Association. Hypertension 2006;47:296-308.
118. Evins AE. Reassessing the safety of varenicline. Am J Psychiatry 2013;170:1385-7.
119. Stone NJ, Robinson JG, Lichtenstein AH, et al. 2013 ACC/AHA guideline on the treatment of blood cholesterol to reduce atherosclerotic cardiovascular risk in adults: a report of the American College of Cardiology/American Heart Association Task Force on Practice Guidelines. Circulation 2014;129:S1-45.
120. Treating tobacco use and dependence: 2008 update U.S. Public Health Service Clinical Practice Guideline executive summary. Respir Care 2008;53:1217-22.
121. James PA, Oparil S, Carter BL, et al. 2014 evidence-based guideline for the management of high blood pressure in adults: report from the panel members appointed to the Eighth Joint National Committee (JNC 8). Jama 2014;311:507-20.
122. Ebbert JO, Hughes JR, West RJ, et al. Effect of Varenicline on Smoking Cessation Through Smoking Reduction: A Randomized Clinical Trial. Jama 2015;313:687-94.
123. American Diabetes Association. Standards of medical care in diabetes—2014. Diabetes Care 2014;37:S14-S80.
124. Buchanan RW, Kreyenbuhl J, Kelly DL, et al. The 2009 schizophrenia PORT psychopharmacological treatment recommendations and summary statements. Schizophr Bull 2010;36:71-93.
125. Rose G, McCartney P, Reid DD. Self-administration of a questionnaire on chest pain and intermittent claudication. Br J Prev Soc Med 1977;31:42-8.
126. Cacciola JS, Alterman AI, McLellan AT, Lin YT, Lynch KG. Initial evidence for the reliability and validity of a "Lite" version of the Addiction Severity Index. Drug Alcohol Depend 2007;87:297-302.

127. White WB, Anwar YA. Evaluation of the overall efficacy of the Omron office digital blood pressure HEM-907 monitor in adults. Blood Press Monit 2001;6:107-10.
128. Vollmer WM, Appel LJ, Svetkey LP, et al. Comparing office-based and ambulatory blood pressure monitoring in clinical trials. J Hum Hypertens 2005;19:77-82.
129. Laboratories ATSCoPSfCPF. ATS statement: guidelines for the six-minute walk test. Am J Respir Crit Care Med 2002;166:111-7.
130. Grant S, Corbett K, Amjad AM, Wilson J, Aitchison T. A comparison of methods of predicting maximum oxygen uptake. Br J Sports Med 1995;29:147-52.
131. Johnson DJ, Oliver RA, Terry JW. Regression equation for prediction of performance in the twelve minute run walk test. J Sports Med Phys Fitness 1979;19:165-70.
132. Friedewald WT, Levy RI, Fredrickson DS. Estimation of the concentration of low-density lipoprotein cholesterol in plasma, without use of the preparative ultracentrifuge. Clin Chem 1972;18:499-502.
133. Bingham SA, Gill C, Welch A, et al. Validation of dietary assessment methods in the UK arm of EPIC using weighed records, and 24-hour urinary nitrogen and potassium and serum vitamin C and carotenoids as biomarkers. Int J Epidemiol 1997;26 Suppl 1:S137-51.
134. Martini MC, Campbell DR, Gross MD, Grandits GA, Potter JD, Slavin JL. Plasma carotenoids as biomarkers of vegetable intake: the University of Minnesota Cancer Prevention Research Unit Feeding Studies. Cancer Epidemiol Biomarkers Prev 1995;4:491-6.
135. van Kappel AL, Steghens JP, Zeleniuch-Jacquotte A, Chajes V, Toniolo P, Riboli E. Serum carotenoids as biomarkers of fruit and vegetable consumption in the New York Women's Health Study. Public Health Nutr 2001;4:829-35.
136. Jarvis MJ, Russell MA, Saloojee Y. Expired air carbon monoxide: a simple breath test of tobacco smoke intake. Br Med J 1980;281:484-5.
137. Verification SSoB. Biochemical verification of tobacco use and cessation. Nicotine Tob Res 2002;4:149-59.
138. Acosta M, Buchhalter A, Breland A, Hamilton D, Eissenberg T. Urine cotinine as an index of smoking status in smokers during 96-hr abstinence: comparison between gas chromatography/mass spectrometry and immunoassay test strips. Nicotine Tob Res 2004;6:615-20.
139. Block G, Gillespie C, Rosenbaum EH, Jenson C. A rapid food screener to assess fat and fruit and vegetable intake. Am J Prev Med 2000;18:284-8.
140. Heatherton TF, Kozlowski LT, Frecker RC, Fagerstrom KO. The Fagerstrom Test for Nicotine Dependence: a revision of the Fagerstrom Tolerance Questionnaire. Br J Addict 1991;86:1119-27.
141. Hughes JR, Keely JP, Niaura RS, Ossip-Klein DJ, Richmond RL, Swan GE. Measures of abstinence in clinical trials: issues and recommendations. Nicotine & tobacco research : official journal of the Society for Research on Nicotine and Tobacco 2003;5:13-25.
142. Karelitz JL, Michael VC, Perkins KA. Analysis of Agreement between Expired-Air Carbon Monoxide Monitors. J Smok Cessat 2017;12:105-12.
143. Salyers MP, Bosworth HB, Swanson JW, Lamb-Pagone J, Osher FC. Reliability and validity of the SF-12 health survey among people with severe mental illness. Medical Care 2000;38:1141-50.
144. Bobes J, Garcia-Portilla P, Saiz PA, Bascaran T, Bousono M. Quality of life measures in schizophrenia. Eur Psychiatry 2005;20 Suppl 3:S313-7.
145. Fryback DG, Dunham NC, Palta M, et al. US norms for six generic health-related quality-of-life indexes from the National Health Measurement study. Med Care 2007;45:1162-70.
146. Hayhurst H, Palmer S, Abbott R, Johnson T, Scott J. Measuring health-related quality of life in bipolar disorder: relationship of the EuroQol (EQ-5D) to condition-specific measures. Qual Life Res 2006;15:1271-80.
147. Morisky DE, Ang A, Krousel-Wood M, Ward HJ. Predictive validity of a medication adherence measure in an outpatient setting. J Clin Hypertens (Greenwich) 2008;10:348-54.
148. Radloff L. The CES-D Scale: A self-report depression scale for research in the general population. Applied Psyhcological Measurement 1977;1:385-401.
149. Weissman MM, Sholomskas D, Pottenger M, Prusoff BA, Locke BZ. Assessing depressive symptoms in five psychiatric populations: a validation study. Am J Epidemiol 1977;106:203-14.
150. Eisen SV, Normand SL, Belanger AJ, Spiro A, 3rd, Esch D. The Revised Behavior and Symptom Identification Scale (BASIS-R): reliability and validity. Med Care 2004;42:1230-41.
151. Eisen SV, Ranganathan G, Seal P, Spiro A, 3rd. Measuring clinically meaningful change following mental health treatment. J Behav Health Serv Res 2007;34:272-89.
152. Niv N, Cohen AN, Mintz J, Ventura J, Young AS. The validity of using patient self-report to assess psychotic symptoms in schizophrenia. Schizophr Res 2007;90:245-50.
153. Sallis JF, Pinski RB, Grossman RM, Patterson TL, Nader PR. The development of self efficacy scales for health-related diet and exercise behaviors. Health Education Research 1988;3:283-92.
154. Sherbourne CD, Stewart AL. The MOS social support survey. Soc Sci Med 1991;32:705-14.
155. Celio AA, Wilfley DE, Crow SJ, Mitchell J, Walsh BT. A comparison of the binge eating scale, questionnaire for eating and weight patterns-revised, and eating disorder examination questionnaire with instructions with the eating disorder examination in the assessment of binge eating disorder and its symptoms. Int J Eat Disord 2004;36:434-44.

156. Mujahid MS, Diez Roux AV, Morenoff JD, Raghunathan T. Assessing the measurement properties of neighborhood scales: from psychometrics to ecometrics. American journal of epidemiology 2007;165:858-67.
157. Buysse D, Reynolds C, Monk T, Berman S, Kupfer D. The Pittsburgh Sleep Quality Index (PSQI): A new instrument for psychiatric research and practice. Psychiatry Research 1989;28:193-213.
158. Casagrande SS, Dalcin A, McCarron P, et al. A Nutritional Intervention to Reduce the Calorie Content of Meals Served at Psychiatric Rehabilitation Programs. Community Ment Health J 2011.
159. D'Agostino RB, Ramachandran SV, Pencina MJ, et al. General cardiovascular risk profile for use in primary care: The Framingham Heart Study. Circulation 2008;17:743-53.
160. Wilson PW, D'Agostino RB, Levy D, Belanger AM, Silbershatz H, Kannel WB. Prediction of coronary heart disease using risk factor categories. Circulation 1998;97:1837-47.
161. Randomised controlled trial evaluating cardiovascular screening and intervention in general practice: principal results of British family heart study. Family Heart Study Group. BMJ 1994;308:313-20.
162. Appel LJ, Sacks FM, Carey VJ, et al. Effects of protein, monounsaturated fat, and carbohydrate intake on blood pressure and serum lipids: results of the OmniHeart randomized trial. Jama 2005;294:2455-64.
163. Maruthur NM, Wang NY, Appel LJ. Lifestyle interventions reduce coronary heart disease risk: results from the PREMIER Trial. Circulation 2009;119:2026-31.
164. Willaing I, Ladelund S, Jorgensen T, Simonsen T, Nielsen LM. Nutritional counselling in primary health care: a randomized comparison of an intervention by general practitioner or dietician. European journal of cardiovascular prevention and rehabilitation : official journal of the European Society of Cardiology, Working Groups on Epidemiology & Prevention and Cardiac Rehabilitation and Exercise Physiology 2004;11:513-20.
165. Harris PA, Taylor R, Thielke R, Payne J, Gonzalez N, Conde JG. Research electronic data capture (REDCap)- A metadata-and workflow process for providing translational research informatics support. J Biomed Infor 2009;42:377-81.
166. Rosner B. Fundamentals of biostatistics: Volume 1 6th ed ed. Belmont, CA: Duxbury Press; 2006.
167. Piantadosi S. Clinical Trials: A Methodologic Perspective. Second ed. Baltimore, MD: Wiley Interscience; 2005.
168. Little R, Rubin D. Statistical Analysis with Missing Data, Second Edition: Wiley and Sons; 2002.
169. Redmon JB, Bertoni AG, Connelly S, et al. Effect of the look AHEAD study intervention on medication use and related cost to treat cardiovascular disease risk factors in individuals with type 2 diabetes. Diabetes Care 2010;33:1153-8.
170. Muntner P, Joyce C, Holt E, et al. Defining the minimal detectable change in scores on the eight-item Morisky Medication Adherence Scale. Ann Pharmacother 2011;45:569-75.
171. Krousel-Wood M, Joyce C, Holt E, et al. Predictors of decline in medication adherence: results from the cohort study of medication adherence among older adults. Hypertension 2011;58:804-10.
172. Kraemer HC, Wilson GT, Fairburn CG, Agras WS. Mediators and moderators of treatment effects in randomized clinical trials. Arch Gen Psychiatry 2002;59:877-83.
173. Gold M, Siegel J, Russell L, Weinstein M. Cost-Effectiveness in Health and Medicine. New York: Oxford University Press; 1996.
174. Briggs AH, O'Brien BH, Blackhouse G. Thinking outside the box: recent advances in the analysis and presentation of uncertainty in cost-effectiveness studies. Annu Rev Public Health 2002;23:377-401.
175. Glaser BG. The Constant Comparative Method of Qualitative-Analysis. Social Problems 1965;12:436-45.
176. Bove AA, Santamore WP, Homko C, et al. Reducing cardiovascular disease risk in medically underserved urban and rural communities. Am Heart J 2011;161:351-9.
177. Ma J, Berra K, Haskell WL, et al. Case management to reduce risk of cardiovascular disease in a county health care system. Arch Intern Med 2009;169:1988-95.
178. Appel L, Champagne C, Harsha D, et al. Effects of Comprehensive Lifestyle Modification on Blood Pressure Control: Main results of the PREMIER Clinical Trial. Writing Group of the PREMIER Collaborative Research Group. JAMA 2003;289:2083-93.
179. Pi-Sunyer X, Blackburn G, Brancati FL, et al. Reduction in weight and cardiovascular disease risk factors in individuals with type 2 diabetes: one-year results of the look AHEAD trial. Diabetes care 2007;30:1374-83.
180. Hiss RG, Armbruster BA, Gillard ML, McClure LA. Nurse care manager collaboration with community-based physicians providing diabetes care: a randomized controlled trial. Diabetes Educ 2007;33:493-502.
181. Barr Taylor C, Miller NH, Reilly KR, et al. Evaluation of a nurse-care management system to improve outcomes in patients with complicated diabetes. Diabetes Care 2003;26:1058-63.
182. Spencer MS, Rosland AM, Kieffer EC, et al. Effectiveness of a Community Health Worker Intervention Among African American and Latino Adults With Type 2 Diabetes: A Randomized Controlled Trial. Am J Public Health 2011.
183. Welch G, Garb J, Zagarins S, Lendel I, Gabbay RA. Nurse diabetes case management interventions and blood glucose control: results of a meta-analysis. Diabetes Res Clin Pract 2010;88:1-6.
184. ACSM. ACSM's guidelines for exercise testing and prescription/American College of Sports Medicine. Philadelphia: Lippincott Williams & Wilkins; 2000.
185. Benowitz NL, Gourlay SG. Cardiovascular toxicity of nicotine: implications for nicotine replacement therapy. J Am Coll Cardiol 1997;29:1422-31.
186. Dale LC, Hurt RD, Offord KP, Lawson GM, Croghan IT, Schroeder DR. High-dose nicotine patch therapy. Percentage of replacement and smoking cessation. JAMA 1995;274:1353-8.
187. Heishman SJ, Balfour DJ, Benowitz NL, Hatsukami DK, Lindstrom JM, Ockene JK. Society for Research on Nicotine and Tobacco. Addiction 1997;92:615-33.

188. Jorenby DE, Smith SS, Fiore MC, et al. Varying nicotine patch dose and type of smoking cessation counseling. JAMA 1995;274:1347-52.
189. Zevin S, Jacob P, 3rd, Benowitz NL. Dose-related cardiovascular and endocrine effects of transdermal nicotine. Clin Pharmacol Ther 1998;64:87-95.
190. Gourlay SG, Benowitz NL. The benefits of stopping smoking and the role of nicotine replacement therapy in older patients. Drugs Aging 1996;9:8-23.
191. Rose JE, Behm FM, Westman EC, Kukovich P. Precessation treatment with nicotine skin patch facilitates smoking cessation. Nicotine Tob Res 2006;8:89-101.
192. Rose JE, Herskovic JE, Behm FM, Westman EC. Precessation treatment with nicotine patch significantly increases abstinence rates relative to conventional treatment. Nicotine Tob Res 2009;11:1067-75.
