# Supplementary material for: Effect of a Comprehensive Cardiovascular Risk Reduction Intervention in Persons With Serious Mental Illness: A Randomized Clinical Trial
Source: JAMA Netw Open. 2020 Jun 12;3(6):e207247. doi: 10.1001/jamanetworkopen.2020.7247 (PMC7293000; doi:10.1001/jamanetworkopen.2020.7247)
Supplement: Supplement 2. — eAppendix. Synopsis of Intervention eTable 1. Trial Enrollment Criteria eTable 2. Global Framingham Risk Score by Randomized Group at Each Visit (Cross-Sectional) and Model-Based Percent Longitudinal Change of Global Framingham Risk Score Over Time eFigure 1. Percent Change in ACC/AHA Risk Score Over Time According to Study Group eTable 3. ACC/AHA Risk Score by Randomized Group at Each Visit and Model-Based Percent Longitudinal Change of ACC/AHA Risk Score Over Time eFigure 2. Model-Based Percent Change in Global Framingham Risk Score by Subgroups of Sex, Race, Cardiovascular Risk at Baseline, and Psychiatric Diagnosis eTable 4. Overnight Hospitalizations, Deaths and Medical Events for Intervention and Control Participants [file jamanetwopen-3-e207247-s002.pdf]

Supplementary Online Content

Daumit GL, Dalcin AT, Dickerson FB, et al. Effect of a comprehensive cardiovascular risk reduction intervention in persons with serious mental illness: a randomized clinical trial. *JAMA Netw Open*. 2020;3(6):e207247. doi:10.1001/jamanetworkopen.2020.7247

eAppendix. Synopsis of Intervention
eTable 1. Trial Enrollment Criteria
eTable 2. Global Framingham Risk Score by Randomized Group at Each Visit (Cross-Sectional) and Model-Based Percent Longitudinal Change of Global Framingham Risk Score Over Time
eFigure 1. Percent Change in ACC/AHA Risk Score Over Time According to Study Group
eTable 3. ACC/AHA Risk Score by Randomized Group at Each Visit and Model-Based Percent Longitudinal Change of ACC/AHA Risk Score Over Time
eFigure 2. Model-Based Percent Change in Global Framingham Risk Score by Subgroups of Sex, Race, Cardiovascular Risk at Baseline, and Psychiatric Diagnosis
eTable 4. Overnight Hospitalizations, Deaths and Medical Events for Intervention and Control Participants

This supplementary material has been provided by the authors to give readers additional information about their work.

© 2020 Daumit GL et al. *JAMA Netw Open.*

eAppendix: Synopsis of Intervention

IDEAL Health Coaching Tracks with Behavioral Counseling Session Topics

SMOKERS: TRACK 1

Track 1a

(Smoking Cessation)

Session 1. Introduction to intervention

Session 2. Review CVD Risk Factor Medication Adherence

Session 3. Getting to Know your Physical Activity and Dietary Habits

Sessions 4 – 11. Smoking Cessation sessions

Session 12. Relapse Prevention

Session 13. Dietary

Session 14. Dietary

Session 15. Exercise

*Repeat dietary and exercise sessions and include relapse prevention discussion each time.

Track 1b

(Motivational Enhancement)

Session 1. Introduction to intervention

Session 2. Review CVD Risk Factor Medication Adherence

Session 3. Getting to Your Physical Activity and Dietary Habits

Sessions 4 – 7. Motivational Enhancement sessions

Session 8. If participant is willing to set a quit date, move to Track 1a.

If participant is not willing to set a quit date, repeat 4 motivational enhancement sessions and re-assess willingness to set a quit date.

After going through the motivational enhancement curriculum twice, the participant will move to Track 1a if they want to set a quit date. The participant will move to Track 1c if they do not want to set a quit date.

Track 1c

(Monthly Motivational Enhancement)

Session 1. Introduction to intervention

Session 2. Review CVD Risk Factor Medication Adherence

Session 3. Getting to Know your Physical Activity and Dietary Habits

Session 4. Monthly Motivational Enhancement Session

Session 5. Dietary

Session 6. Dietary

Session 7. Exercise

Session 8. Monthly Motivational Enhancement session

Repeat sessions 5-8 until participant expresses either:
-An interest in quitting smoking within 30 days (Track 1a)
-A willingness to talk about their smoking and/or reduce their smoking (Track 1b)

NON-SMOKERS

Track 2

Session 1. Introduction to intervention

Session 2. Review CVD Risk Factor Medication Adherence

Session 3. Getting to Know your Physical Activity and Dietary Habits

Session 4. Dietary

Session 5. Dietary

Session 6. Dietary

Session 7. Exercise

Session 8. Dietary

Session 9. Dietary

Session 10. Dietary

Session 11. Exercise

*Repeat dietary and exercise sessions

© 2020 Daumit GL et al. *JAMA Netw Open.*

Sample Health Coach Behavioral Counseling Session Flow

1	Set the stage and establish rapport<br>Welcome participant to session and review agenda	1-2 min
2	Collect intervention data<br>Weight<br>For smokers, smokelyzer depending on readiness to quit	1-2 min
3	Review content and goal from previous session<br>Brief review of material covered during previous session<br>Discuss goal, successes and challenges, including exercise. Highlight successes.	3-5 min
4	Discuss session topic<br>Smoking, dietary and/or exercise focus	3-5 min
5	Set goal and action plan*<br>Develop a specific and attainable behavioral goal and strategize ways for participant to be successful (what they need to do to accomplish their goal)	3-5 min
6	Summarize<br>Brief summary of what was discussed during session and remind participant of their goal	1 min
7	Schedule next session<br>Schedule next session and thank participant for their time	1 min

Encourage participant to keep the same behavioral goal until the goal is achieved.

Nurse Cardiovascular Risk Factor Education Session Topics

Diabetes Education Sessions

- Understanding Your Diabetes
- Medication management/Sick Day
- Monitoring blood sugar
- Eye care/Foot Care
- Exercise and Diabetes

Hypertension Education Sessions

- Understanding Hypertension
- Medication Adherence

© 2020 Daumit GL et al. *JAMA Netw Open.*

Intervention Adaptations and Strategies for Persons with Serious Mental Illness

Intervention Component	Adaptations and Strategies for Persons with Serious Mental Illness
Health Coach Sessions	-Meet frequently<br>-Concentrate on key modifiable dietary behaviors<br>-Allow for individualized cognitive tailoring as needed<br>-Address risk factors either sequentially or simultaneously based on readiness, attention and psychiatric symptoms<br>-Guide participants toward a personalized high impact behavior
Environmental Prompts	-Refrigerator magnets, preprinted grocery lists, watches, water bottle, measuring cups, portion plates, lunch bags<br>-Card with high impact individualized behavioral goal(s)
Contingency Management	-Participation rewarded with points that could be traded for varying levels of gifts relative to the number of sessions attended and decreasing CO_2 measurements
Self-monitoring	-Simplified tools - pack wraps for smokers, dietary tracker, pedometers
Nurse Sessions	-Repetition, simplified medical information, advocate for participants with physicians while honoring their autonomy, engage social supports and coordinate with mental health program staff to reinforce goals

Care Coordination/Care Management Approach by Risk Factor

Risk Factor	Care Coordination/Management Approach
Diabetes	-Regularly monitor blood glucose<br>-Communicate with physician about medications<br>-Coordinate with mental health program staff and caregivers around medication adherence, supply procurement, and appropriate diet
Dyslipidemia	-Advocate for and facilitate routine lipid monitoring<br>-Communicate with PCPs around guideline-concordant treatment with lipid lowering medications
Hypertension	-Routinely measure blood pressure<br>-Communicate with PCPs around guideline-concordant treatment with antihypertensive medications
Overweight/Obesity	-Emphasize role of weight loss for CVD risk management
Unhealthy diet	-Emphasize role of diet for CVD risk management
Physical inactivity	-Emphasize role of exercise for CVD risk management
Smoking	-Communicate with physician about pharmacotherapy<br>-Coordinate with mental health program staff and caregivers to support quitting

© 2020 Daumit GL et al. *JAMA Netw Open.*

Dietary Discussion Topics by Risk Factor

Diabetes
- Avoid Sugar Drinks
- Avoid Sweets
- Eat Vegetables and Whole Fruits Instead of Unhealthy Foods
- Eat Smart Portions

Blood Pressure
- Avoid Salty/Greasy Foods
- Avoid Processed Foods
- Eat Vegetables and Whole Fruits Instead of Unhealthy Foods
- Eat Smart Portions

Cholesterol
- Avoid Processed Foods
- Avoid Salty/Greasy Foods
- Avoid Sweets
- Eat Vegetables and Whole Fruits Instead of Unhealthy Foods
- Eat Smart Portions

Weight
- Avoid Sugar Drinks
- Avoid Sweets
- Avoid Salty/Greasy Foods
- Avoid Processed Foods
- Eat Vegetables and Whole Fruits Instead of Unhealthy Foods
- Eat Smart Portions

Diabetes and Blood Pressure
- Avoid Sugar Drinks
- Avoid Sweets
- Avoid Salty/Greasy Foods
- Avoid Processed Foods
- Eat Vegetables and Whole Fruits Instead of Unhealthy Foods
- Eat Smart Portions

Diabetes and Cholesterol
- Avoid Processed Foods
- Avoid Salty/Greasy Foods
- Avoid Sweets
- Avoid Sugar Drinks
- Eat Vegetables and Whole Fruits Instead of Unhealthy Foods
- Eat Smart Portions

Blood Pressure and Cholesterol
- Avoid Processed Foods
- Avoid Salty/Greasy Foods
- Avoid Sweets
- Eat Vegetables and Whole Fruits Instead of Unhealthy Foods
- Eat Smart Portions

© 2020 Daumit GL et al. *JAMA Netw Open.*

Diabetes, Blood Pressure, and Cholesterol

- Avoid Sugar Drinks
- Avoid Sweets
- Avoid Salty/Greasy Foods
- Avoid Processed Foods
- Eat Vegetables and Whole Fruits Instead of Unhealthy Foods
- Eat Smart Portions

© 2020 Daumit GL et al. *JAMA Netw Open.*

Dietary Options Tool

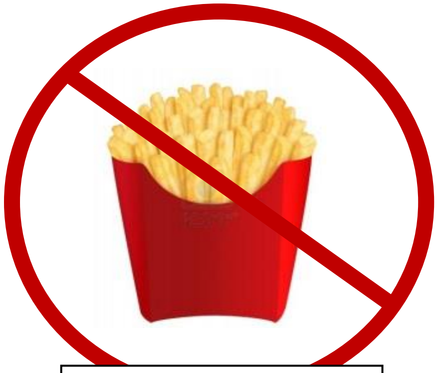

No Salty/Greasy Foods

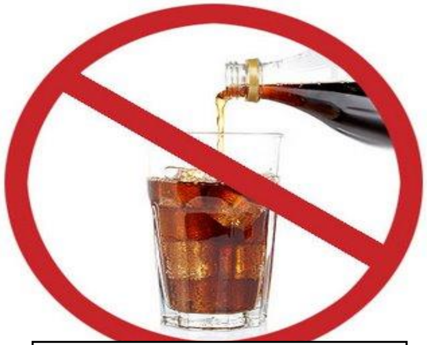

No Sugar Drinks

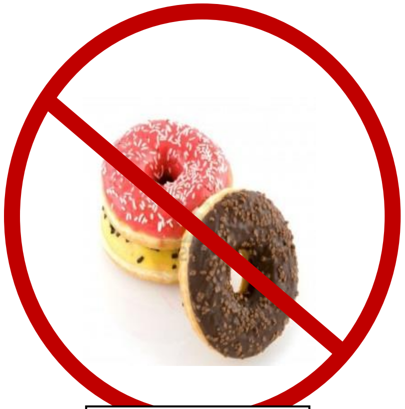

No Sweets

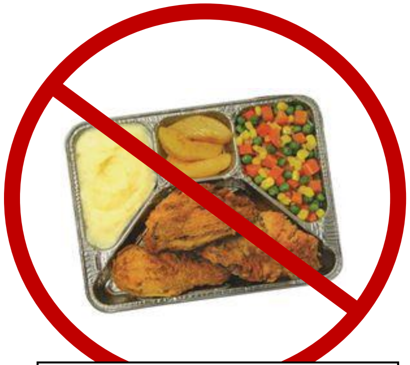

Avoid Processed Foods

Heart Health Strategies

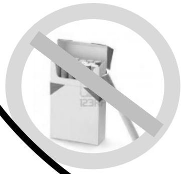

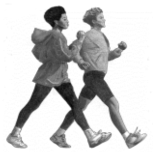

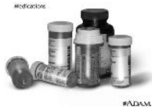

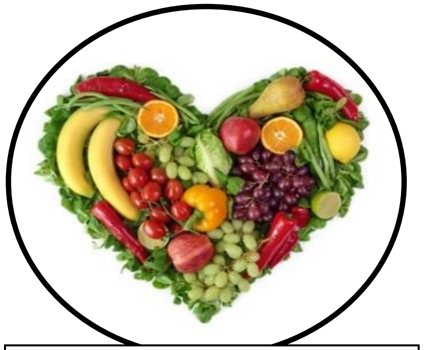

Eat More Fruits and Vegetables

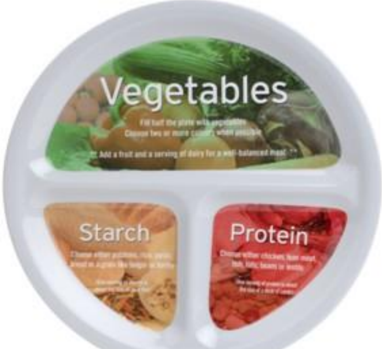

Eat Smart Portions

To identify focus of sessions with participants with diabetes, high blood pressure, and high cholesterol

© 2020 Daumit GL et al. *JAMA Netw Open.*

Name: ____________________________________
Week of: _________________________

IDEAL Tracker

DAY	MON	TUE	WED	THU	FRI	SAT	SUN
**Did you AVOID:**							
Sugar Drinks	Yes No	Yes No	Yes No	Yes No	Yes No	Yes No	Yes No
Salty/Greasy Foods	Yes No	Yes No	Yes No	Yes No	Yes No	Yes No	Yes No
Sweets	Yes No	Yes No	Yes No	Yes No	Yes No	Yes No	Yes No
Processed Foods	Yes No	Yes No	Yes No	Yes No	Yes No	Yes No	Yes No
**Did you EAT:**							
5 Fruits & Veggies	1 2 3 4 5	1 2 3 4 5	1 2 3 4 5	1 2 3 4 5	1 2 3 4 5	1 2 3 4 5	1 2 3 4 5
Smart Portions	Yes No	Yes No	Yes No	Yes No	Yes No	Yes No	Yes No
**Did you EXERCISE?**							
	Yes No	Yes No	Yes No	Yes No	Yes No	Yes No	Yes No

Weight:____________________lbs

© 2020 Daumit GL et al. *JAMA Netw Open.*

eTable 1. Trial Enrollment Criteria

**<u>Inclusion criteria</u>**
▪ Age 18 years and older
▪ Body mass index at least 25 kg/m^2 or<br>one of the following CVD risk factors:<br>-Hypertension (SBP>= 140mmHg or DBP>= 90mmHg or on antihypertensive medications;<br>-Diabetes mellitus (fasting blood sugar> 125mg/dl or hemoglobin A1c>6.5 or on a hypoglycemic medication);<br>-Dyslipidemia (LDL >130 mg/dl, HDL<40, total cholesterol >=200 or on a lipid lowering agent);<br>-Current tobacco smoker
▪ Able and willing to give informed consent
▪ Completion of baseline data collection
▪ Willing to accept randomization
▪ Willing to participate in the intervention
**<u>Exclusion criteria</u>**
▪ Cardiovascular event (unstable angina, myocardial infarction) within the past 6 months
▪ Serious medical condition which either limits life expectancy or requires active management (e.g., certain cancers)
▪ Condition which interferes with outcome measurement (e.g., dialysis)
▪ Pregnant or planning a pregnancy during study period. Nursing mothers would need approval from physician.
▪ Alcohol or substance use disorder if not sober/abstinent for 30 days
▪ Planning to leave rehabilitation center or clinic within 6 months or move out of geographic area within 18 months
▪ Investigator judgment (e.g., for concerns about participant or staff safety)

© 2020 Daumit GL et al. *JAMA Netw Open.*

eTable 2. Global Framingham Risk Score by Randomized Group at Each Visit (Cross-Sectional) and Model-Based Percent Longitudinal Change of Global Framingham Risk Score Over Time (corresponds to Figure 2 in text)

Global Framingham Risk Score	Intervention Group	Control Group	Intervention Group vs. Control
Baseline, n	132	137	
Mean	11.5 ± 11.5	12.7 ± 12.7	
Median	8.6 (3.9-16.0)	9.1 (4.0-16.7)	
6-month, n	123	126	
Mean	11.5 ± 12.3	12.5 ± 12.4	
Median	7.9 (3.3-14.3)	9.4 (3.4-17.5)	
18-month, n	124	132	
Mean	9.9 ± 10.2	12.3 ± 12.0	
Median	7.7 (3.1 -12.0)	9.7 (4.0-15.9)	
% change baseline to 6 mo.	-6.9 [-12.9, -0.8]	0.5 [-5.4, 6.5]	-7.4 [-15.9, 1.1]
% change baseline to 18 mo.	-11.2 [-18.5, -3.9]	1.4 [-5.7, 8.6]	-12.7 [-22.9, -2.5]

The global Framingham Risk Score reflects the 10-year probability of a cardiovascular event. Mean global Framingham Risk Score (± SD) and median, (Q1-Q3) ranges were calculated with cross-sectional data based on data available for the corresponding visit. N for intervention group at baseline is 132, 6 months is 123, 18 months is 124, N for control group at baseline is 137, 6 months is 126, 18 months is 132. Percentage changes reported are mean estimates and 95% CI derived from mixed-effects repeated measures analysis and correspond to Figure 2. The mixed-effects repeated measures analysis used all available data from all randomized participants, accounting for missing data based on missing at random mechanism.

© 2020 Daumit GL et al. *JAMA Netw Open.*

eFigure 1. Percent Change in ACC/AHA Risk Score Over Time According to Study Group

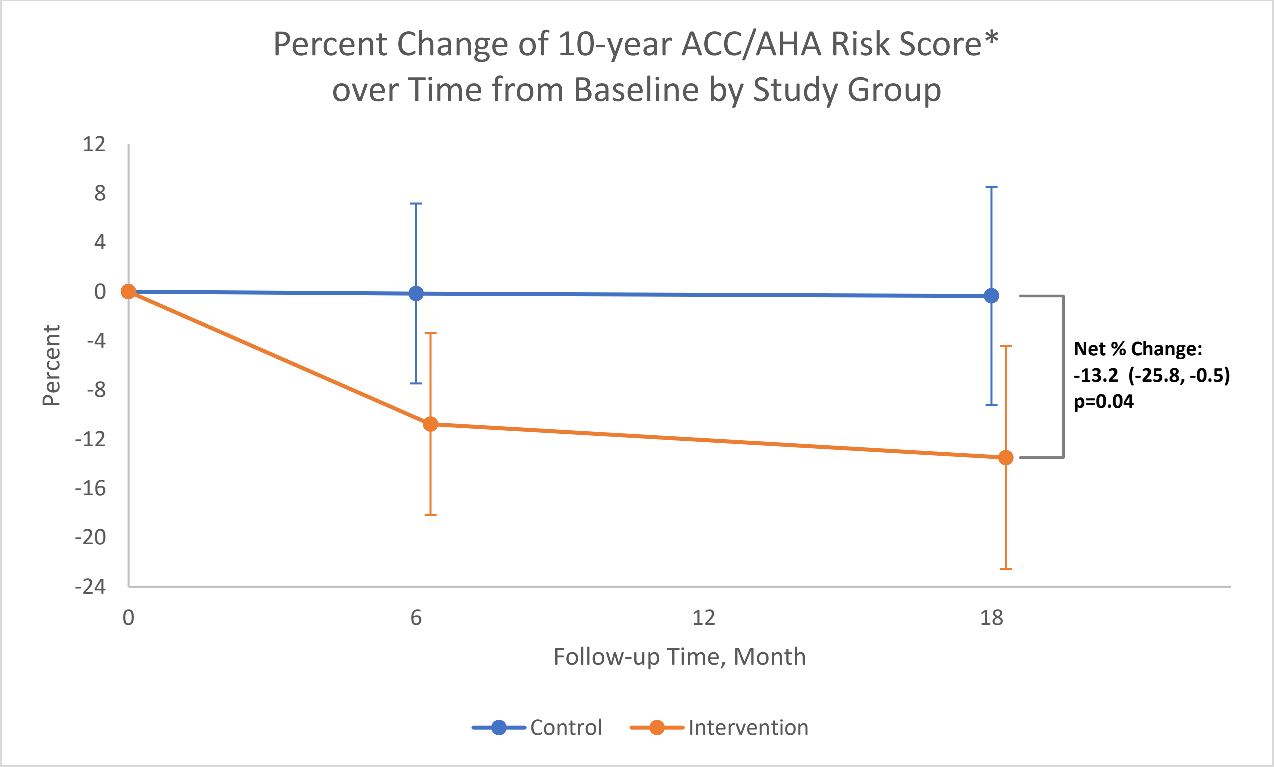

© 2020 Daumit GL et al. *JAMA Netw Open.*

eTable 3. ACC/AHA Risk Score by Randomized Group at Each Visit and Model-Based Percent Longitudinal Change of ACC/AHA Risk Score Over Time (corresponds to eFigure 1.)

ACC/AHA Risk Score	Intervention Group	Control Group	Intervention Group vs. Control Group
Baseline, n	132	137	
Mean	7.9 ± 7.8	9.4 ± 9.0	
Median	5.0 (1.9-11.9)	6.6 (2.7-12.5)	
6-month, n	123	126	
Mean	7.7 ±8.0	9.3 ± 9.3	
Median	4.9 (1.5-11.7)	6.9 (2.6-13.1)	
18-month, n	124	132	
Mean	7.0 ± 8.0	9.6 ± 10.4	
Median	4.9 (1.6-8.6)	6.1 (3.0-12.7)	
% change baseline to 6 mo.	-10.8 [-18.2,-3.4]	-0.1 [-7.5, 7.2]	-10.6 [-21.0, -0.2]
% change baseline to 18 mo.	-13.5 [-22.6,-4.4]	-0.3 [-9.2, 8.5]	-13.2 [-25.8, -0.5]

The AHA/ACC Risk Score reflects the 10-year probability of a cardiovascular event. Mean AHA/ACC Risk Score (± SD) and median, (Q1-Q3) ranges were calculated with cross-sectional data based on data available for the corresponding visit. N for intervention group at baseline is 132, 6 months is 123, 18 months is 124, N for control group at baseline is 137, 6 months is 126, 18 months is 132. % Changes reported are mean estimates and 95% CI derived from mixed-effects repeated measures analysis and correspond to eFigure 1. The mixed-effects repeated measures analysis used all available data from all randomized participants, accounting for missing data based on missing at random mechanism.

© 2020 Daumit GL et al. *JAMA Netw Open.*

eFigure 2. Model-Based Percent Change in Global Framingham Risk Score by Subgroups of Sex, Race, Cardiovascular Risk at Baseline, and Psychiatric Diagnosis

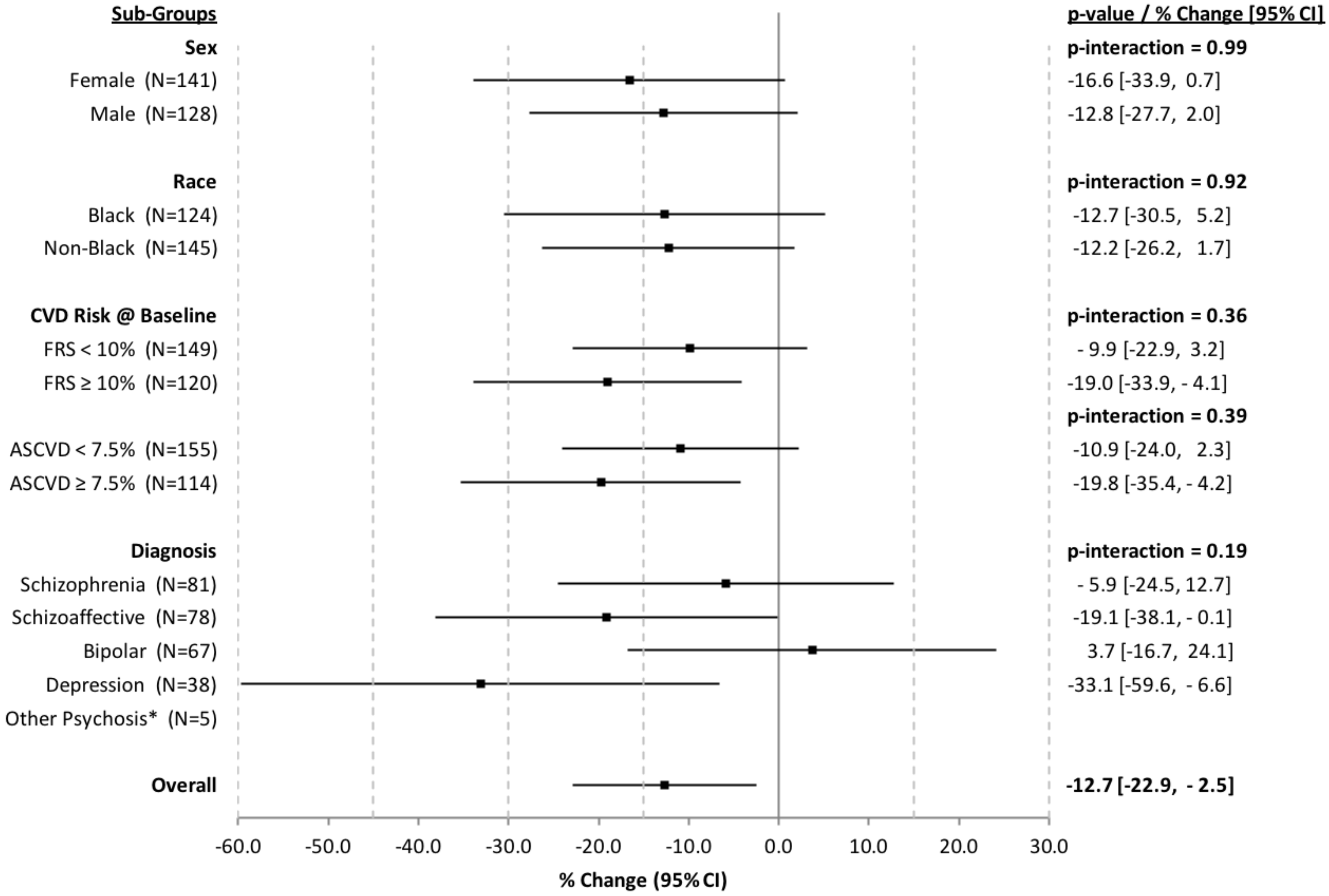

*Small sub-group (N=5), estimates are not displayed

Estimates are derived from mixed-effects repeated measures analysis using all available data from all randomized participants. ASCVD refers to the ACC/AHA risk score.

© 2020 Daumit GL et al. *JAMA Netw Open.*

eTable 4. Overnight Hospitalizations, Deaths and Medical Events for Intervention and Control Participants.

4a. Overnight Hospitalizations

	Intervention		Control	
	Number of participants	Number of events	Number of participants	Number of events
Medical hospitalizations	20	22	30	41
Psychiatric hospitalizations	10	14	9	13
Total hospitalizations	29	36	35	54

Overnight hospitalization data are from standard data collection visits.

4b. Deaths

	Intervention	Control
Deaths	1 unknown reason<br>1 presumed cardiac death	1 unknown reason<br>1 cardiac arrest
Total	2	2

4c. Medical Events

	Intervention	Control
Cardiovascular		
Heart Attack & PTCA	1	0
PTCA	0	1
Arrhythmia	1	0
Pacemaker / ICD device	1	0
Lower extremity bypass surgery	0	1
TIA	0	1
Stroke	0	1
Hypertension	0	1
Hypotension	1	0
DVT	1	0
Edema	0	1
Total	5	6
Diabetes mellitus		
Hypoglycemia	0	1
Hyperglycemia	0	1
Total	0	2
Orthopedic/Musculoskeletal		
Hip surgery	0	2
Back surgery	1	2
	Intervention	Control
Total	1	4

© 2020 Daumit GL et al. *JAMA Netw Open.*

Gastrointestinal		
Bowel Obstruction	1	1
Stomach Surgery	0	1
Pancreatitis	0	1
Hyponatremia/Dehydration	2	1
Other Gastrointestinal	3	3
Total	6	7
Respiratory		
COPD	0	2
Pneumonia	1	5
Respiratory distress	0	1
Respiratory failure	0	1
Difficulty breathing	2	1
Bronchitis	2	1
Total	5	11
Neurological		
Concussion	0	1
Seizure	0	1
Tremors	1	0
Total	1	2
Other Medical Events		
Skin infection	1	0
Viral infection	0	1
Tonsillectomy	0	1
Thyroidectomy	0	1
Urinary surgery	0	1
Post-surgical bleeding	0	2
Cancer	1	0
Ear pain	1	0
Fall	1	3
Total	4	9
Overall Total	22	41

Medical event data are from standard data collection visits.

© 2020 Daumit GL et al. *JAMA Netw Open.*
