# Supplementary material for: Effect of a Comprehensive Cardiovascular Risk Reduction Intervention in Persons With Serious Mental Illness: A Randomized Clinical Trial
Source: JAMA Netw Open. 2020 Jun 12;3(6):e207247. doi: 10.1001/jamanetworkopen.2020.7247 (PMC7293000; doi:10.1001/jamanetworkopen.2020.7247)
Supplement: Supplement 3. — Data Sharing Statement [file jamanetwopen-3-e207247-s003.pdf]

Data Sharing Statement

Daumit. Effect of a Comprehensive Cardiovascular Risk Reduction Intervention in Persons With Serious Mental Illness. *JAMA Netw Open*. Published June 12, 2020. 10.1001/jamanetworkopen.2020.7247

Data
Data available: Yes
Data types: Deidentified participant data, Data dictionary
How to access data: gdaumit@jhmi.edu
When available: beginning date: 03-01-2021

Supporting Documents
Document types: None

Additional Information
Who can access the data: Researchers who proposed use of the data has been approved
Types of analyses: for research
Mechanisms of data availability: with a signed data access agreement and IRB approval
